# Synthesis, anti-inflammatory, bactericidal activities and docking studies of novel 1,2,3-triazoles derived from ibuprofen using click chemistry

**DOI:** 10.1186/s40064-016-2052-5

**Published:** 2016-04-11

**Authors:** Kishore Kumar Angajala, Sunitha Vianala, Ramesh Macha, M. Raghavender, Murali Krishna Thupurani, P. J. Pathi

**Affiliations:** Department of Chemistry, University College of Science, Saifabad, Osmania University, Hyderabad, Telangana 500004 India; Chaitanya College of Pharmacy Education and Research, Kishanpura, Hanamkonda, Warangal, Telangana 506001 India

**Keywords:** Click chemistry, Triazoles, Ibuprofen, Anti-inflammatory, Docking, Bactericidal

## Abstract

**Background:**

Nonsteroidal anti-inflammatory drugs are of vast therapeutic benefit in the treatment of different types of inflammatory conditions. 1,2,3-Triazoles and their derivatives have a wide range of applications as anti-bacterial, anti-fungal, anti-tubercular, cytostatic, anti-HIV, anti-allergic, anti-neoplastic and anti-inflammatory (AI) agents. Considering the individual biological and medicinal importance of ibuprofen and 1,2,3-triazoles, we wanted to explore novel chemical entities based on ibuprofen and triazole moieties towards their biological significance.

**Results:**

Click chemistry has utilized as an ideal strategy to prepare novel ibuprofen-based 1,4-disubstituted 1,2,3-triazole containing molecules. These compounds were screened for their in vivo AI activity, among all the synthesized analogues **13o** was shown potent effect than the reference AI drug ibuprofen at the same concentration (10 mg/kg body weight). Compounds **13l**, **13g**, **13c**, **13k**, **13i**, **13n**, **13m** and **13j** were shown significant AI activity. These triazole analogues were also screened for their bactericidal profile. Compounds **13c**, **13i**, **13l** and **13o** exhibited considerable bactericidal activity against gram positive and gram negative strains. In addition to this, molecular docking studies were also carried out into cyclooxygenase-2 active site to predict the affinity and orientation of these novel compounds (**13a**–**q**).

**Conclusions:**

In summary, we have designed and synthesized 1,2,3-triazole analogues of ibuprofen in good yields using Click chemistry approach. AI and bactericidal activities of these compounds were evaluated and shown remarkable results.

**Electronic supplementary material:**

The online version of this article (doi:10.1186/s40064-016-2052-5) contains supplementary material, which is available to authorized users.

## Background

The nonsteroidal anti-inflammatory drugs are extensively applied for the treatment of analgesic, antipyretic, rheumatic arthritis and in high doses these are used to treat inflammatory diseases. Prolonged oral administration of these drugs was reported for frequent adverse effects on the gastrointestinal tract (GIT) (Allison et al. 1992; Lazzaroni and Bianchi Porro 2004) and subsequently leads to obstacles such as kidney damage (Ruiz and Lowenthal 1997), gastric ulcer (Alsarra et al. 2010) and hepatotoxicity (Tan et al. 2007). This is most likely due to the presence of free carboxyl group on Nonsteroidal anti-inflammatory drugs (NSAIDs) (Mishra et al. 2008). The GIT mucosal injury problems produced by NSAIDs are commonly believed to be caused by two different mechanisms. One is local irritation produced by free carboxylic acid group and inhibition of prostaglandin biosynthesis in the GIT. The second has indirect effect can be attributed to combination of an ion trapping mechanism of NSAIDs from the lumen into the mucosa. Thus, free acidic group plays a key role in keeping the effectiveness and producing the gastric ulceration as well. Hence, there remains a compelling need for effective NSAIDs with an improved safety profile and strategy for suppressing inflammation with least side effects.

It has been reported (Kalgutkar et al. 2000a, b, 2002; Shanbhag et al. 1992; Tozkoparan et al. 2000) that conversion of the carboxyl group containing NSAIDs to ester, amide functions and some other modifications retains the anti-inflammatory activity of the parent NSAIDs and makes them more selective towards cyclooxygenase-2 (COX-2) enzyme. These modifications were inspired us to concentrate on the carboxyl side chain of ibuprofen to design novel class of molecules. During the synthesis of these analogues, we planned to utilize Click chemistry as key reaction. Click reaction is one of the most popular reactions for the construction of triazoles. Concept of this reaction discovered by the groups of Sharpless (Rostovtsev et al. 2002) and Meldal (Tornøe et al. 2002) independently. It is a copper(I)-catalyzed 1,3-dipolar cycloaddition (CuAAC) reaction which involves alkyne and azide as key partners to deliver 1,2,3-triazoles. Click chemistry has recently emerged to become a powerful tool in drug discovery. The 1,4-disubstituted 1,2,3-triazoles obtained from CuAAC reactions are found to possess wide applications in several research fields including synthetic organic (Liu et al. 2008; Wacharasindhu et al. 2009), biological (Romeo et al. 2015) and medicinal chemistry (Liang et al. 2015). 1,2,3-Triazole is one of the key structural unit found in a wide variety of bioactive molecules tazobactam (Yang et al. 1999), cefatrizine (Dunn et al. 1976), carboxyamidotriazole (Guo et al. 2008).

**Fig. 1:**
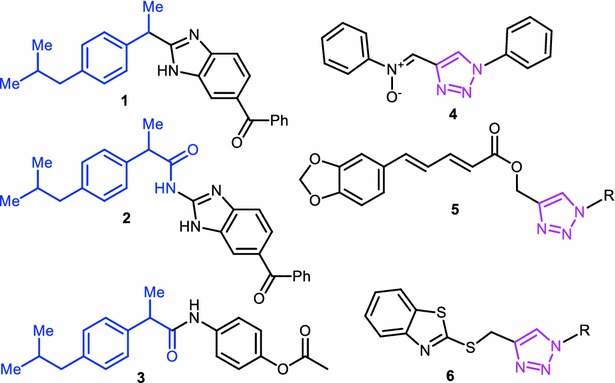
Examples of COX-2 inhibitors/anti-inflammatory molecules containing ibuprofen and triazole moieties

**Fig. 2:**
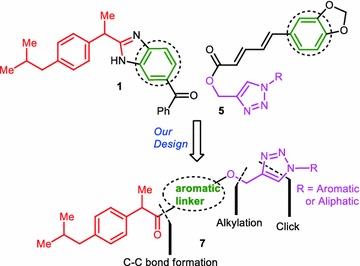
Design of novel molecules **7** from **1** and **5** containing ibuprofen-aromatic linker-triazole moieties and their key disconnections

In this paper, we framed out our studies to synthesize analogues of ibuprofen, a widely used drug among other clinically existing NSAIDs. Free carboxyl group in ibuprofen could be modified to obtain more potent analogues. Derivatives of ibuprofen with modified carboxylic acid functionality were shown in Fig. 1 (**1**–**3**) (Bansal et al. 2015; Yadav et al. 2006). In compound **1**, carboxyl group modified as an aromatic motif. Whereas in compounds **2**–**3**, acid functionality diminished by making different substituted aromatic amides. Interestingly, we found that some molecules having 1,2,3-triazoles (**4**–**6**, Fig. 1) (Rao et al. 2014; Haftchenary et al. 2015; Shafi et al. 2012) also showed good anti-inflammatory properties. Considering the individual biological activities, medicinal importance of ibuprofen and 1,4-disubstituted 1,2,3-triazoles, we designed novel class of small molecules (**7**, Fig. 2) having these two pharmacophores in single frame work through an aromatic linker. Resorcinol could be a useful moiety to link ibuprofen and 1,2,3-triazoles via C–C and C–O bonds respectively. The more active position (4th) of resorcinol was linked to carboxyl group of ibuprofen via C–C bond to obtain a novel compound which having ibuprofen as a major structural backbone and key resorcinol moiety. We utilized more reactive hydroxyl functionality, which is present at *para* position of the linker to make the propargyl handle. Using Click chemistry approach, this was further diversified by treating with different azides to give novel analogues that contain ibuprofen, resorcinol and 1,4-disubstituted 1,2,3-triazoles as substructures.

In recent years, the multidrug resistance of microbial pathogens has heightened the urgency to develop new antibacterial agents. Having the advantages of mono therapy of an anti-inflammatory drug with anti-microbial properties, here we studied bactericidal activity along with anti-inflammatory activity of the newly synthesized triazoles.

## Results and discussion

### Chemistry

**Scheme 1:**
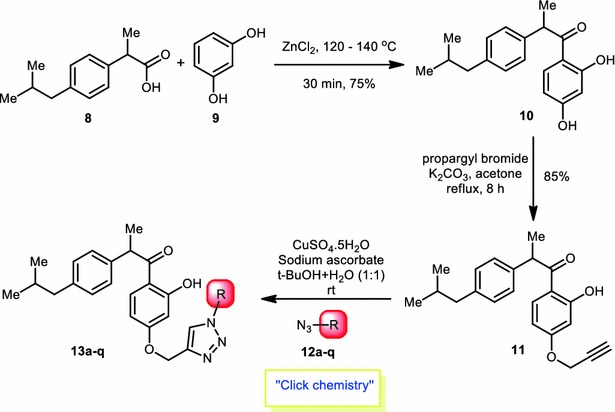
Synthesis of 1-(4-((1*H*-1,2,3-triazol-4-yl)methoxy)-2-hydroxyphenyl)-2-(4-isobutylphenyl)propan-1-one derivatives **13a**–**q**

As shown in Scheme 1, 1-(2,4-dihydroxyphenyl)-2-(4-isobutylphenyl)propan-1-one (**10**) was prepared by heating of resorcinol (**9**) and ibuprofen (**8**) in the presence of freshly fused ZnCl_2_. For *O*-alkylation, compound **10** was refluxed with propargyl bromide and potassium carbonate in dry acetone for 8 h. In this reaction we got *para* propargylated compound (**11**) as major product with 85 % yield. The reason for formation of *para* propargylated product as major may be explained on the basis of mesomeric effect and steric factors; *ortho* hydroxy group will have less nucleophilicity than *para* hydroxyl group. Major product (**11**) was separated and analyzed by ^1^H-NMR spectroscopy, which showed characteristic singlet at δ 12.91 due to presence of chelated phenolic hydrogen of *ortho* hydroxyl group and singlet for two protons at δ 4.66 (O–CH_2_–), triplet for one proton at δ 2.53 (≡CH) indicates the formations of O-propargylation. With this evidence it is confirmed that the resulting major product was 1-(2-hydroxy-4-(prop-2-yn-1-yloxy)phenyl)-2-(4-isobutylphenyl)propan-1-one (**11**).

**Fig. 3:**
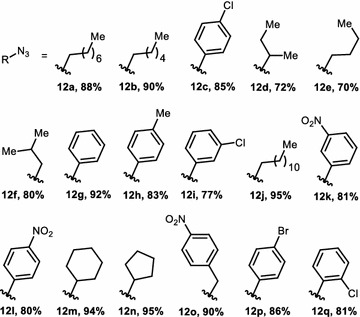
Different aliphatic and aromatic azides (**12a**–**q**)

Then we synthesized various aliphatic and aromatic azides (**12a**–**q**, Fig. 3) by utilizing literature protocols (Lee et al. 2012; Kumar et al. 2014). To prepare alkyl azides, corresponding alkyl halide was heated at 80–90 °C with NaN_3_ in DMF. Aromatic azides were prepared from different substituted anilines using diazotization followed by treatment with NaN_3_.

**Fig. 4:**
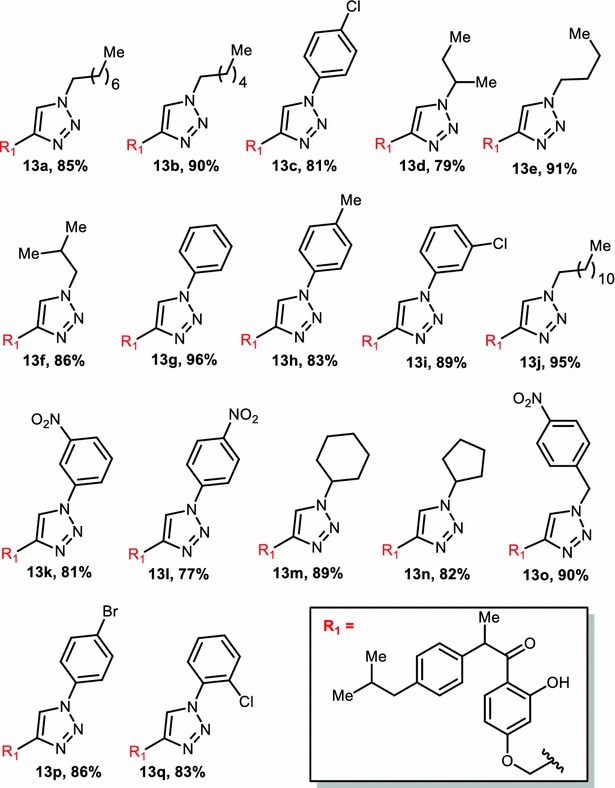
Derivatives of target compound (**13a**–**q**)

1,3-diploar cycloaddition between 1-(2-hydroxy-4-(prop-2-yn-1-yloxy)phenyl)-2-(4-isobutylphenyl)-propan-1-one (**11**) and various aromatic, aliphatic azides (**12a**–**q**) produced seventeen novel 1,4-disubstituted 1,2,3-triazoles in good to excellent yields (**13a**–**q**, Fig. 4). All the synthesized compounds were thoroughly analyzed by ^1^H-NMR, ^13^C-NMR and LRMS analytical techniques. Purity was determined by HPLC using the condition specified in each case: column, mobile phase, flow rate (Additional file 1).

## Biological study

### In vivo anti-inflammatory activity

The in vivo anti-inflammatory activity of the synthesized novel triazole moiety containing molecules (**13a**–**q**) was determined at the dose of 10 mg/kg body weight using carrageenan-induced rat paw edema model (Winter et al. 1962). Anti-inflammatory activity was calculated at hourly intervals up to 6 h after injection and results were summarized in Table 1 as the mean ± SEM paw volume (mL) and the percentage anti-inflammatory activity. The paw volume differences were compared between the treated animals and the control group. Percentage inhibition was calculated as per the formula, % inhibition = [(V_o_ − V_t_)/V_o_] × 100, where V_o_ = volume of the paw control at time t, V_t_ = volume of the paw of drug treated at time t. The maximum anti-inflammatory activity was obtained after 3 h which is the time required for reaching the maximum activity, soon after gradually decreased for the next 2 h.

**Table 1:** *In vivo* anti-inflammatory activity of novel 1,2,3-triazoles (**13a**–**q**)

Compound	3 h		4 h		5 h		6 h	
	Swelling	Inhibition (%)	Swelling	Inhibition (%)	Swelling	Inhibition (%)	Swelling	Inhibition (%)
Volume of edema^c^ (mL) and % AI^d^								
Control (−)	1.17 ± 0.032		1.37 ± 0.012		1.60 ± 0.019		1.89 ± 0.009	
Ibuprofen	0.08 ± 0.021	93.16	0.06 ± 0.027	95.62	0.07 ± 0.027	95.62	0.10 ± 0.021	94.70
**13a**	0.60 ± 0.015	48.17	0.60 ± 0.004	56.20	0.66 ± 0.004	58.75	0.71 ± 0.012	62.43
**13b**	0.62 ± 0.024^a^	47.00	0.61 ± 0.008^a^	55.47	0.69 ± 0.008^a^	56.87	0.74 ± 0.016^a^	60.84
**13c**	0.17 ± 0.020	85.47	0.17 ± 0.016	87.59	0.21 ± 0.009	86.87	0.26 ± 0.011	86.24
**13d**	0.50 ± 0.007	57.26	0.49 ± 0.019	64.23	0.54 ± 0.009	66.25	0.56 ± 0.015	70.37
**13e**	0.43 ± 0.011	63.24	0.42 ± 0.023	69.34	0.53 ± 0.013	66.87	0.62 ± 0.018	67.19
**13f**	0.54 ± 0.015	53.84	0.54 ± 0.010	60.58	0.57 ± 0.019	64.37	0.62 ± 0.011	67.19
**13g**	0.15 ± 0.017^a^	87.17	0.12 ± 0.007^a^	91.24	0.15 ± 0.014^a^	90.62	0.20 ± 0.012^a^	89.41
**13h**	0.40 ± 0.022	65.81	0.49 ± 0.012	64.23	0.56 ± 0.017	65.00	0.60 ± 0.011	68.25
**13i**	0.23 ± 0.009^b^	80.34	0.20 ± 0.019^b^	85.40	0.26 ± 0.010^b^	83.75	0.31 ± 0.016^b^	83.59
**13j**	0.32 ± 0.012^b^	72.64	0.34 ± 0.023^b^	75.18	0.40 ± 0.013^b^	75.00	0.49 ± 0.011^b^	74.07
**13k**	0.20 ± 0.010^b^	82.90	0.19 ± 0.016^b^	86.13	0.25 ± 0.012^b^	84.37	0.30 ± 0.020^b^	84.20
**13l**	0.11 ± 0.019^a^	90.59	0.09 ± 0.011^a^	93.43	0.11 ± 0.021^a^	93.12	0.16 ± 0.012^a^	91.53
**13m**	0.27 ± 0.024^b^	76.92	0.26 ± 0.020^b^	81.02	0.32 ± 0.011^b^	80.00	0.42 ± 0.017^b^	77.77
**13n**	0.25 ± 0.006^b^	78.63	0.21 ± 0.012^b^	84.67	0.27 ± 0.021^b^	83.12	0.35 ± 0.009^b^	81.48
**13o**	0.07 ± 0.011^a^	94.01	0.05 ± 0.021^a^	96.35	0.07 ± 0.011^a^	95.62	0.11 ± 0.014^a^	94.17
**13p**	0.36 ± 0.010	69.23	0.40 ± 0.015	70.80	0.47 ± 0.010	70.62	0.52 ± 0.012	72.48
**13q**	0.38 ± 0.019	67.52	0.45 ± 0.019	67.15	0.26 ± 0.049^b^	67.50	0.58 ± 0.020	69.31

^a^P < 0.001 and ^b^ P < 0.01. Control (−) (0.1 mL of saline solution)

^c^Values are expressed as mean ± SEM from six observations and data is analyzed by one way ANOVA followed by Dunnett’s ‘t’ test

^d^Values in parentheses [percentage anti-inflammatory activity (% AI)]

Compounds **13a**–**q**, showed mild to excellent anti-inflammatory activities (47.00–94.01 % at 3 h, 55.47–96.35 % at 4 h and 56.87–95.62 % at 5 h). It is interesting to note that presence of electron withdrawing group or atom (NO_2_ or Cl) at *meta* or *para* (C_3_ or C_4_) positions of benzyl or phenyl ring on triazole leads to significant increase in the activity. Among these triazoles (**13a**–**q**), compound **13o** bearing a 4-nitrobenzyl group on the triazole moiety exhibited most potent activity 94.01 % at 3 h, 96.35 % at 4 h, 95.62 % at 5 h and 94.17 % at 6 h with compare to reference drug (93.16 % at 3 h, 95.62 % at 4 h, 95.62 % at 5 h and 94.70 % at 6 h). Compound **13l** bearing a 4-nitrophenyl group on the triazole moiety showed good anti-inflammatory activity of 90.59 % at 3 h, 93.43 % at 4 h and 93.12 % at 5 h. The 1,4-disubstituted 1,2,3-triazole nucleus bearing phenyl (**13g**), 4-chloro phenyl (**13c**) were shown considerable inhibition of edema 91.24 and 87.59 % respectively at 4 h. Moderate activity was observed with respect to compounds **13k** (86.13 %), **13i** (85.40 %), **13n** (84.67 %) and **13m** (81.02 %) at 4 h.

### Bactericidal activity

According to the results obtained, all the bacterial strains noticed high susceptible nature towards the compounds tested. Among the tested triazoles, compounds **13c**, **13i**, **13l** and **13o** exhibited high bactericidal activity. The minimum inhibitory concentration (MIC) of **13o** against tested bacterial strains is comparable with that from standard antibiotic drug cefixime (Table 2). On the other hand, compounds **13l**, **13c** and **13i** also produced significant MIC and minimum bactericidal concentration (MBC) values against tested human pathogenic organisms. These results indicates that electron withdrawing group or atom (NO_2_ or Cl) at *meta* or *para* positions of benzyl or phenyl ring attached to triazole may increasing the bactericidal activity than aliphatic and electron donating aryl substituted triazoles. By the present investigation, it has been understood that the synthesized compounds are highly active against gram positive strain compared to gram negative strains. Among the screened bacterial strains methicillin-resistant *Staphylococcus aureus* (MRSA) was found most susceptible MIC (12.5) and MBC (15.1) values for compound **13o** which were almost nearer to the values <13.5 and <13 of positive control.

**Table 2:** MIC/MBC (µg/mL) values of synthesized compounds (**13a**–**q**) and Cefixime against tested bacterial strains

Compounds	Gram positive						Gram negative					
	MRSA		*B. subtilis*		*B. cereus*		*E. coli*		*K. pneumoniae*		*P. vulgaris*	
	MIC	MBC	MIC	MBC	MIC	MBC	MIC	MBC	MIC	MBC	MIC	MBC
**13a**	>150	>155	109.3	112	118	120	98.6	100	>130	>140	>90	>90
**13b**	120	126	88.2	>90	102	108	92.6	100	>128	>140	>100	>110
**13c**	24.1	27.3	28.9	>40	32.0	42.1	28.6	30.2	38.5	>40	>50	>50
**13d**	30.6	31.6	>25	30.2	45.7	50.1	>40	42.3	62.8	65.2	78.3	80.2
**13e**	57.9	60.0	60.5	70.2	79.6	82.1	>120	>120	>100	105.1	>150	>150
**13f**	>170	>170	>180	>180	88.1	90.2	112	117	136	138	80.3	85
**13g**	40.5	42.3	66.6	70.5	>60	68.0	55.3	55.5	33.8	36.9	90.1	>100
**13h**	>118	>120	>140	>140	114	117	>130	<135	175	179	<200	<200
**13i**	20.5	22.1	18.6	19.3	25.8	30.3	22.5	25.8	35.9	38.0	45.0	>50
**13j**	>188	>190	>153	>160	>160	>160	112	113	>145	>148	>170	>172
**13k**	33.6	>40	35.9	47.0	44.6	50.2	>50	66.0	58.2	65.0	>80	>86
**13l**	18.5	24.9	20.5	22.8	20.7	21.0	18.2	24.1	33.6	35.1	30.9	33.5
**13m**	>189	>200	>168	>170	159	163	149	156	177	180	161	164
**13n**	36.3	39.5	58.0	>80	>60	>75	>50	58.6	80.2	92.0	>70	>85
**13o**	12.5	15.1	12.9	13.0	12.0	12.0	15.5	20.3	25.3	>30	28.4	>35
**13p**	>120	>120	98.9	102	>140	>150	80.6	92.8	>100	>120	>150	>165
**13q**	>125	>130	>155	158	>160	>160	>110	>115	146	148	133	134
Cefixime	<13.5	<13	10.2	11.0	10	10	13.0	13	17	17.2	20.6	21.0

*MRSA*, Methicillin-resistant *Staphylococcus aureus*, *B. subtilis*, *Bacillus subtilis*, *B. cereus*, *Bacillus cereus*, *E. coli*, *Escherichia coli*, *K. pneumoniae*, *Klebsiella pneumonia*, *P. Vulgaris*, *Proteus vulgaris*

### Molecular modeling approach

**Fig. 5:**
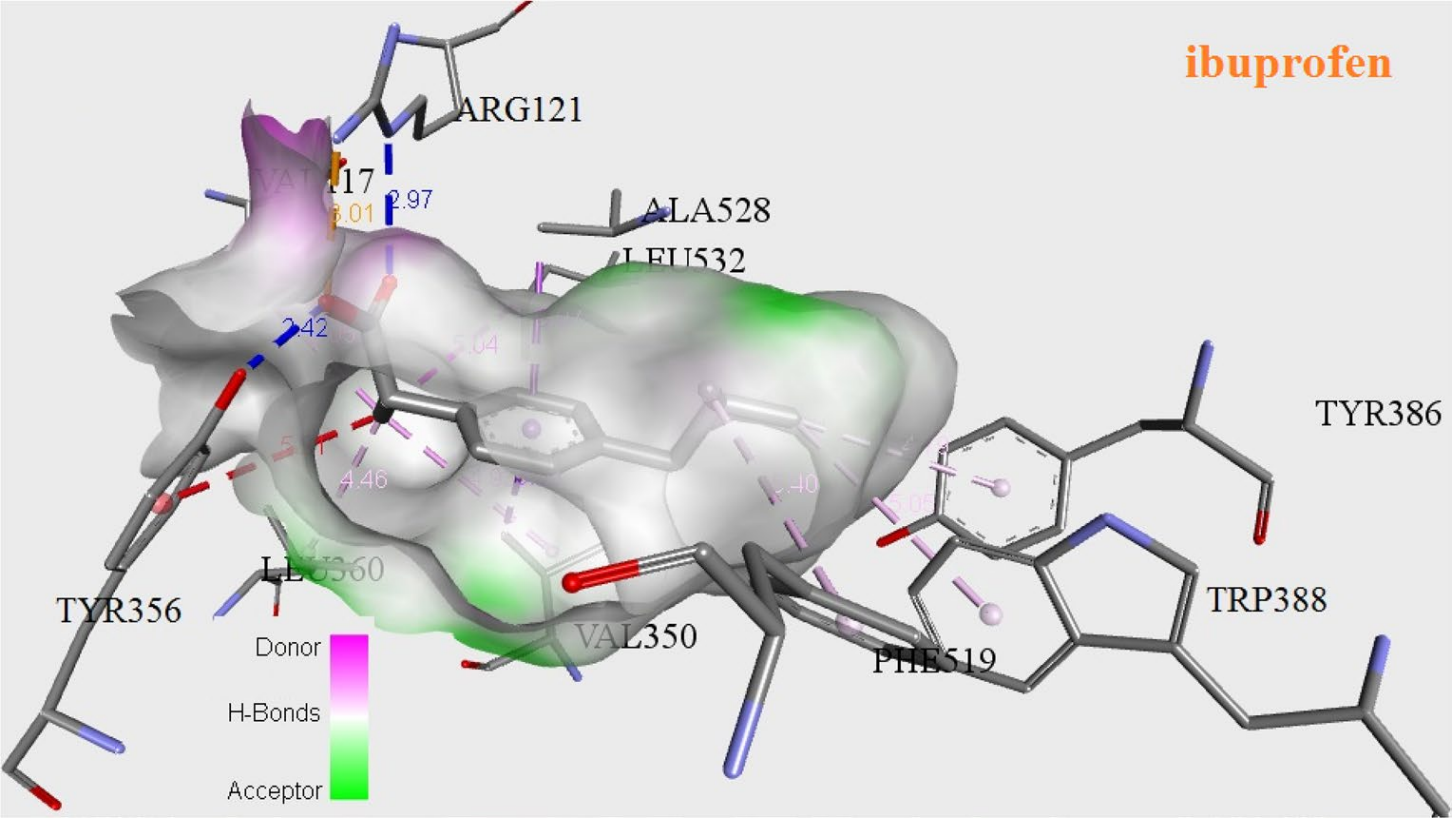
Docking pose of ibuprofen into the COX-2 (4PH9) active site. Hydrogen bonds are shown in *dotted lines*

**Fig. 6:**
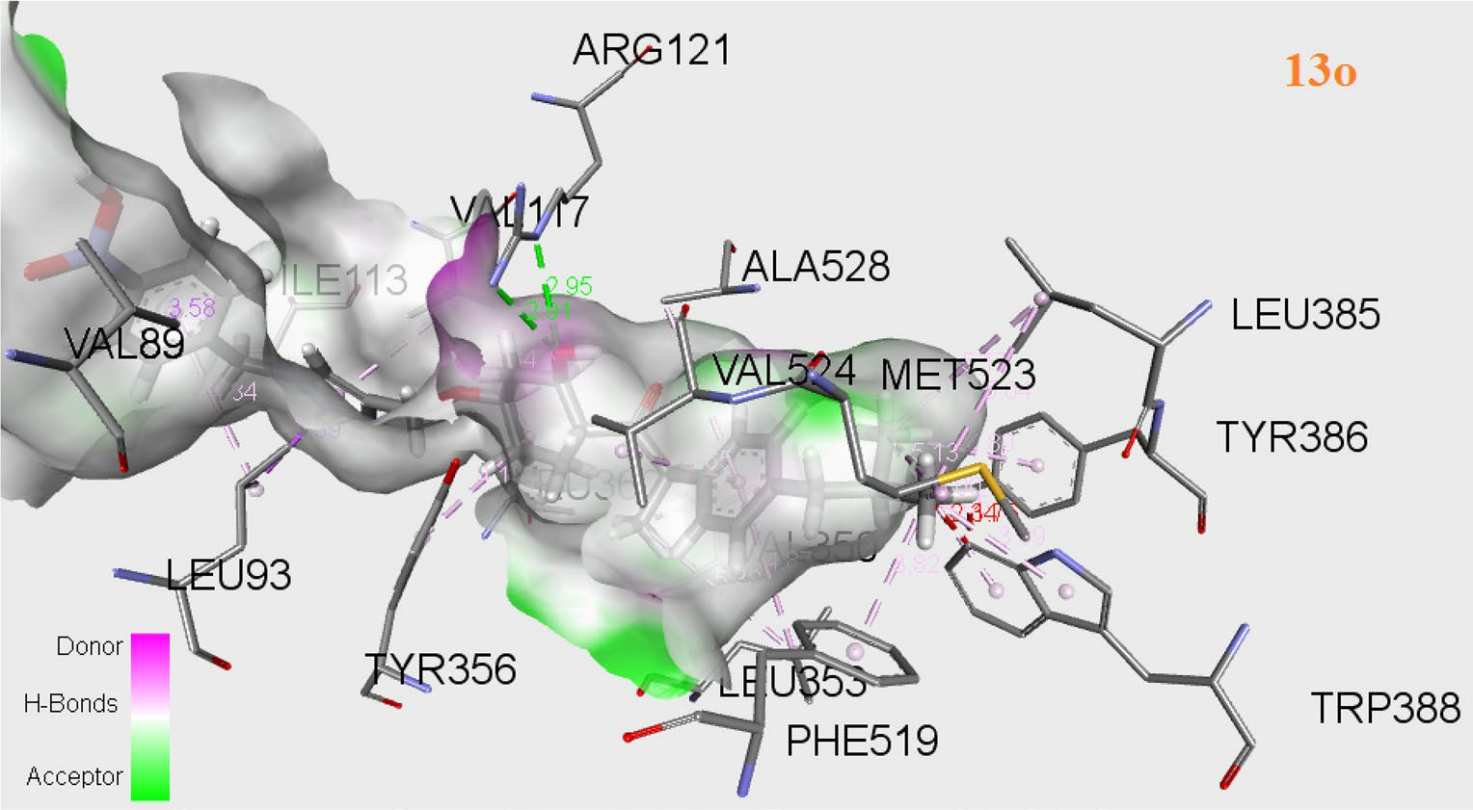
Docking pose of compound **13o** into the COX-2 (4PH9) active site. Hydrogen bonds are shown in *dotted lines*

**Fig. 7:**
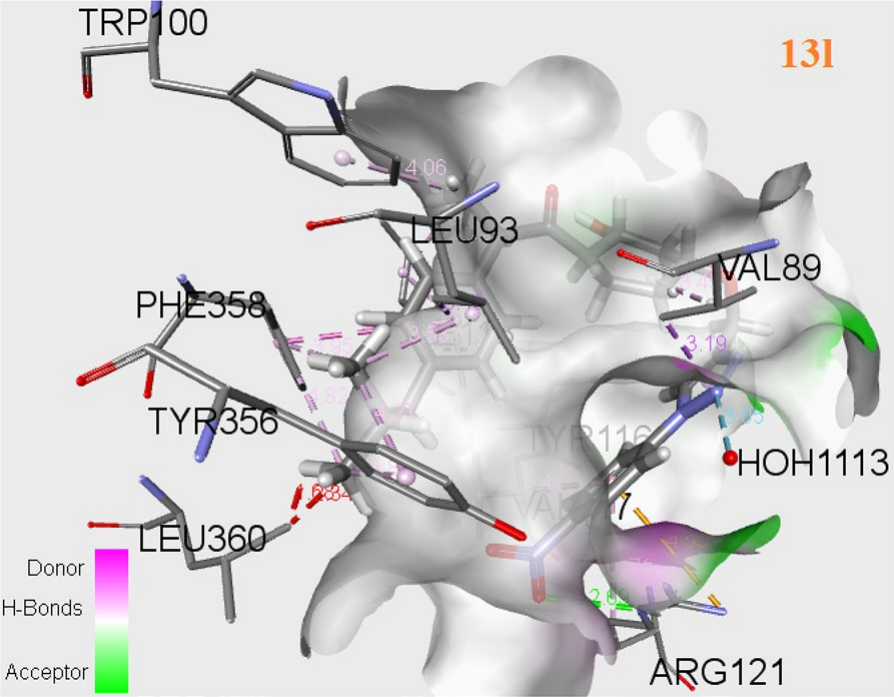
Docking pose of compound **13l** into the COX-2 (4PH9) active site. Hydrogen bonds are shown in *dotted lines*

The successful docking has been performed for all newly synthesized target compounds (**13a**–**q**) using genetic optimization for ligand docking (GOLD) algorithm version 2.0 (Verdonk et al. 2003). The GOLD program uses a genetic algorithm (GA) to explore the full range of the rotational flexibility of selected receptor hydrogens and ligand flexibility. The 3D crystallographic structure of COX-2 (PDB code 4PH9) (Orlando et al. 2015) was used as template selected from RCSB protein data bank (PDB) for anti-inflammatory activity. The interactions between ligand (**13a**–**q**) and receptor in the modeled complexes were investigated and observed the fitness function ability of COX-2 by all newly synthesized inhibitors. The observed Chem score and Gold fitness scores have been produced in Tables 3 and 4. Binding energies shown in Table 5 were calculated with ArgusLab (Thompson 2004) docking software. Discovery studio visualizer has been utilized to visualize the binding conformations of these analogues in the active site of 4PH9 protein and good binding orientation poses were shown in Figs. 5, 6 and 7.

**Table 3:** Chem score of novel 1,2,3-triazoles (**13a**–**q**)

Compound	Score	DG	S(hbond)	S(metal)	S(lipo)	DE(clash)	DE(int)
**13a**	33.75	−38.96	1.77	0.00	290.03	0.79	4.43
**13b**	32.60	−36.75	2.67	0.00	244.12	0.75	3.40
**13c**	40.43	−45.28	1.94	0.00	342.10	0.75	4.10
**13d**	33.17	−37.99	2.69	0.00	251.40	2.10	2.72
**13e**	36.23	−40.09	3.56	0.00	246.17	0.11	3.75
**13f**	31.89	−35.41	2.70	0.00	229.21	0.09	3.43
**13g**	38.65	−42.06	3.58	0.00	257.62	1.20	2.20
**13h**	36.19	−40.46	3.52	0.00	246.84	2.42	1.85
**13i**	38.03	−42.37	3.58	0.00	260.74	1.87	2.47
**13j**	36.76	−40.21	2.34	0.00	277.60	0.28	3.18
**13k**	38.84	−41.32	2.98	0.00	267.56	0.08	2.40
**13l**	38.17	−41.17	1.94	0.00	297.19	0.47	2.52
**13m**	33.96	−40.00	2.91	0.00	265.74	2.71	3.32
**13n**	35.60	−39.63	2.61	0.00	271.34	0.05	3.99
**13o**	35.66	−38.89	2.39	0.00	277.42	0.46	2.76
**13p**	39.07	−47.49	2.12	0.00	346.07	3.77	4.65
**13q**	38.39	−42.34	3.52	0.00	261.98	0.56	3.39
Ibuprofen	21.39	−22.32	2.05	0.00	132.11	0.10	0.84

Chem score = Δ*G*_binding_ + *P*_clash_ + *C*_internal_ *P*_internal_ + (*C*_covalent_ *P*_covalent_ + *P*_constraint_)

Score = −[DG + DE(clash) + DE(int)]

**Table 4:** Gold fitness score of novel 1,2,3-triazoles (**13a**–**q**)

Compound	Fitness	S(hb_ext)	S(vdw_ext)	S(hb_int)	S(vdw_int)
**13a**	45.77	1.16	43.59	0.00	−15.31
**13b**	57.40	7.50	47.91	0.00	−15.97
**13c**	54.17	4.29	56.67	0.00	−28.04
**13d**	55.57	12.48	44.01	0.00	−17.43
**13e**	51.45	5.51	44.29	0.00	−14.96
**13f**	58.76	7.97	46.45	0.00	−13.09
**13g**	60.01	6.00	48.17	0.00	−12.22
**13h**	56.08	9.08	47.70	0.00	−10.33
**13i**	56.08	4.69	47.22	0.00	−13.53
**13j**	55.11	7.17	45.42	0.00	−14.52
**13k**	52.03	5.07	44.07	0.00	−13.63
**13l**	54.78	6.10	49.52	0.00	−19.42
**13m**	62.06	13.54	51.06	0.00	−21.69
**13n**	60.05	10.71	48.89	0.00	−17.88
**13o**	70.31	13.07	53.66	0.00	−16.55
**13p**	53.70	6.05	48.38	0.00	−18.86
**13q**	53.01	4.73	45.93	0.00	−14.87
Ibuprofen	41.19	5.84	27.89	0.00	−3.00

**Table 5:** ArgusLabs binding energy values of novel 1,2,3-triazoles (**13a**–**q**)

Compound	Argus B.E. (K cal/mol)	Elapsed time (s)	GA dock energy (K cal/mol)	Elapsed time (s)
**13a**	−13.2381	22	−12.0907	21
**13b**	−12.3289	86	−8.0643	21
**13c**	−15.5507	18	−11.4343	25
**13d**	−13.9083	19	−11.899	19
**13e**	−13.4295	26	−11.7591	17
**13f**	−13.2227	16	−1.36602	16
**13g**	−15.0759	14	−13.2652	21
**13h**	−12.3786	16	−14.1789	19
**13i**	−15.9765	17	−9.5211	19
**13j**	−14.9881	11	−15.6249	19
**13k**	−14.1346	14	−13.3685	19
**13l**	−14.1561	12	+3.0948	17
**13m**	−14.1131	15	−12.7818	18
**13n**	−14.9852	14	−12.7541	17
**13o**	−15.4749	10	−11.1716	20
**13p**	−12.1776	7	−12.5944	20
**13q**	−8.1178	7	−8.7334	19
Ibuprofen	−13.3519	6	−4.82611	8

Among several residues of the active site region of COX-2, TYR356 and ARG121 allows an important role of formation of hydrogen bonds, which favors interaction with COX-2 inhibitor. Molecular binding pattern of ibuprofen with COX-2 revealed that it has hydrogen bonds with ARG121 (bonding distances of 3.01, 2.97 Å) and TYR356 (bonding distances of 2.42, 5.21 Å) shown in Fig. 5. Similarly **13o** also formed hydrogen bonds with bond distances viz. 2.91, 2.95 Å for ARG121 and 4.10 Å for TYR356 shown in Fig. 6. It is also forming hydrogen bonds with VAL89, LEU360, LEU93, VAL117, VAL350, LEU353, LEU385, MET523, TYR386, TRP388, PHE519, ILE113, VAL524, ALA528 and GLY527. These interactions of **13o** in the COX-2 active site may be driving to have more activity. Compound **13o** has the highest fitness score 70.31 and good Chem score 35.66 with binding energy −15.4749 compared with the fitness score 41.19, Chem score 21.39 and binding energy −13.3519 of the standard drug ibuprofen (Tables 3, 4, 5). Compound **13l** established hydrogen bonds viz; 2.69, 4.53, 5.42 Å for ARG121 and 3.87, 4.27 Å for TYR356. It is also formed hydrogen bonds with LEU93, PHE358, VAL89, ILE113, TRP100, PHE358, VAL117, MET114 and TYR116 (Fig. 7). Compound **13l** showed fitness score 54.78 and Chem score 38.17 with binding energy −14.1561.

In this investigation, we discovered that the synthesized triazoles which showed good Chem score and Gold score functions, also exhibited good binding energy values. Finally, molecular docking studies showed good correlation between the in vivo anti-inflammatory activity of final compounds and their binding interactions with COX-2 as well as their Gold fitness scores. Among all compounds, **13o** and **13l** report good affinities with COX-2 (4PH9).

## Experimental section

### Chemistry

#### Synthesis and characterization data

Melting points were determined in open capillaries. ^1^H-Nuclear magnetic resonance (NMR) spectra were recorded on Bruker AV 300 and 400 MHz instruments, in CDCl_3_ using TMS as an internal standard. Chemical shifts are given in (δ) ppm and coupling constants (*J*) are given in Hz. Combinations of the following abbreviations are used to describe NMR spectra: s = singlet; d = doublet; t = triplet; q = quartet; m = multiplet. Thin layer chromatography (TLC) was carried out on aluminium sheets coated with silica gel 60 F_254_ (Merck, 1.05554) and the spots were visualized with UV light at 254 nm or alternatively by staining with aqueous basic potassium permanganate. Flash column chromatography was performed using silica gel (Merck, 60A, 100–200 mesh). Chromatographic purity by HPLC was determined by using area normalization method and the condition specified in each case: column, mobile phase, flow rate, detection wavelength, and retention times. Commercially available reagents were used as supplied and some of them were distilled before use. All reactions were performed in oven dried glassware. All solvents were removed by evaporation under reduced pressure.

#### General procedure for the synthesis of 1-(2,4-dihydroxyphenyl)-2-(4-isobutylphenyl)propan-1-one (**10**)

Ibuprofen (2 g, 9.69 mmol) was added to fused zinc chloride (1.98 g, 14.54 mmol) and heated to 120 °C for 20 min then resorcinol (1.06 g, 9.69 mmol) was added. The reaction mixture was heated to 140 °C for 30 min and monitored by TLC for complete conversion of starting materials. The reaction mixture was allowed to room temperature and was poured into ice cold water (100 mL), extracted with ethyl acetate (3 × 50 mL). The combined organic layers were washed with 5 % HCl (50 mL), saturated NaHCO_3_ (25 mL) and brine (2 × 25 mL). The organic layer was dried over anhydrous sodium sulphate, filtered and concentrated *in vacuo*. The crude product was purified by column chromatography using 100–200 mesh silica gel, eluted at 10 % ethyl acetate in pet ether to afford 1-(2,4-dihydroxy-phenyl)-2-phenyl-ethanone as light yellow liquid (2.2 g, 75 %).

Chemical formula: C_19_H_22_O_3_; ^1^H-NMR (400 MHz, CDCl_3_) δ 12.97 (s, 1H), 7.70 (d, *J* = 8.82 Hz, 1H), 7.22–7.16 (m, 2H), 7.12–7.06 (m, 2H), 6.33 (d, *J* = 2.30 Hz, 1H), 6.28 (dd, *J* = 8.78, 2.32 Hz, 1H), 4.60 (q, *J* = 6.79 Hz, 1H), 2.44–2.39 (m, 2H), 1.88–1.77 (m, 1H), 1.51 (d, *J* = 6.82 Hz, 3H), 0.88 (m, 6H); ^13^C-NMR (100 MHz, CDCl_3_) δ 205.3, 166.1, 163.2, 141.3, 139.3, 133.6, 130.5 (2C), 128.1 (2C), 114.0, 108.9, 104.6, 48.1, 46.6, 31.9, 24.2 (2C), 21.0; LRMS: (ES+) *m*/*z* = 299 [M + 1].

#### General procedure for the synthesis of 1-(2-hydroxy-4-(prop-2-yn-1-yloxy)phenyl)-2-(4-isobutylphenyl)propan-1-one (**11**)

Compound (**10**) (2.1 g, 7.038 mmol) was taken in dry acetone, anhydrous K_2_CO_3_ (0.972 g, 7.038 mmol) and propargyl bromide (0.837 g, 7.038, mmol) was added. This reaction mixture was refluxed for 8 h. Progress of the reaction was monitored by TLC, the reaction mixture was cooled to room temperature and solvent was removed *in vacuo*, then diluted with water (50 mL) and extracted with ethyl acetate (3 × 50 mL). The combined organic layers were washed with brine (2 × 25 mL). The organic layer was dried over anhydrous sodium sulphate, filtered and concentrated *in vacuo*. The crude product was purified by column chromatography using 100–200 mesh silica gel, eluted at 5 % ethyl acetate in pet ether to afford 1-(2-hydroxy-4-(prop-2-yn-1-yloxy)phenyl)-2-(4-isobutylphenyl)propan-1-one as light yellow liquid (2 g, 85 %).

Chemical formula: C_22_H_24_O_3_; ^1^H-NMR (400 MHz, CDCl_3_) δ 12.91 (s, 1H), 7.73 (d, *J* = 9.04 Hz, 1H), 7.20–7.17 (m, 2H), 7.08 (d, *J* = 8.00 Hz, 2H), 6.48 (d, *J* = 2.48 Hz, 1H), 6.40 (dd, *J* = 9.00, 2.51 Hz, 1H), 4.66 (s, 2H), 4.64–4.57 (m, 1H), 2.53 (t, *J* = 2.32 Hz, 1H), 2.41 (d, *J* = 7.18 Hz, 2H), 1.82 (m, 1H), 1.51 (d, *J* = 6.86 Hz, 3H), 0.87 (d, *J* = 6.60 Hz, 6H); ^13^C-NMR (75 MHz, CDCl_3_) δ 206.0, 166.7, 162.7, 141.6, 139.3, 133.6, 130.3 (2C), 128.7 (2C), 114.5, 110.1, 105.1, 78.8, 76.8, 57.0, 48.1, 46.9, 31.4, 24.2 (2C), 20.0; LRMS: (ES+) *m*/*z* = 337 [M + 1], 359 [M + Na].

#### General procedure for the synthesis of 1,4-disubstituted 1,2,3-triazole analogues (**13a**–**q**)

Propargyl derivative (**11**) (100 mg, 0.297 mmol) is dissolved in 5 mL aqueous *t*-BuOH (50 %) was added CuSO_4_. 5H_2_O (5 mol%) followed by sodium ascorbate (10 mol%) and azide (0.356 mmol) was added. The reaction mixture was stirred for 1 h at room temperature, monitored by TLC. After complete conversion of starting materials the reaction mixture was diluted with water (25 mL), extracted with ethyl acetate (3 × 25 mL). The combined organic layers were washed with brine (2 × 25 mL). The organic layer was dried over anhydrous sodium sulphate, filtered and concentrated *in vacuo*. The crude product was purified by column chromatography using 100–200 mesh silica gel and ethyl acetate in pet ether to afford corresponding 1,4-disubstituted 1,2,3-triazole analogues.

#### 1-(2-Hydroxy-4-((1-octyl-1*H*-1,2,3-triazol-4-yl)methoxy)phenyl)-2-(4-isobutylphenyl)propan-1-one (**13a**)

Chemical formula: C_30_H_41_N_3_O_3_; yield: 85 %; white solid; mp: 84–86 °C; ^1^H-NMR (300 MHz, CDCl_3_) δ 12.91 (s, 1H), 7.75 (d, *J* = 9.03 Hz, 1H), 7.59 (s, 1H), 7.21 (d, *J* = 8.04 Hz, 2H), 7.10 (d, *J* = 8.03 Hz, 2H), 6.52 (d, *J* = 2.44 Hz, 1H), 6.46 (dd, *J* = 8.95, 2.49 Hz, 1H), 5.22 (s, 2H), 4.64 (q, *J* = 6.85 Hz, 1H), 4.37 (t, *J* = 7.27 Hz, 2H), 2.45 (d, *J* = 7.15 Hz, 2H), 1.96–1.81 (m, 3H), 1.55 (d, *J* = 6.84 Hz, 3H), 1.32–1.27 (m, 9H), 0.94–0.9 (m, 10H); ^13^C-NMR (75 MHz, CDCl_3_) δ 205.1, 166.5, 165.0, 143.9, 141.5, 139.5, 133.2, 130.8 (2C), 128.3 (2C), 123.7, 114.5, 108.9, 103.6, 64.0, 52.5, 48.6, 47.1, 33.9, 32.5, 32.4, 31.3, 31.2, 28.8, 25.0, 24.8 (2C), 21.6, 16.5; LRMS: (ES+) *m*/*z* = 492 [M + 1], 514 [M + Na]; HPLC 98.03 %, column: phenomenex luna C8 (2) (250X4.6 mm), mobile phase: 90 % acetonitrile in 0.1 % formic acid, flow rate: 1.0 mL/min.

#### 1-(4-((1-Hexyl-1*H*-1,2,3-triazol-4-yl)methoxy)-2-hydroxyphenyl)-2-(4-isobutylphenyl)propan-1-one (**13b**)

Chemical formula: C_28_H_37_N_3_O_3_; yield: 90 %; white solid; mp: 83–85 °C; ^1^H-NMR (300 MHz, CDCl_3_) δ 12.90 (s, 1H), 7.72 (d, *J* = 8.97 Hz, 1H), 7.57 (s, 1H), 7.19 (d, *J* = 7.72 Hz, 2H), 7.08 (d, *J* = 7.68 Hz, 2H), 6.49 (s, 1H), 6.43 (d, *J* = 8.87 Hz, 1H), 5.19 (s, 2H), 4.61 (q, *J* = 6.74 Hz, 1H), 4.34 (t, *J* = 7.17 Hz, 2H), 2.41 (d, *J* = 7.07 Hz, 2H), 1.96–1.76 (m, 3H), 1.51 (d, *J* = 6.73 Hz, 3H), 1.26–1.35 (m, 6H), 0.91–0.84 (m, 9H); ^13^C-NMR (75 MHz, CDCl_3_) δ 205.2, 166.5, 164.9, 143.9, 141.5, 139.5, 133.3, 130.8 (2C), 128.3 (2C), 123.8, 114.5, 108.9, 103.6, 64.0, 52.5, 48.6, 47.1, 33.4, 32.5, 32.4, 28.5, 24.8 (3C), 21.6, 16.4; LRMS: (ES+) *m*/*z* = 464 [M + 1], 486 [M + Na]; HPLC 99.12 %, column: phenomenex luna C8 (2) (250X4.6 mm), mobile phase: 90 % acetonitrile in 0.1 % formic acid, flow rate: 1.0 mL/min.

#### 1-(4-((1-(4-Chlorophenyl)-1*H*-1,2,3-triazol-4-yl)methoxy)-2-hydroxyphenyl)-2-(4-isobutylphenyl)propan-1-one (**13c**)

Chemical formula: C_28_H_28_ClN_3_O_3_; yield: 81 %; white solid; mp: 79–81 °C; ^1^H-NMR (400 MHz, CDCl_3_) δ 12.92 (s, 1H), 8.01 (s, 1H), 7.74 (d, *J* = 9.07 Hz, 1H), 7.68 (d, *J* = 8.82 Hz, 2H), 7.50 (d, *J* = 8.85 Hz, 2H), 7.19 (d, *J* = 8.04 Hz, 2H), 7.08 (d, *J* = 8.04 Hz, 2H), 6.53 (d, *J* = 2.48 Hz, 1H), 6.45 (dd, *J* = 8.99, 2.51 Hz, 1H), 5.28 (s, 2H), 4.61 (q, *J* = 6.83 Hz, 1H), 2.41 (d, *J* = 7.17 Hz, 2H), 1.87–1.77 (m, 1H), 1.52 (d, *J* = 6.84 Hz, 3H), 0.87 (d, *J* = 6.53 Hz, 6H); ^13^C-NMR (126 MHz, CDCl_3_) δ 205.1, 166.2, 164.5, 144.7, 141.1, 139.1, 135.9, 135.4, 132.9, 130.6 (2C), 130.4 (2C), 127.9, 122.5 (2C), 121.7, 114.1, 108.3, 103.0, 63.1, 47.9, 46.3, 31.5, 23.8 (2C), 20.7; LRMS: (ES+) *m*/*z* = 490 [M + 1]; HPLC 96.19 %, column: X-BRIDGE C-18 (150X4.6 mm), mobile phase A: 0.1 % farmic acid in water, mobile phase B: acetonitrile, gradient (T/%B): 0/20, 3/20, 12/95, 23/95, 25/20, 30/20; flow rate: 1.0 mL/min.

#### 1-(4-((1-(Sec-butyl)-1*H*-1,2,3-triazol-4-yl)methoxy)-2-hydroxyphenyl)-2-(4-isobutylphenyl)propan-1-one (**13d**)

Chemical formula: C_26_H_33_N_3_O_3_; yield: 79 %; white solid; mp: 89–91 °C; ^1^H-NMR (400 MHz, CDCl_3_) δ 12.91 (s, 1H), 7.73 (d, *J* = 8.77 Hz, 1H), 7.61 (s, 1H), 7.19 (d, *J* = 7.85 Hz, 2H), 7.08 (d, *J* = 7.82 Hz, 2H), 6.50 (s, 1H), 6.45 (d, *J* = 8.68 Hz, 1H), 5.19 (s, 2H), 4.69–4.52 (m, 2H), 2.41 (d, *J* = 7.12 Hz, 2H), 1.98–1.77 (m, 3H), 1.57 (d, *J* = 6.41 Hz, 3H), 1.51 (d, *J* = 6.78 Hz, 3H), 0.91–0.83 (m, 9H); ^13^C-NMR (100 MHz, CDCl_3_) δ 205.0, 165.8, 164.4, 142.2, 140.5, 138.6, 132.2, 129.7 (2C), 127.2 (2C), 122.4, 113.2, 107.5, 102.2, 62.3, 59.1, 46.5, 45.0, 30.3, 30.1, 22.4 (2C), 20.8, 19.2, 10.4; LRMS: (ES+) *m*/*z* = 436 [M + 1], 458 [M + Na].

#### 1-(4-((1-Butyl-1*H*-1,2,3-triazol-4-yl)methoxy)-2-hydroxyphenyl)-2-(4-isobutylphenyl)propan-1-one (**13e**)

Chemical formula: C_26_H_33_N_3_O_3_; yield: 91 %; white solid; mp: 89–91 °C; ^1^H-NMR (400 MHz, CDCl_3_) δ 12.91 (s, 1H), 7.72 (d, *J* = 9.03 Hz, 1H), 7.58 (s, 1H), 7.19 (d, *J* = 7.94 Hz, 2H), 7.08 (d, *J* = 7.91 Hz, 2H), 6.49 (d, *J* = 2.24 Hz, 1H), 6.43 (dd, *J* = 8.98, 2.29 Hz, 1H), 5.18 (s, 2H), 4.61 (q, *J* = 6.74 Hz, 1H), 4.35 (t, *J* = 7.23 Hz, 2H), 2.41 (d, *J* = 7.14 Hz, 2H), 1.92–1.78 (m, 3H), 1.51 (d, *J* = 6.82 Hz, 3H), 1.40–1.30 (m, 2H), 0.95 (t, *J* = 7.35 Hz, 3H), 0.87 (d, *J* = 6.56 Hz, 6H); ^13^C-NMR (101 MHz, CDCl_3_) δ 205.0, 165.8, 164.3, 143.0, 140.5, 138.6, 132.2, 129.7 (2C), 127.2 (2C), 122.6, 113.2, 107.6, 102.2, 62.1, 50.2, 46.5, 45.0, 32.2, 30.1, 22.4 (2C) 19.7, 19.2, 13.4; LRMS: (ES+) *m*/*z* = 436 [M + 1], 458 [M + Na]; HPLC 99.31 %, column: phenomenex luna C8 (2) (250X4.6 mm), mobile phase: 90 % acetonitrile in 0.1 % formic acid, flow rate: 1.0 mL/min.

#### 1-(2-Hydroxy-4-((1-isobutyl-1*H*-1,2,3-triazol-4-yl)methoxy)phenyl)-2-(4-isobutylphenyl)propan-1-one (**13f**)

Chemical formula: C_26_H_33_N_3_O_3_; yield: 86 %; white solid; mp: 97–99 °C; ^1^H-NMR (400 MHz, CDCl_3_) δ 12.89 (s, 1H), 7.80–7.48 (m, 2H), 7.30–7.00 (m, 4H), 6.58–6.35 (m, 2H), 5.19 (d, *J* = 0.52 Hz, 2H), 4.72–4.54 (m, 1H), 4.25–4.08 (m, 2H), 2.52–2.11 (m, 3H), 1.81–1.51 (m, 5H), 1.09–0.73 (m, 12H); ^13^C-NMR (101 MHz, CDCl_3_) δ 205.0, 165.8, 164.3, 142.9, 140.5, 138.6, 132.2, 129.7 (2C), 127.2 (2C), 123.1, 113.2, 107.6, 102.2, 62.1, 57.6, 46.5, 45.0, 30.0, 29.6, 22.3 (2C), 19.8 (2C), 19.2; LRMS: (ES+) *m*/*z* = 436 [M + 1], 458 [M + Na]; HPLC 99.32 %, column: phenomenex luna C8 (2) (250X4.6 mm), mobile phase: 90 % acetonitrile in 0.1 % formic acid, flow rate: 1.0 mL/min.

#### 1-(2-Hydroxy-4-((1-phenyl-1*H*-1,2,3-triazol-4-yl)methoxy)phenyl)-2-(4-isobutylphenyl)propan-1-one (**13g**)

Chemical formula: C_28_H_29_N_3_O_3_; yield: 96 %; white solid; mp: 138–140 °C; ^1^H-NMR (500 MHz, CDCl_3_) δ 12.91 (s, 1H), 8.04 (s, 1H), 7.76–7.71 (m, 3H), 7.53 (t, *J* = 7.77 Hz, 2H), 7.45 (t, *J* = 7.40 Hz, 1H), 7.21 (d, *J* = 8.02 Hz, 2H), 7.09 (d, *J* = 8.00 Hz, 2H), 6.54 (d, *J* = 2.46 Hz, 1H),), 6.47 (dd, *J* = 8.99, 2.48 Hz, 1H), 5.29 (s, 2H), 4.63 (q, *J* = 6.81 Hz, 1H), 2.43 (d, *J* = 7.16 Hz, 2H), 1.87–1.79 (m, 1H), 1.54 (d, *J* = 6.84 Hz, 3H), 0.89 (d, *J* = 6.59 Hz, 6H); ^13^C-NMR (126 MHz, CDCl_3_) δ 205.1, 166.3, 164.6, 144.4, 141.1, 139.1, 137.4, 132.9, 130.4 (2C), 130.3 (2C), 129.6, 127.9 (2C), 121.8, 121.3 (2C), 114.1, 108.3, 103.1, 63.1, 47.8, 46.3, 31.5, 23.8 (2C), 20.7; LRMS: (ES+) *m*/*z* = 456 [M + 1], 478 [M + Na]; HPLC 99.28 %, column: X-BRIDGE C-18 (150X4.6 mm), mobile phase A: 0.1 % farmic acid in water, mobile phase B: acetonitrile, gradient (T/%B): 0/20, 3/20, 12/95, 23/95, 25/20, 30/20; flow rate: 1.0 mL/min.

#### 1-(2-Hydroxy-4-((1-(*p*-tolyl)-1*H*-1,2,3-triazol-4-yl)methoxy)phenyl)-2-(4-isobutylphenyl)propan-1-one (**13h**)

Chemical formula: C_29_H_31_N_3_O_3_; yield: 83 %; white solid; mp: 126–128 °C; ^1^H-NMR (400 MHz, CDCl_3_) δ 12.92 (s, 1H), 7.99 (s, 1H), 7.77–7.69 (m, 2H), 7.59 (d, *J* = 8.44 Hz, 1H), 7.31 (d, *J* = 8.15 Hz, 2H), 7.19 (d, *J* = 8.07 Hz, 2H), 7.08 (d, *J* = 8.06 Hz, 2H), 6.53 (d, *J* = 2.43 Hz, 1H), 6.46 (dd, *J* = 9.01, 2.50 Hz, 1H), 5.27 (s, 2H), 4.62 (q, *J* = 6.84 Hz, 1H), 2.43–2.39 (m, 5H), 1.85–1.77 (m, 1H), 1.51 (d, *J* = 6.85 Hz, 3H), 0.87 (d, *J* = 6.59 Hz, 6H); ^13^C-NMR (126 MHz, CDCl_3_) δ 205.1, 166.3, 164.6, 144.2, 141.1, 139.7, 139.2, 132.9, 130.9 (2C), 130.3 (2C), 127.9 (2C), 121.8, 121.3, 121.2 (2C), 114.1, 108.4, 103.1, 63.2, 47.8, 46.3, 31.5, 23.8 (2C), 22.5, 20.7; LRMS: (ES+) *m*/*z* = 470 [M + 1], 492 [M + Na]; HPLC 98.88 %, column: X-BRIDGE C-18 (150X4.6 mm), mobile phase A: 0.1 % farmic acid in water, mobile phase B: acetonitrile, gradient (T/%B): 0/20, 3/20, 12/95, 23/95, 25/20, 30/20; flow rate: 1.0 mL/min.

#### 1-(4-((1-(3-Chlorophenyl)-1*H*-1,2,3-triazol-4-yl)methoxy)-2-hydroxyphenyl)-2-(4-isobutylphenyl)propan-1-one (**13i**)

Chemical formula: C_28_H_28_ClN_3_O_3_; yield: 89 %; white solid; mp: 118–120 °C; ^1^H-NMR (400 MHz, CDCl_3_) δ 12.92 (s, 1H), 8.04 (s, 1H), 7.78 (t, *J* = 1.8 Hz, 1H), 7.74 (d, *J* = 9.1 Hz, 1H), 7.65–7.61 (m, 1H), 7.48–7.40 (m, 2H), 7.19 (d, *J* = 8.1 Hz, 2H), 7.08 (d, *J* = 8.0 Hz, 2H), 6.52 (d, *J* = 2.5 Hz, 1H), 6.45 (dd, *J* = 9.0, 2.5 Hz, 1H), 5.27 (s, 2H), 4.62 (q, *J* = 6.8 Hz, 1H), 2.41 (d, *J* = 7.1 Hz, 2H), 1.86–1.76 (m, 1H), 1.51 (d, *J* = 6.8 Hz, 3H), 0.87 (d, *J* = 6.6 Hz, 6H); ^13^C-NMR (126 MHz, CDCl_3_) δ 205.1, 166.2, 164.5, 144.7, 141.1, 139.1, 138.2, 136.2, 132.9, 131.5, 130.4 (2C), 129.7, 127.9 (2C), 121.8, 121.5, 119.2, 114.1, 108.3, 103.0, 63.0, 47.8, 46.2, 31.5, 23.8 (2C), 20.7; LRMS: (ES+) *m*/*z* = 490 [M + 1], 512 [M + Na]; HPLC 98.70 %, column: X-BRIDGE C-18 (150X4.6 mm), mobile phase A: 0.1 % farmic acid in water, mobile phase B: acetonitrile, gradient (T/%B): 0/20, 3/20, 12/95, 23/95, 25/20, 30/20; flow rate: 1.0 mL/min.

#### 1-(4-((1-Dodecyl-1*H*-1,2,3-triazol-4-yl)methoxy)-2-hydroxyphenyl)-2-(4-isobutylphenyl)propan-1-one (**13j**)

Chemical formula: C_34_H_49_N_3_O_3_; yield: 95 %; white solid; mp: 69–71 °C; ^1^H-NMR (400 MHz, CDCl_3_) δ 12.92 (s, 1H), 7.72 (d, *J* = 9.05 Hz, 1H), 7.58 (s, 1H), 7.19 (d, *J* = 7.92 Hz, 2H), 7.08 (d, *J* = 7.89 Hz, 2H), 6.49 (d, *J* = 2.18 Hz, 1H), 6.43 (dd, *J* = 8.98, 2.21 Hz, 1H), 5.18 (s, 2H), 4.61 (q, *J* = 6.72 Hz, 1H), 4.33 (t, *J* = 7.24 Hz, 2H), 2.41 (d, *J* = 7.14 Hz, 2H), 1.93–1.78 (m, 3H), 1.51 (d, *J* = 6.80 Hz, 3H), 1.33–1.23 (m, 19H), 0.90–0.85 (m, 9H); ^13^C-NMR (101 MHz, CDCl_3_) δ 205.0, 165.8, 164.3, 143.0, 140.5, 138.6, 132.2, 129.7 (2C), 127.2 (2C), 122.6, 113.2, 107.6, 102.2, 62.1, 50.5, 46.5, 45.0, 31.9, 30.2, 30.1, 29.6 (2C), 29.5, 29.3, 29.3, 28.9, 26.5, 22.7, 22.4, 22.4, 19.2, 14.1; LRMS: (ES+) *m*/*z* = 548 [M + 1], 570 [M + Na]; HPLC 98.21 %, column: phenomenex luna C8 (2) (250X4.6 mm), mobile phase: 90 % acetonitrile in 0.1 % formic acid, flow rate: 1.0 mL/min.

#### 1-(2-Hydroxy-4-((1-(3-nitrophenyl)-1*H*-1,2,3-triazol-4-yl)methoxy)phenyl)-2-(4-isobutylphenyl)propan-1-one (**13k**)

Chemical formula: C_28_H_28_N_4_O_5_; yield: 81 %; yellow solid; mp: 146–148 °C; ^1^H-NMR (400 MHz, CDCl_3_) δ 12.92 (s, 1H), 8.60 (s, 1H), 8.31 (d, *J* = 8.11 Hz, 1H), 8.18 (d, *J* = 6.46 Hz, 2H), 7.80–7.70 (m, 2H), 7.19 (d, *J* = 7.90 Hz, 2H), 7.08 (d, *J* = 7.88 Hz, 2H), 6.52 (d, *J* = 2.20 Hz, 1H), 6.46 (dd, *J* = 8.96, 2.24 Hz, 1H), 5.30 (s, 2H), 4.62 (q, *J* = 6.67 Hz, 1H), 2.41 (d, *J* = 7.13 Hz, 2H), 1.87–1.76 (m, 1H), 1.51 (d, *J* = 6.79 Hz, 3H), 0.87 (d, *J* = 6.55 Hz, 6H); ^13^C-NMR (101 MHz, CDCl_3_) δ 205.1, 165.8, 164.0, 148.9, 144.7, 140.6, 138.5, 137.5, 132.3, 131.0, 129.7 (2C), 127.2 (2C), 126.0, 123.4, 121.1, 115.3, 113.4, 107.5, 102.2, 61.8, 46.6, 45.0, 30.1, 22.3 (2C), 19.2; LRMS: (ES+) *m*/*z* = 501 [M + 1], 523 [M + Na]; HPLC 99.69 %, column: phenomenex luna C8 (2) (250X4.6 mm), mobile phase: 90 % acetonitrile in 0.1 % formic acid, flow rate: 1.0 mL/min.

#### 1-(2-Hydroxy-4-((1-(4-nitrophenyl)-1*H*-1,2,3-triazol-4-yl)methoxy)phenyl)-2-(4-isobutylphenyl)propan-1-one (**13l**)

Chemical formula: C_28_H_28_N_4_O_5_; yield: 77 %; yellow solid; mp: 167–169 °C; ^1^H-NMR (400 MHz, CDCl_3_) δ 12.93 (s, 1H), 8.42 (d, *J* = 9.0 Hz, 2H), 8.19 (s, 1H), 7.99 (d, *J* = 9.0 Hz, 2H), 7.77 (d, *J* = 9.0 Hz, 1H), 7.21 (d, *J* = 8.0 Hz, 2H), 7.10 (d, *J* = 8.0 Hz, 2H), 6.53 (d, *J* = 2.4 Hz, 1H), 6.46 (dd, *J* = 8.9, 2.4 Hz, 1H), 5.31 (s, 2H), 4.63 (q, *J* = 6.8 Hz, 1H), 2.43 (d, *J* = 7.1 Hz, 2H), 1.88–1.78 (m, 1H), 1.53 (d, *J* = 6.8 Hz, 3H), 0.89 (d, *J* = 6.6 Hz, 6H); ^13^C-NMR (101 MHz, CDCl_3_) δ 205.1, 165.8, 163.9, 147.3, 144.9, 140.9, 140.6, 138.5, 132.3, 129.7 (2C), 127.2 (2C), 125.5 (2C), 120.9, 120.6 (2C), 113.5, 107.4, 102.2, 61.8, 46.6, 45.0, 30.1, 22.4, 22.3 (2C), 19.2; LRMS: (ES+) *m*/*z* = 501 [M + 1]; HPLC 99.87 %, column: phenomenex luna C8 (2) (250X4.6 mm), mobile phase: 90 % acetonitrile in 0.1 % formic acid, flow rate: 1.0 mL/min.

#### 1-(4-((1-Cyclohexyl-1*H*-1,2,3-triazol-4-yl)methoxy)-2-hydroxyphenyl)-2-(4-isobutylphenyl)propan-1-one (**13m**)

Chemical formula: C_28_H_35_N_3_O_3_; yield: 89 %; white solid; mp: 118–120 °C; ^1^H-NMR (400 MHz, CDCl_3_) δ 12.92 (s, 1H), 7.73 (d, *J* = 9.10 Hz, 1H), 7.60 (s, 1H), 7.19 (d, *J* = 8.08 Hz, 2H), 7.08 (d, *J* = 8.08 Hz, 2H), 6.50 (d, *J* = 2.50 Hz, 1H), 6.44 (dd, *J* = 9.00, 2.53 Hz, 1H), 5.18 (s, 2H), 4.61 (q, *J* = 6.86 Hz, 1H), 4.45 (tt, *J* = 11.85, 3.85 Hz, 1H), 2.41 (d, *J* = 7.18 Hz, 2H), 2.25–2.17 (m, 2H), 1.97–1.88 (m, 2H), 1.77 (m, 3H), 1.51 (d, *J* = 6.87 Hz, 3H), 1.49–1.39 (m, 2H), 1.33–1.23 (m, 2H), 0.87 (d, *J* = 6.61 Hz, 6H); ^13^C-NMR (126 MHz, CDCl_3_) δ 205.1, 166.2, 164.8, 143.1, 141.1, 139.2, 132.8, 130.3 (2C), 127.9 (2C), 121.3, 114.0, 108.4, 103.0, 63.3, 61.4, 47.8, 46.3, 34.9 (2C), 31.5, 26.5 (2C), 26.5, 23.8 (2C), 20.7; LRMS: (ES+) *m*/*z* = 462 [M + 1]; HPLC 99.89 %, column: phenomenex luna C8 (2) (250X4.6 mm), mobile phase: 90 % acetonitrile in 0.1 % formic acid, flow rate: 1.0 mL/min.

#### 1-(4-((1-Cyclopentyl-1*H*-1,2,3-triazol-4-yl)methoxy)-2-hydroxyphenyl)-2-(4-isobutylphenyl)propan-1-one (**13n**)

Chemical formula: C_27_H_33_N_3_O_3_; yield: 82 %; white solid; mp: 130–132 °C; ^1^H-NMR (400 MHz, CDCl_3_) δ 12.92 (s, 1H), 7.73 (d, *J* = 8.7 Hz, 1H), 7.60 (s, 1H), 7.19 (d, *J* = 8.0 Hz, 2H), 7.08 (d, *J* = 8.0 Hz, 2H), 6.50 (d, *J* = 2.5 Hz, 1H), 6.44 (dd, *J* = 8.6, 2.5 Hz, 1H), 5.17 (s, 2H), 4.96–4.88 (m, 1H), 4.61 (q, *J* = 6.84 Hz, 1H), 2.41 (d, *J* = 7.2 Hz, 2H), 2.31–2.21 (m, 2H), 2.09–1.99 (m, 2H), 1.96–1.86 (m, 2H), 1.84–1.74 (m, 3H), 1.51 (d, *J* = 6.8 Hz, 3H), 0.87 (d, *J* = 6.5 Hz, 6H); LRMS: (ES+) *m*/*z* = 448 [M + 1], 470 [M + Na]; HPLC 99.37 %, column: phenomenex luna C8 (2) (250X4.6 mm), mobile phase: 90 % acetonitrile in 0.1 % formic acid, flow rate: 1.0 mL/min.

#### 1-(2-Hydroxy-4-((1-(4-nitrobenzyl)-1H-1,2,3-triazol-4-yl)methoxy)phenyl)-2-(4-isobutylphenyl)propan-1-one (**13o**)

Chemical formula: C_29_H_30_N_4_O_5_; yield: 90 %; white solid; mp: 150–152 °C; ^1^H-NMR (400 MHz, CDCl_3_) δ 12.91 (s, 1H), 8.24 (d, *J* = 8.4 Hz, 2H), 7.73 (d, *J* = 9.07 Hz, 1H), 7.61 (s, *J* = 3.97 Hz, 1H), 7.45–7.39 (m, 2H), 7.28 (s, 1H), 7.19 (d, *J* = 7.30 Hz, 2H), 7.10 (d, 1H), 6.49 (d, *J* = 2.49 Hz, 1H), 6.42 (dd, *J* = 8.98, 2.53 Hz, 1H), 5.66 (s, *J* = 3.05 Hz, 2H), 5.21 (s, *J* = 3.99 Hz, 2H), 4.63 (q, *J* = 6.82 Hz, 1H), 2.44 (d, *J* = 6.00 Hz, 2H), 1.89–1.80 (m, 1H), 1.53 (d, *J* = 8.49 Hz, 3H), 0.90 (d, *J* = 6.42 Hz, 6H); ^13^C-NMR (100 MHz, CDCl_3_)δ 205.18, 166.37, 164.60, 148.83, 144.88, 142.04, 141.33, 139.30, 133.07, 130.5 (2C), 129.5 (2C), 128.0 (2C), 125.2 (2C), 123.88, 114.35, 108.58, 103.24, 63.43, 54.77, 48.21, 46.61, 31.88, 24.21 (2C), 21.06; LRMS: (ES+) *m*/*z* = 515 [M + 1], 537 [M + Na]; HPLC 99.68 %, column: phenomenex luna C8 (2) (250X4.6 mm), mobile phase: 90 % acetonitrile in 0.1 % formic acid, flow rate: 1.0 mL/min.

#### 1-(4-((1-(4-Bromophenyl)-1*H*-1,2,3-triazol-4-yl)methoxy)-2-hydroxyphenyl)-2-(4-isobutylphenyl)propan-1-one (**13p**)

Chemical formula: C_28_H_28_BrN_3_O_3_; yield: 86 %; white solid; mp: 133–135 °C; ^1^H-NMR (400 MHz, CDCl_3_) δ 12.92 (s, 1H), 8.02 (s, 1H), 7.74 (d, *J* = 9.07 Hz, 1H), 7.63 (m, *J* = 8.98, 2.18 Hz, 4H), 7.19 (d, *J* = 8.02 Hz, 2H), 7.08 (d, *J* = 8.00 Hz, 2H), 6.52 (d, *J* = 2.44 Hz, 1H), 6.45 (dd, *J* = 8.99, 2.47 Hz, 1H), 5.27 (s, 2H), 4.61 (q, *J* = 6.81 Hz, 1H), 2.41 (d, *J* = 7.16 Hz, 2H), 1.82–1.79 (m, 1H), 1.51 (d, *J* = 6.84 Hz, 3H), 0.87 (d, *J* = 6.59 Hz, 6H); ^13^C-NMR (101 MHz, CDCl_3_) δ 205.2, 166.3, 164.6, 144.9, 141.3, 139.3, 136.5, 133.7 (2C), 133.1, 130.5 (2C), 128.1 (2C), 123.6, 122.9 (2C), 121.8, 114.3, 108.5, 103.3, 63.3, 48.2, 46.6, 31.9, 24.2 (2C), 21.0; LRMS: (ES+) *m*/*z* = 535 [M + 1].

#### 1-(4-((1-(2-Chlorophenyl)-1*H*-1,2,3-triazol-4-yl)methoxy)-2-hydroxyphenyl)-2-(4-isobutylphenyl)propan-1-one (**13q**)

Chemical formula: C_28_H_28_ClN_3_O_3_; yield: 83 %; white solid; mp: 80–82 °C; ^1^H-NMR (400 MHz, CDCl_3_) δ 12.91 (s, 1H), 8.06 (s, 1H), 7.76 (d, *J* = 8.99 Hz, 1H), 7.67–7.58 (m, 2H), 7.52–7.46 (m, 2H), 7.21 (d, *J* = 7.77 Hz, 2H), 7.10 (d, *J* = 7.74 Hz, 2H), 6.56 (d, *J* = 1.86 Hz, 1H), 6.53–6.46 (m, 1H), 5.32 (s, 2H), 4.64 (q, *J* = 6.73 Hz, 1H), 2.44 (d, *J* = 7.04 Hz, 2H), 1.88–1.81 (m, 1H), 1.55 (d, *J* = 6.72 Hz, 3H), 0.90 (d, *J* = 6.46 Hz, 6H); ^13^C-NMR (101 MHz, CDCl_3_) δ 205.2, 166.4, 164.7, 143.6, 141.2, 139.3, 135.5, 133.0, 131.7, 131.6, 130.5 (2C), 129.4, 128.8, 128.6, 128.1(2C), 125.9, 114.3, 108.6, 103.3, 63.4, 48.2, 46.6, 31.8, 24.2 (2C), 21.0; LRMS: (ES+) *m*/*z* = 490 [M + 1], 512 [M + Na]; HPLC 99.35 %, column: phenomenex luna C8 (2) (250X4.6 mm), mobile phase: 90 % acetonitrile in 0.1 % formic acid, flow rate: 1.0 mL/min.

### Biological evaluation

#### Animals

Wistar strain albino rats of male sex weighing 150–250 g were purchased from National Institute of Nutrition, Hyderabad, India and housed under standard environmental conditions (temperature: 24 ± 1 °C, light/dark cycle: 10/14 h). The rats were fed with standard pellet diet (Amrut laboratory animal feed, Maharashtra, India) and water ad libitum. Animals were acclimatized to laboratory conditions at least 1 week before conducting the experiments according to the guide lines of CPCSEA–New Delhi (Registration No.—915/ac/05/CPCSEA).

#### In vivo anti-inflammatory activity

The synthesized compounds assessed for their anti-inflammatory activity using carrageenan-induced rat paw edema method. Male Wistar rats (150–250 g) were fasted with access of water at least 24 h prior to the experiments and were divided randomly into different groups (control, standard and the test groups) of five rats each. The rat paw edema was induced by subcutaneous injection of 0.1 mL of 1 % freshly prepared saline solution of carrageenan into the right hind paw of rats. The standard drug ibuprofen (10 mg/kg body weight) given orally as a positive control. The control group was administered orally with 0.9 % of 0.1 mL of saline solution only. The test groups were administered orally with the synthesized compounds at the equimolar dosage of the standard drug, 1 h before the administration of carrageenan. The paw volumes were measured using plethysmometer at interval of 1 h.

### Bactericidal assay

#### Bacterial strains

Gram positive strains methicillin-resistant *S. aureus* (MRSA, NCTC 13616), *Bacillus subtilis* (ATCC 6633), *Bacillus cereus* (ATCC 14579) and gram negative strains *Klebsiella pneumoniae* (ATCC 43816), *Escherichia coli* (ATCC 8739), *Proteus vulgaris* (ATCC 13315) were procured from American type culture collection, USA. Methicillin-resistant *S. aureus* was purchased from culture collections, UK. All bacterial strains stored at −80 °C were streaked on Luria–Bertani (LB) agar plates (Hi-media Laboratories, Mumbai, India) and incubated at 37 °C for 20 to 24 h. A few isolated colonies were selected from each plate and suspended in 5 mL of LB broth in sterile culture vessel. The vessel was plugged with cotton and incubated with gentle shaking (140 rpm) at 37 °C for 20 h.

#### Determination of bactericidal activity

The assay was conducted to assess the bactericidal activities of synthesized compounds through microtiter plates (Lambert et al. 2001). The assay reaction mixture consisted of phosphate-buffered saline (PBS) [50 mM sodium phosphate, 150 mM NaCl (pH 7.0)], the test compound at various concentrations and the bacterial strains were prepared in sterile 96-well microtiter plates (Nunc, Inc). The wells are filled with 100 µL diluted test compounds in PBS and 50 µL of the diluted bacterial strains. The wells were incubated with gentle shaking (140 rpm) at 37 °C for various incubation periods 0 (baseline), 2, 4, 8, 12 and 24 h (time-kill studies). For positive and negative controls, a separate microtiter plate was prepared and screened for each incubation time studied (0, 2, 4, 8, 12 and 24 h). Following incubation, a 20-µL aliquot from each well was spotted at the top of a square plate containing nutrient agar medium. The plate was labelled and tapped gently to facilitate the movement of the liquid. There were approximately 200 cells in the spotted (20-µL) sample. Plates were placed uncovered in biohood until the sample liquid dried (ca. 10 min) and incubated overnight at 37 °C. The colony forming units (CFU) for each streak were enumerated after 24 h using a colony counter.

The number of CFU at each dilution of test compounds was compared with the average of positive control value to determine the percentage of bacteria killed per well. The percentage of the bacteria killed was plotted graphically, and the percentage of the test compound resulting decrease in the number of CFU (MIC/MBC) was determined.

### Molecular modelling approach

In the present study, Hyperchem 8.0, Swiss Protein Data Base Viewer (SPDBV) 3.7 (Johansson et al. 2012) version, GOLD Version 2.0, ArgusLabs 4.0.1 and Discovery studio visualiser 4.1 docking programs were evaluated to determine the interactions, affinities, binding energies and selectivity’s of compounds (**13a**–**q**). Ligands energy minimization was carried out by using Hyperchem. 8.0 version. The protein–ligand interactions between COX-2 (PDB code 4PH9) and target molecules (**13a**–**q**) were prepared for docking studies by adding hydrogen atoms, removing water molecules, co-crystallized inhibitors and refined by using the Deep View/SPDBV. Basic amines were protonated and acidic carboxyl groups were de-protonated prior to charge calculation. Then successful docking has been performed using GOLD 2.0. GOLD was used to evaluate Chem score and Gold fitness functions. ArgusLab 4.0.1 docking software used here to visualize the binding conformations and to calculate the binding energies of the analogues (**13a**–**q**). Discovery studio visualizer has been utilized to visualize the best binding poses of the final target analogues (**13a**–**q**) within the active site of 4PH9 protein.

## Conclusions

We have designed and synthesized a number of hybrid molecules containing ibuprofen-resorcinol-triazole moieties in single molecule using Click chemistry. These synthesized analogues (**13a**–**q**) were screened for in vivo anti-inflammatory. Compounds **13l**, **13g**, **13c**, **13k**, **13i**, **13n**, **13m** and **13j** were shown significant activity. Most of the biological experimental values correlated with docking results. These molecular binding interactions of an in silico data demonstrated that **13o** has more specificity towards the COX-2 binding site and could be a potent anti-inflammatory compound. These final derivatives were also evaluated for bactericidal activity. Compounds **13c**, **13i**, **13l** and **13o** exhibited good bactericidal profile. Finally, among all compounds **13o**, **13c**, **13i** and **13l** showed an interesting dual anti-inflammatory and antibacterial activity. These results gave us positive encouragement to develop further novel chemical entities towards challenging biological agents.

## Additional file


10.1186/s40064-016-2052-5 Supplementary material (Copies of ^1^H-NMR, ^13^C-NMR, LRMS and HPLC) for synthesized compounds.
