# Supplementary material for: Synthesis, anti-inflammatory, bactericidal activities and docking studies of novel 1,2,3-triazoles derived from ibuprofen using click chemistry
Source: Springerplus. 2016 Apr 11;5:423. doi: 10.1186/s40064-016-2052-5 (PMC4828371; doi:10.1186/s40064-016-2052-5)
Supplement: Supplementary file 1 — 10.1186/s40064-016-2052-5 Supplementary material (Copies of 1H-NMR, 13C-NMR, LRMS and HPLC) for synthesized compounds. [file 40064_2016_2052_MOESM1_ESM.docx]

**Additional file 1**

**Supplementary material**. Supplementary material (Copies of ^1^H-NMR, ^13^C-NMR, LRMS and HPLC) for synthesized compounds.

^1^H-NMR of compound 1-(2,4-dihydroxyphenyl)-2-(4-isobutylphenyl)propan-1-one

(**10**).

^
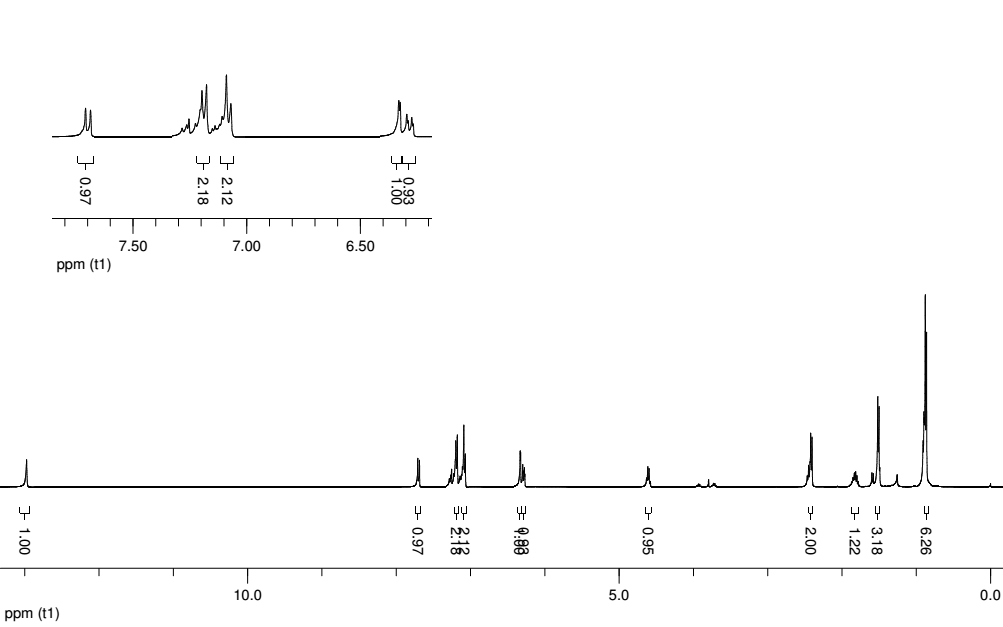
^

^13^C- NMR of compound 1-(2,4-dihydroxyphenyl)-2-(4-isobutylphenyl)propan-1-one

(**10**).

^
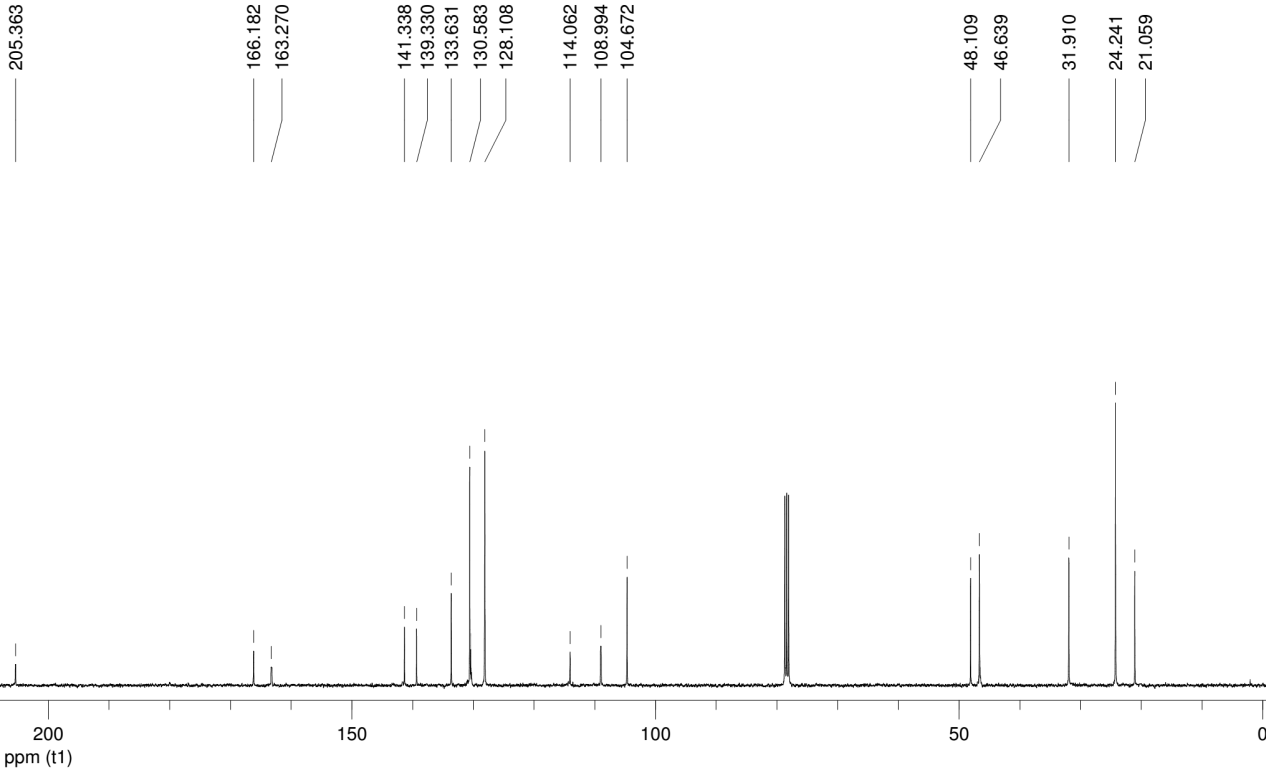
^

^1^H-NMR of compound 1-(2-hydroxy-4-(prop-2-yn-1-yloxy)phenyl)-2-(4-isobutylphenyl)propan-1-one (**11**).

**
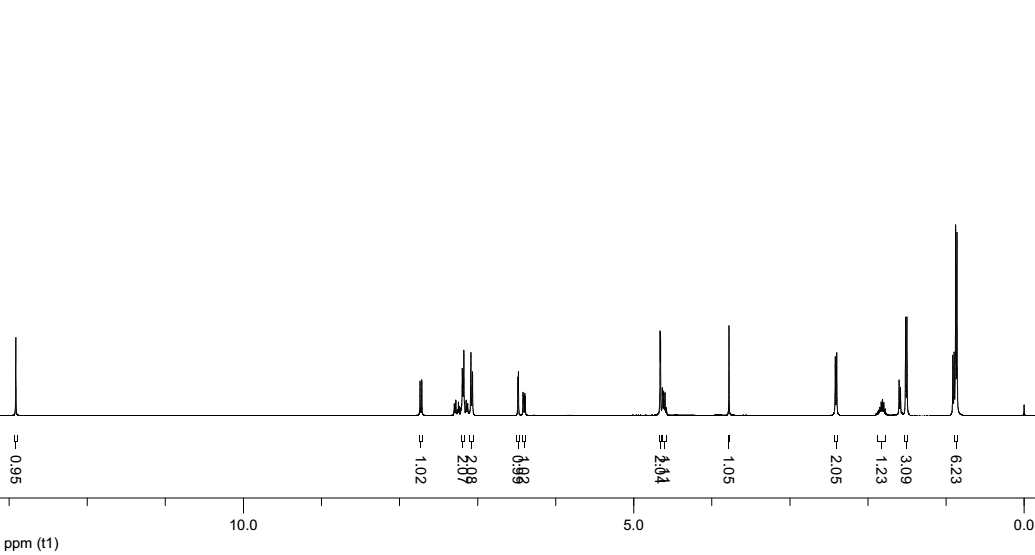
**

^13^C- NMR of compound 1-(2-hydroxy-4-(prop-2-yn-1-yloxy)phenyl)-2-(4-isobutylphenyl)propan-1-one (**11**).

**
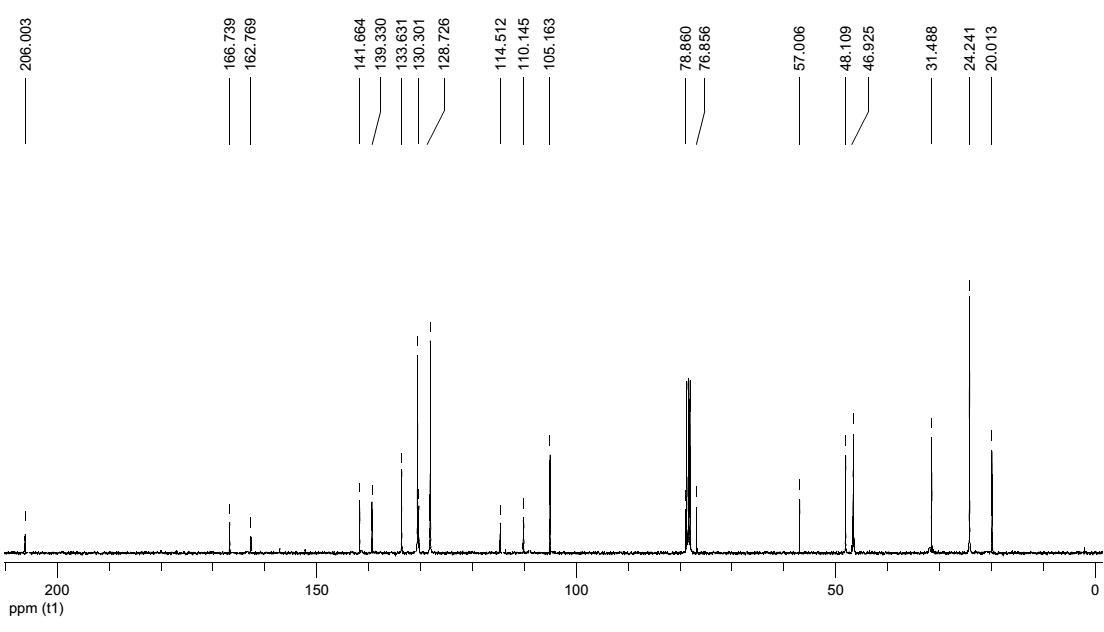
**

^1^H-NMR of compound 1-(2-hydroxy-4-((1-octyl-1H-1,2,3-triazol-4-yl)methoxy)phenyl)-2-(4-isobutylphenyl)propan-1-one (**13a**).


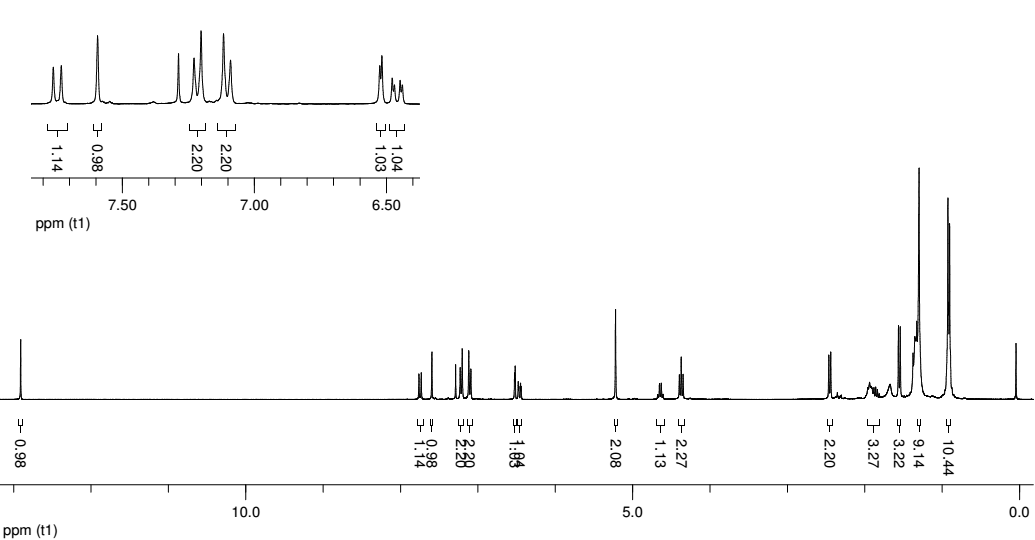


^13^C- NMR of compound 1-(2-hydroxy-4-((1-octyl-1H-1,2,3-triazol-4-yl)methoxy)phenyl)-2-(4-isobutylphenyl)propan-1-one (**13a**).


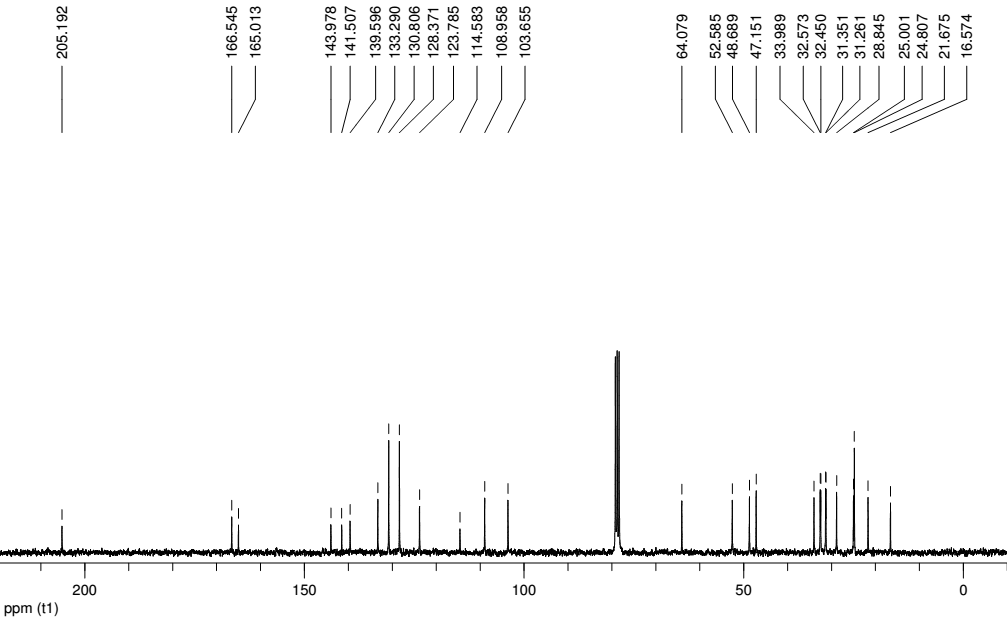


^1^H-NMR of compound 1-(4-((1-hexyl-1H-1,2,3-triazol-4-yl)methoxy)-2-hydroxyphenyl)-2-(4-isobutylphenyl)propan-1-one (**13b**).


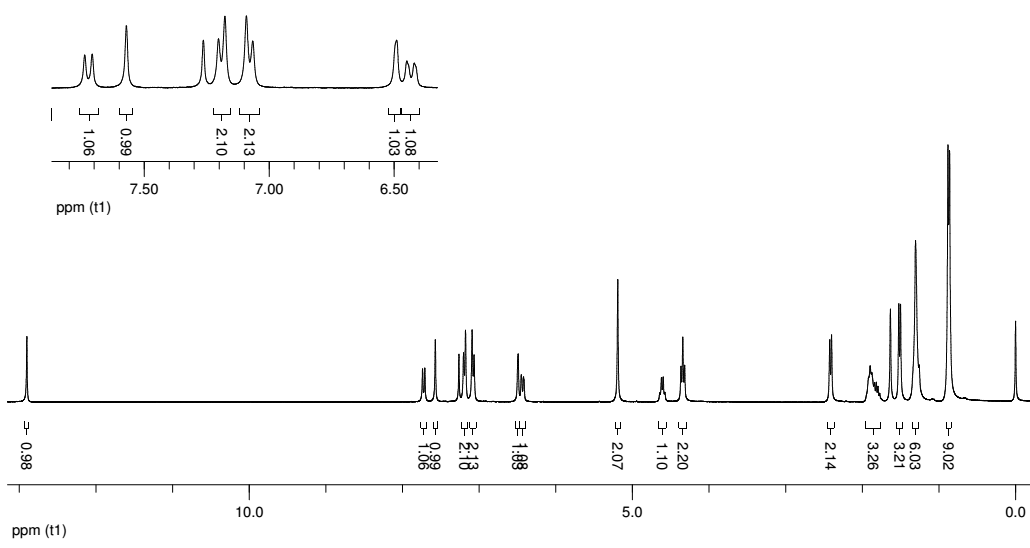


^13^C- NMR of compound 1-(4-((1-hexyl-1H-1,2,3-triazol-4-yl)methoxy)-2-hydroxyphenyl)-2-(4-isobutylphenyl)propan-1-one (**13b**).


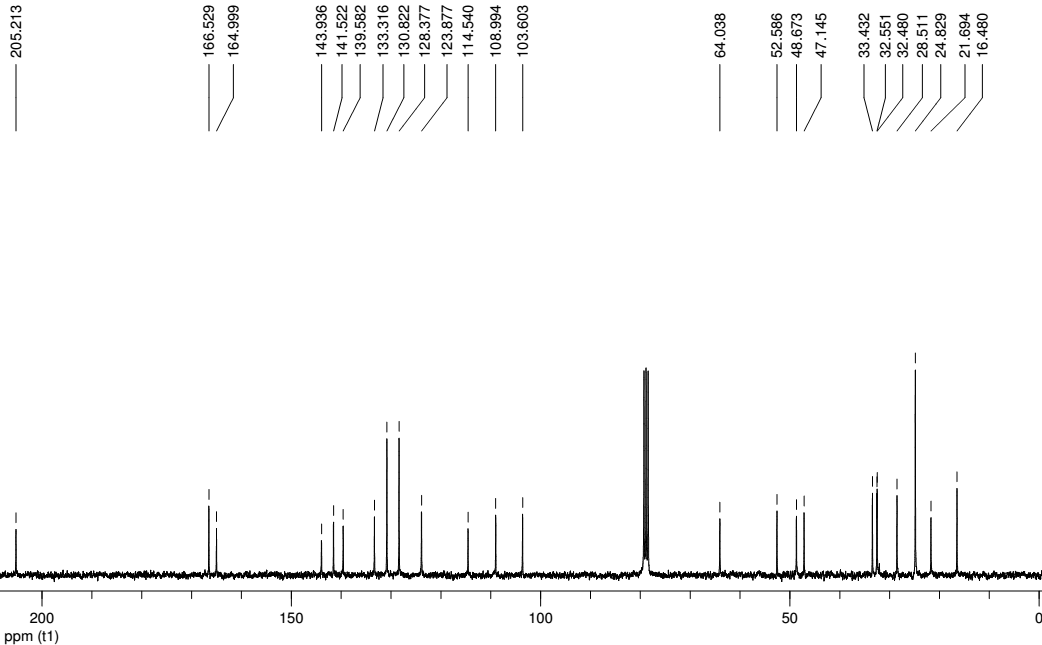


^1^H-NMR of compound 1-(4-((1-(4-chlorophenyl)-1H-1,2,3-triazol-4-yl)methoxy)-2-hydroxyphenyl)-2-(4-isobutylphenyl)propan-1-one (**13c**).


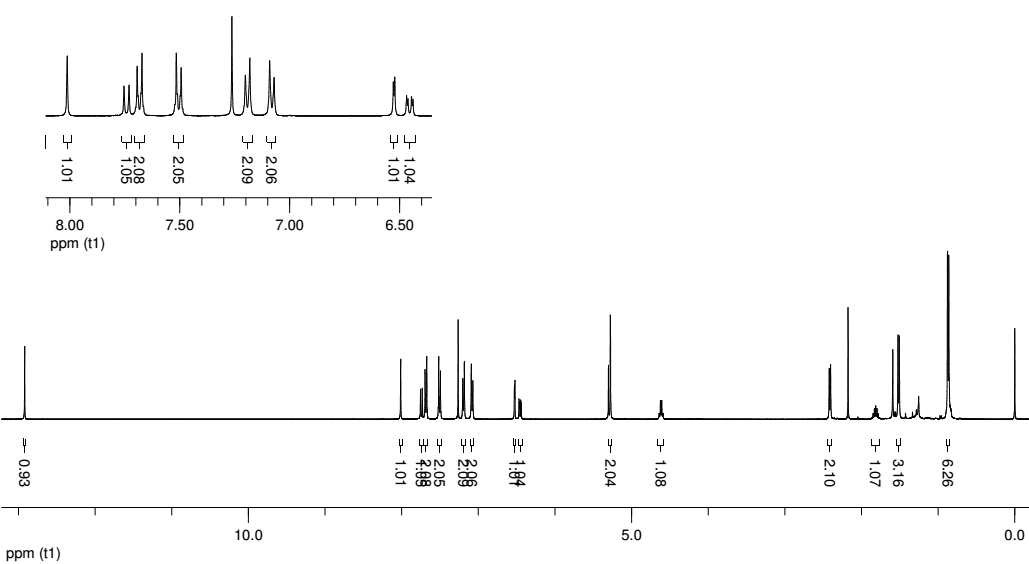


^13^C- NMR of compound 1-(4-((1-(4-chlorophenyl)-1H-1,2,3-triazol-4-yl)methoxy)-2-hydroxyphenyl)-2-(4-isobutylphenyl)propan-1-one (**13c**).


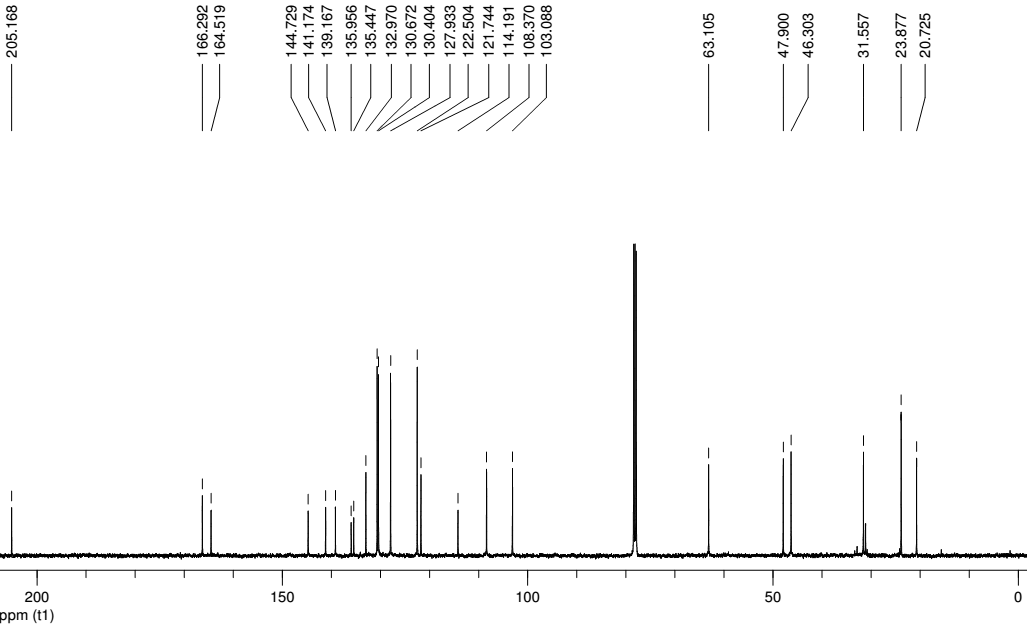


^1^H-NMR of compound 1-(4-((1-(sec-butyl)-1H-1,2,3-triazol-4-yl)methoxy)-2-hydroxyphenyl)-2-(4-isobutylphenyl)propan-1-one (**13d**).


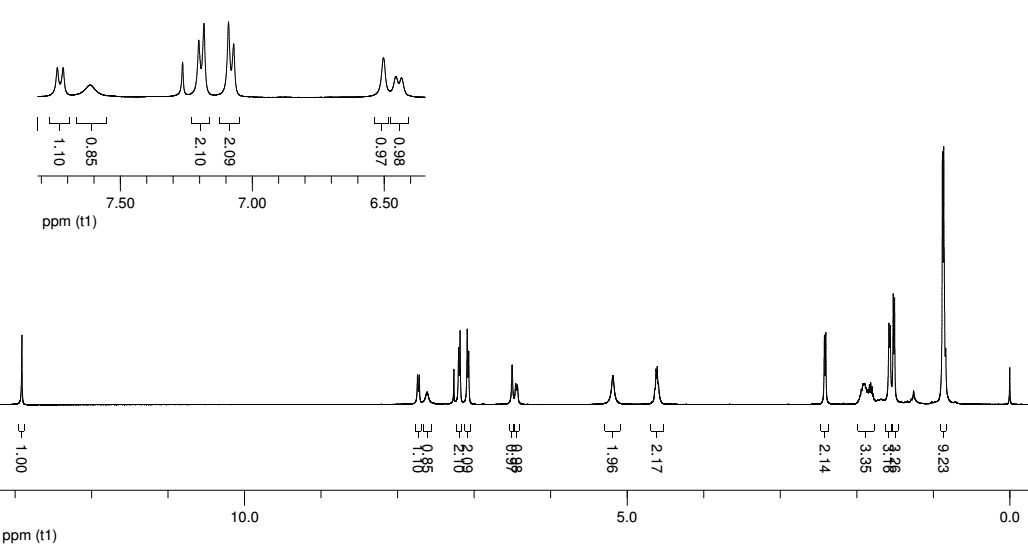


^13^C- NMR of compound 1-(4-((1-(sec-butyl)-1H-1,2,3-triazol-4-yl)methoxy)-2-hydroxyphenyl)-2-(4-isobutylphenyl)propan-1-one (**13d**).


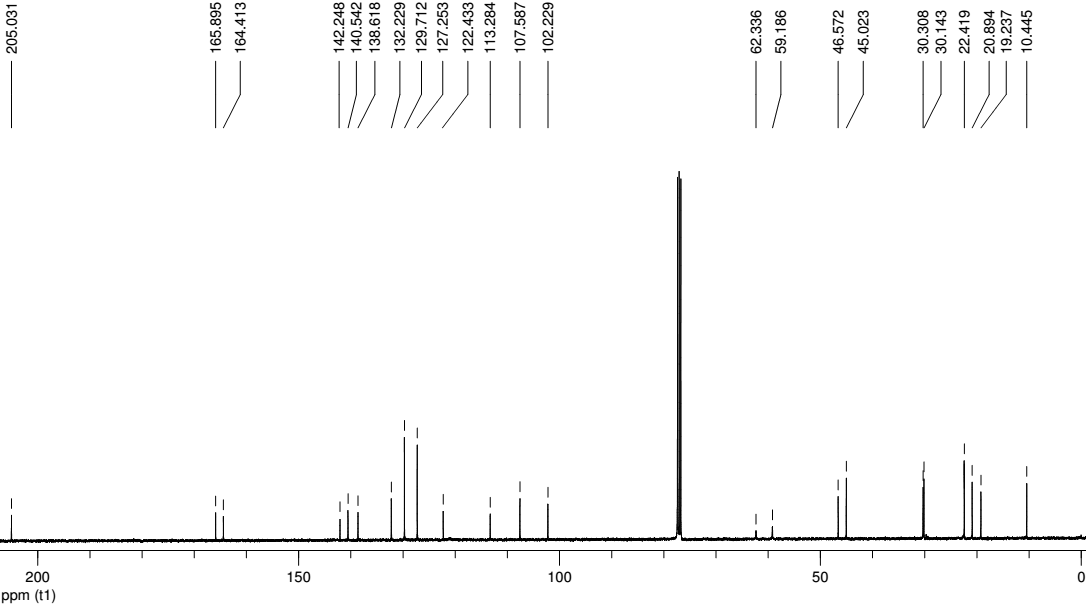


^1^H-NMR of compound 1-(4-((1-butyl-1H-1,2,3-triazol-4-yl)methoxy)-2-hydroxyphenyl)-2-(4-isobutylphenyl)propan-1-one (**13e**).


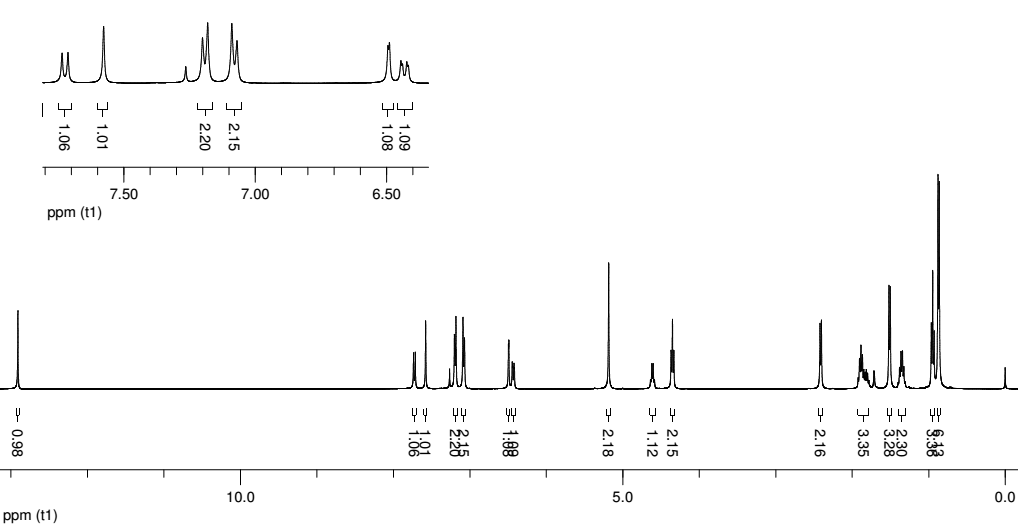


^13^C- NMR of compound 1-(4-((1-butyl-1H-1,2,3-triazol-4-yl)methoxy)-2-hydroxyphenyl)-2-(4-isobutylphenyl)propan-1-one (**13e**).


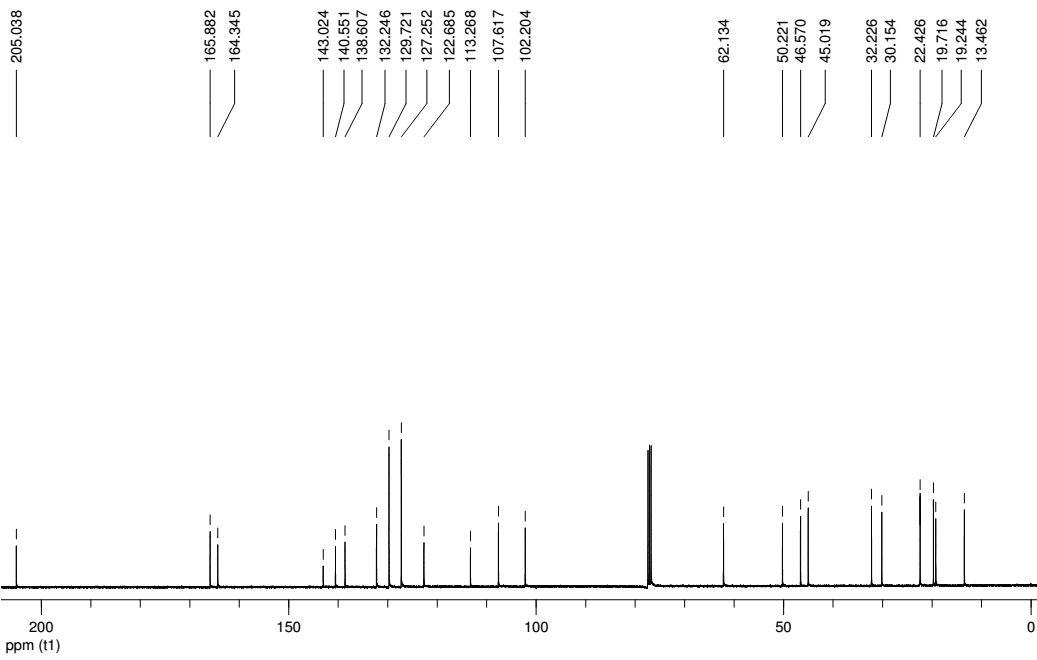


^1^H-NMR of compound 1-(2-hydroxy-4-((1-isobutyl-1H-1,2,3-triazol-4-yl)methoxy)phenyl)-2-(4-isobutylphenyl)propan-1-one (**13f**).


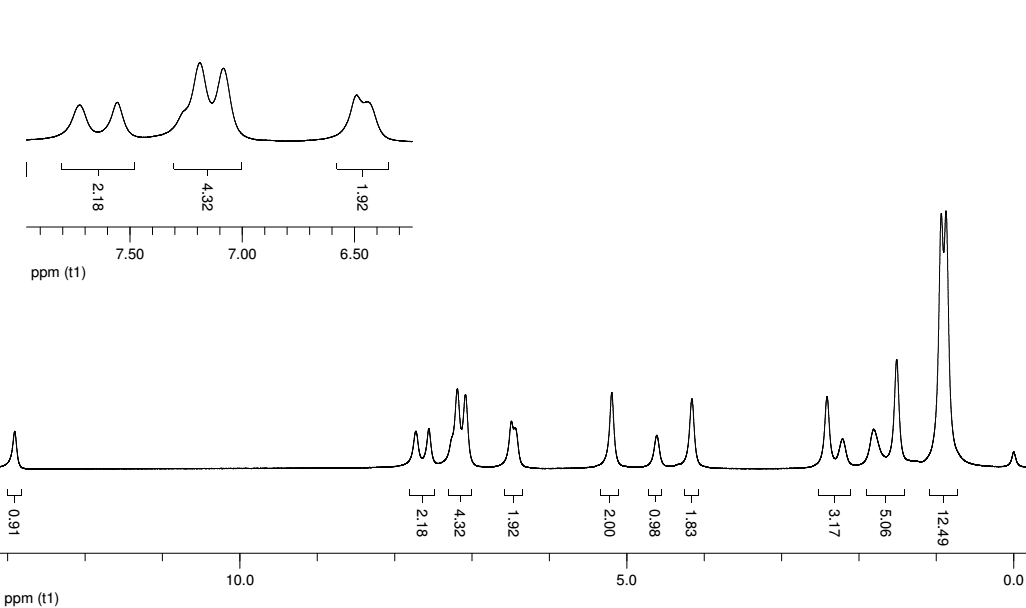


^13^C- NMR of compound 1-(2-hydroxy-4-((1-isobutyl-1H-1,2,3-triazol-4-yl)methoxy)phenyl)-2-(4-isobutylphenyl)propan-1-one (**13f**).


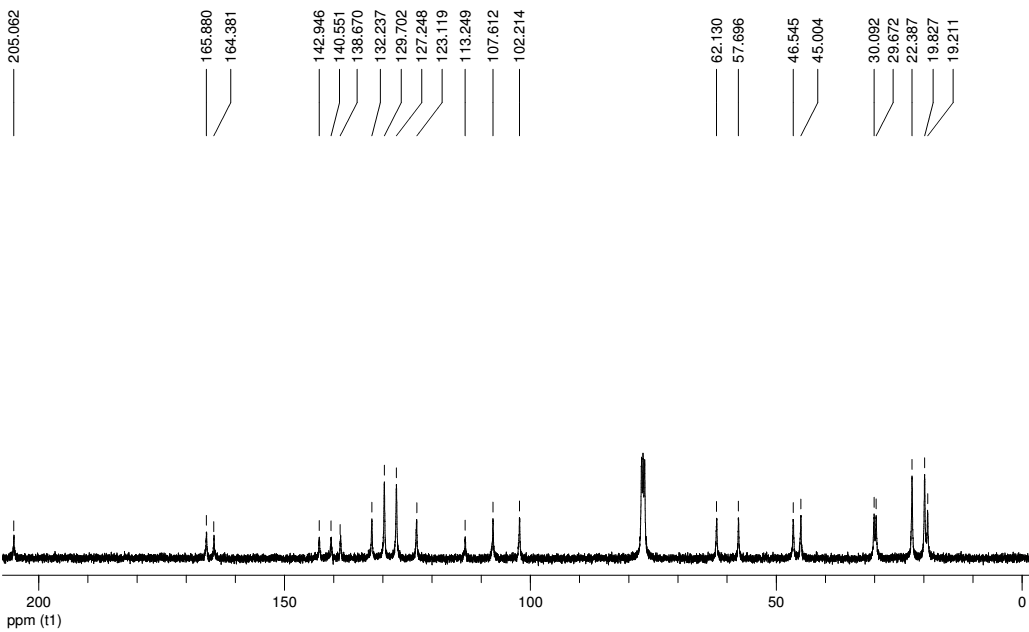


^1^H-NMR of compound 1-(2-hydroxy-4-((1-phenyl-1H-1,2,3-triazol-4-yl)methoxy)phenyl)-2-(4-isobutylphenyl)propan-1-one (**13g**).


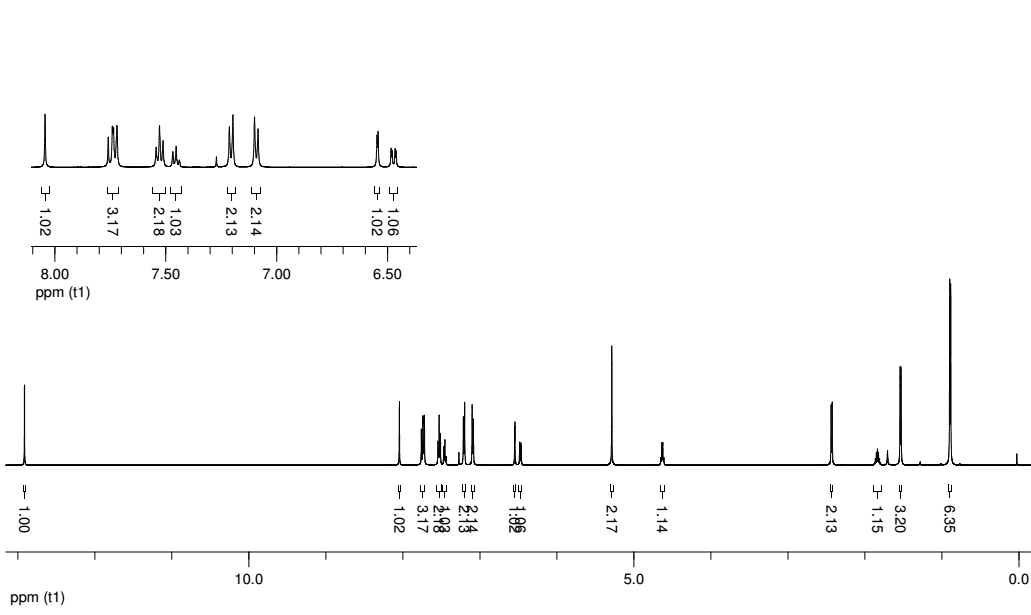


^13^C- NMR of compound 1-(2-hydroxy-4-((1-phenyl-1H-1,2,3-triazol-4-yl)methoxy)phenyl)-2-(4-isobutylphenyl)propan-1-one (**13g**).


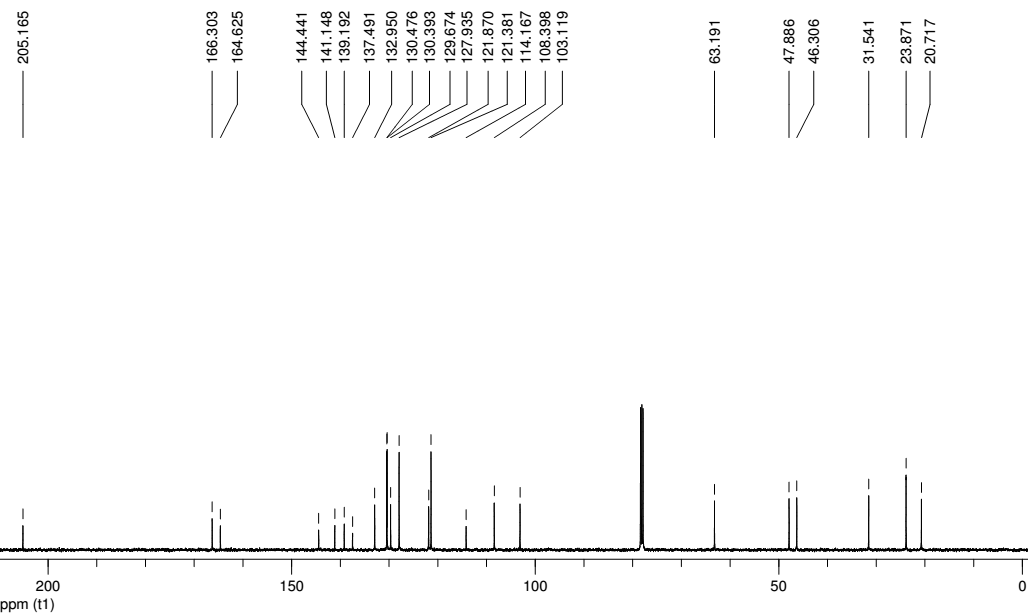


^1^H-NMR of compound 1-(2-hydroxy-4-((1-(p-tolyl)-1H-1,2,3-triazol-4-yl)methoxy)phenyl)-2-(4-isobutylphenyl)propan-1-one (**13h**).


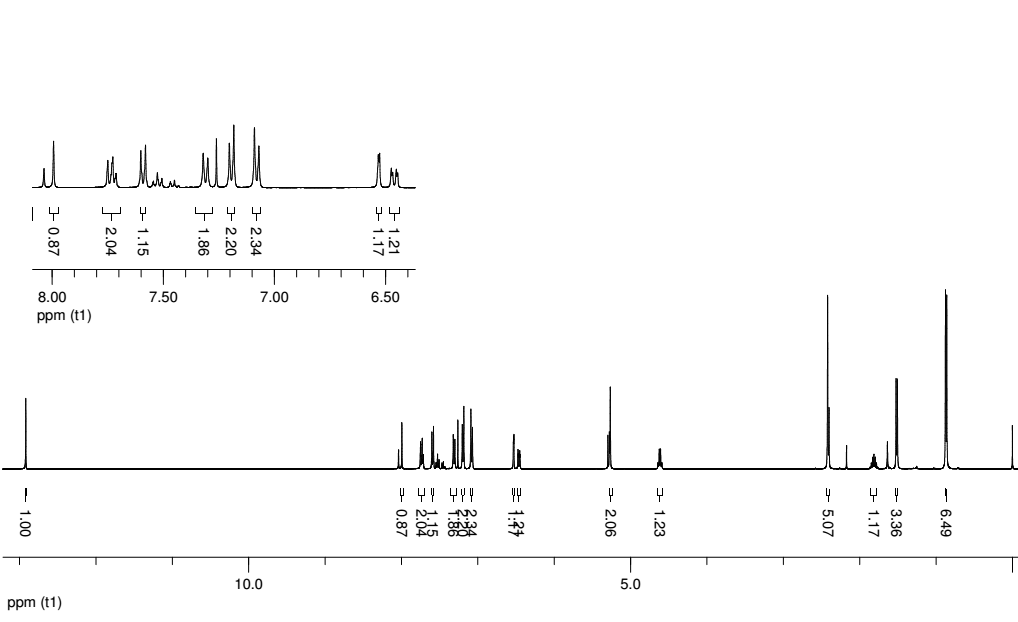


^13^C- NMR of compound 1-(2-hydroxy-4-((1-(p-tolyl)-1H-1,2,3-triazol-4-yl)methoxy)phenyl)-2-(4-isobutylphenyl)propan-1-one (**13h**).


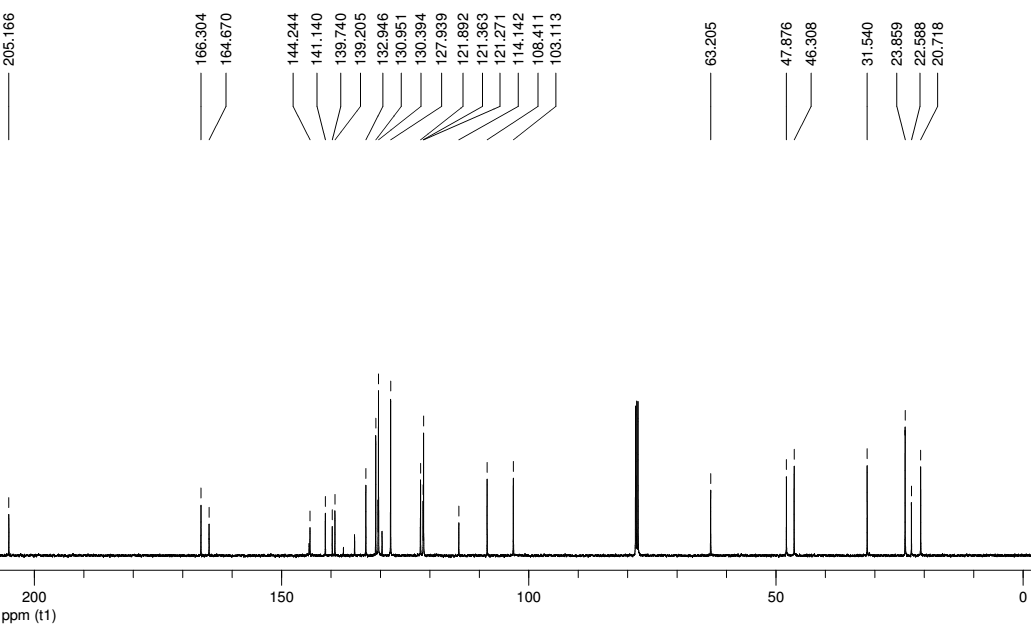


^1^H-NMR of compound 1-(4-((1-(3-chlorophenyl)-1H-1,2,3-triazol-4-yl)methoxy)-2-hydroxyphenyl)-2-(4-isobutylphenyl)propan-1-one (**13i**).


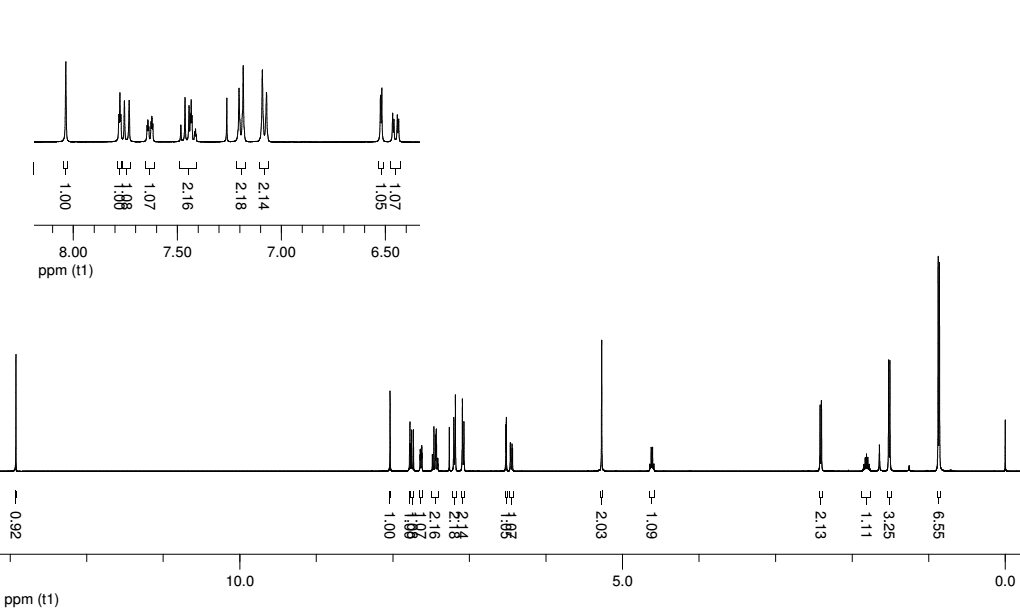

^13^C- NMR of compound 1-(4-((1-(3-chlorophenyl)-1H-1,2,3-triazol-4-yl)methoxy)-2-hydroxyphenyl)-2-(4-isobutylphenyl)propan-1-one (**13i**).
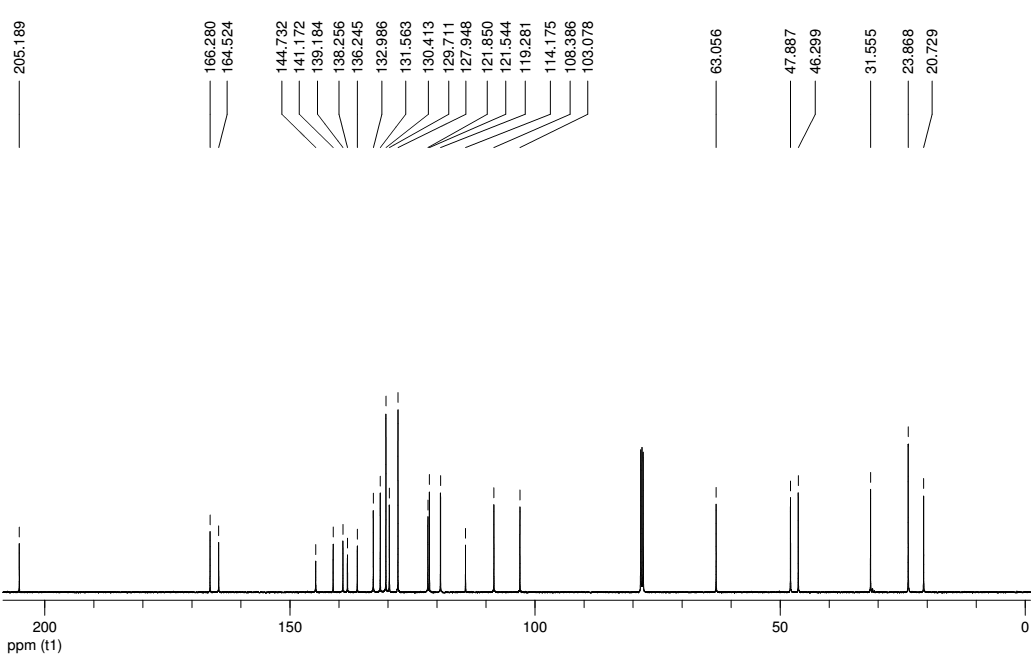


^1^H-NMR of compound 1-(4-((1-dodecyl-1*H*-1,2,3-triazol-4-yl)methoxy)-2-hydroxy phenyl)-2-(4-isobutylphenyl)propan-1-one (**13j**).


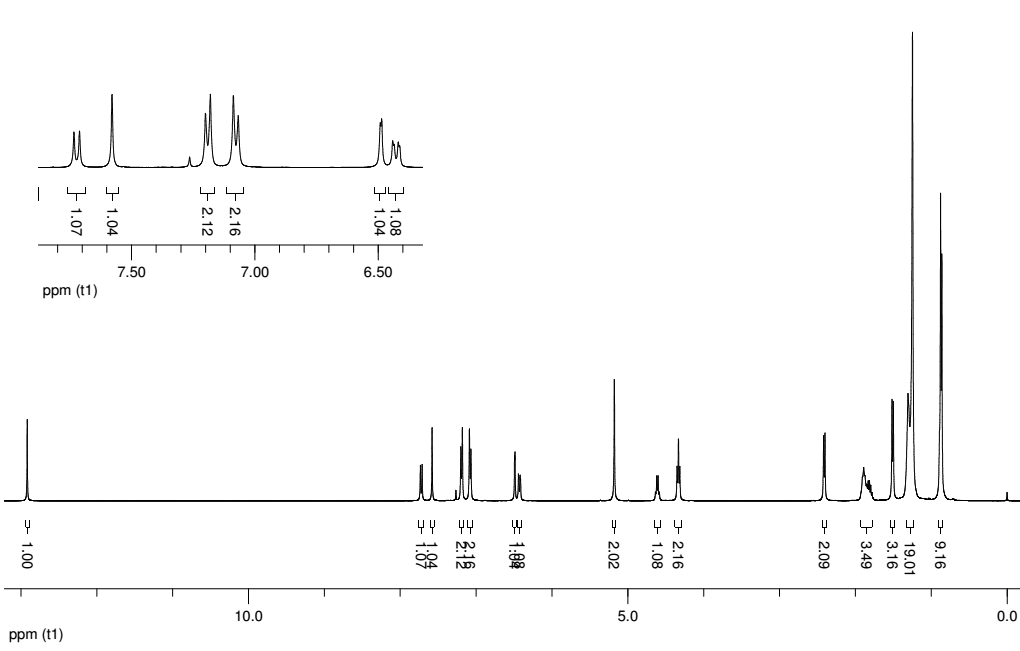

^13^C- NMR of compound 1-(4-((1-dodecyl-1*H*-1,2,3-triazol-4-yl)methoxy)-2-hydroxy phenyl)-2-(4-isobutylphenyl)propan-1-one (**13j**).
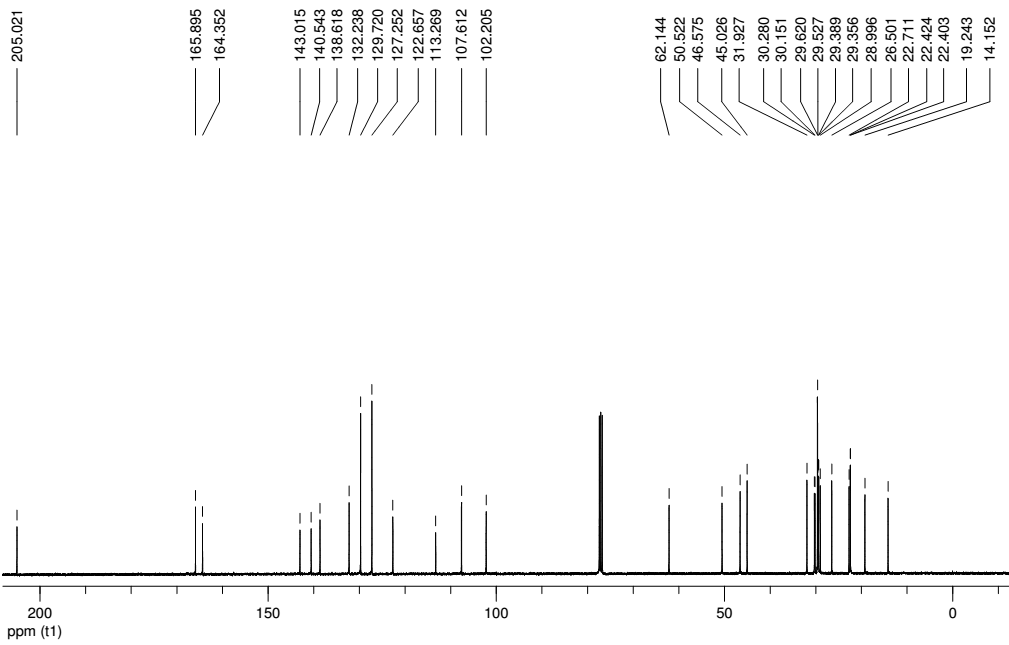


^1^H-NMR of compound 1-(2-hydroxy-4-((1-(3-nitrophenyl)-1H-1,2,3-triazol-4-yl)methoxy)phenyl)-2-(4-isobutylphenyl)propan-1-one (**13k**).


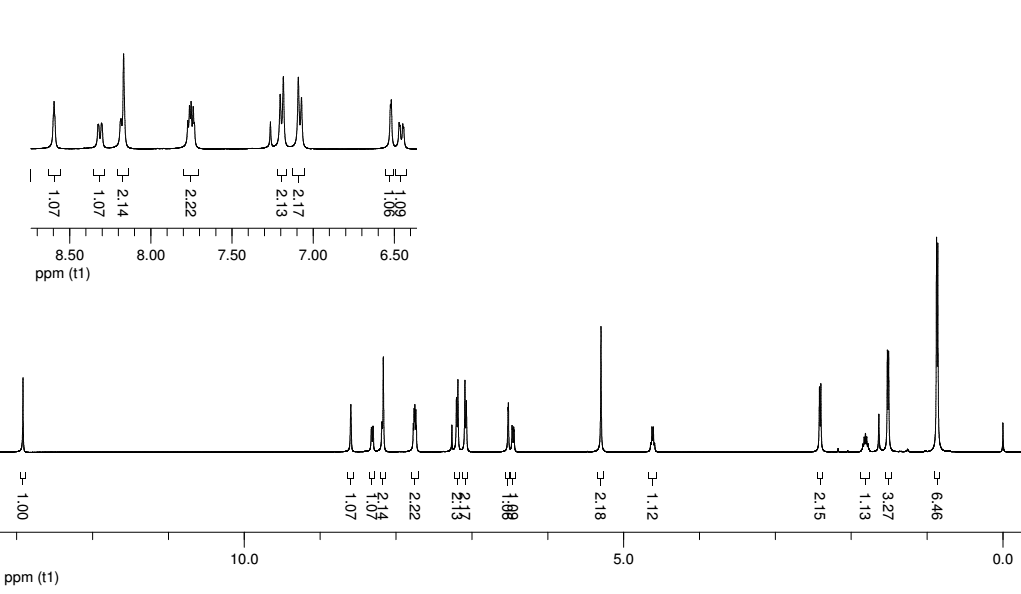


^13^C- NMR of compound 1-(2-hydroxy-4-((1-(3-nitrophenyl)-1H-1,2,3-triazol-4-yl)methoxy)phenyl)-2-(4-isobutylphenyl)propan-1-one (**13k**).
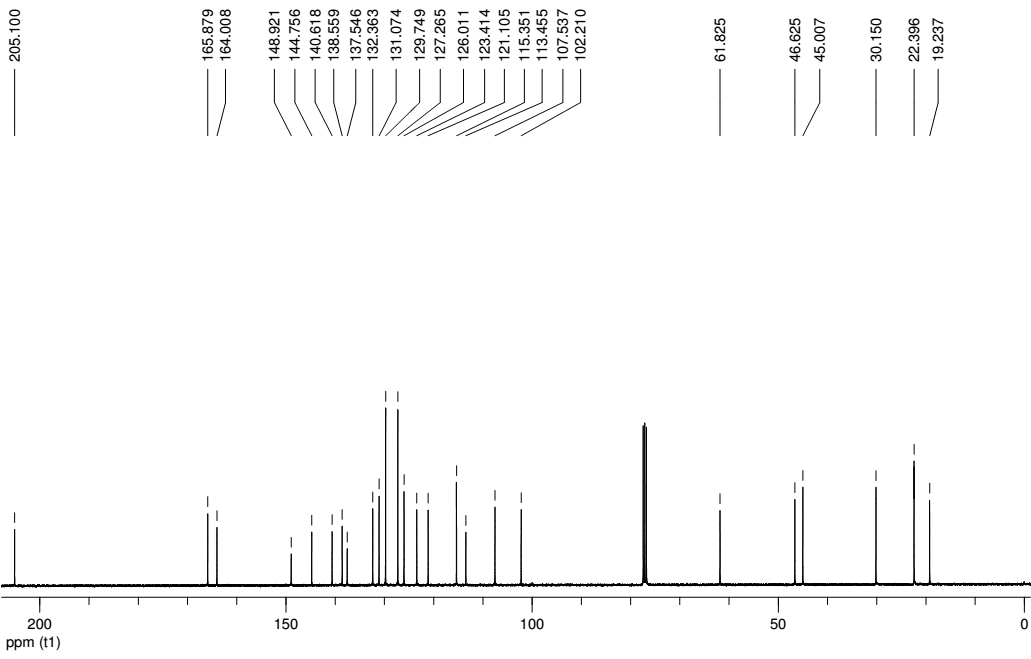


^1^H-NMR of compound 1-(2-hydroxy-4-((1-(4-nitrophenyl)-1H-1,2,3-triazol-4-yl)methoxy)phenyl)-2-(4-isobutylphenyl)propan-1-one (**13l**).


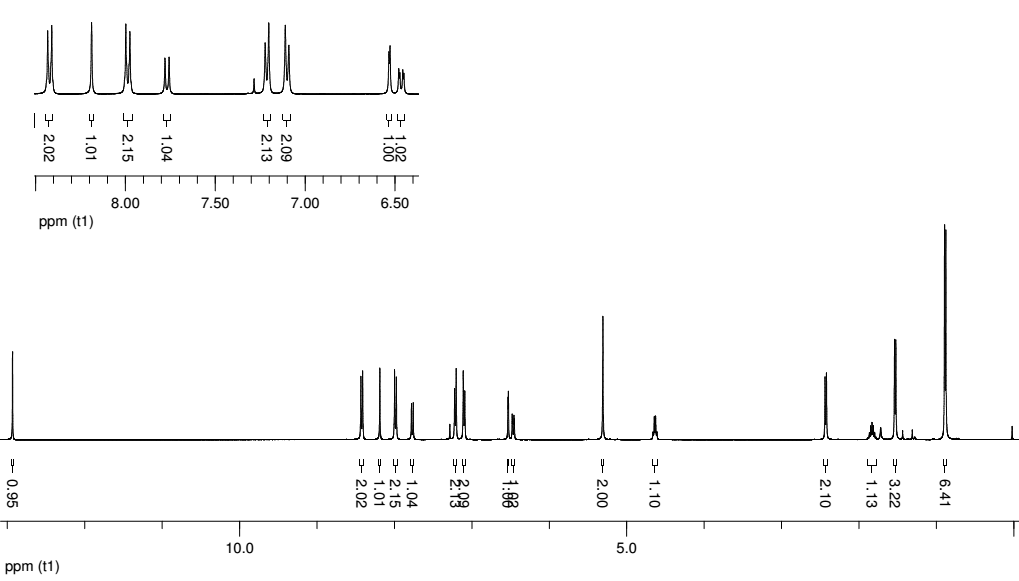


^13^C- NMR of compound 1-(2-hydroxy-4-((1-(4-nitrophenyl)-1H-1,2,3-triazol-4-yl)methoxy)phenyl)-2-(4-isobutylphenyl)propan-1-one (**13l**).


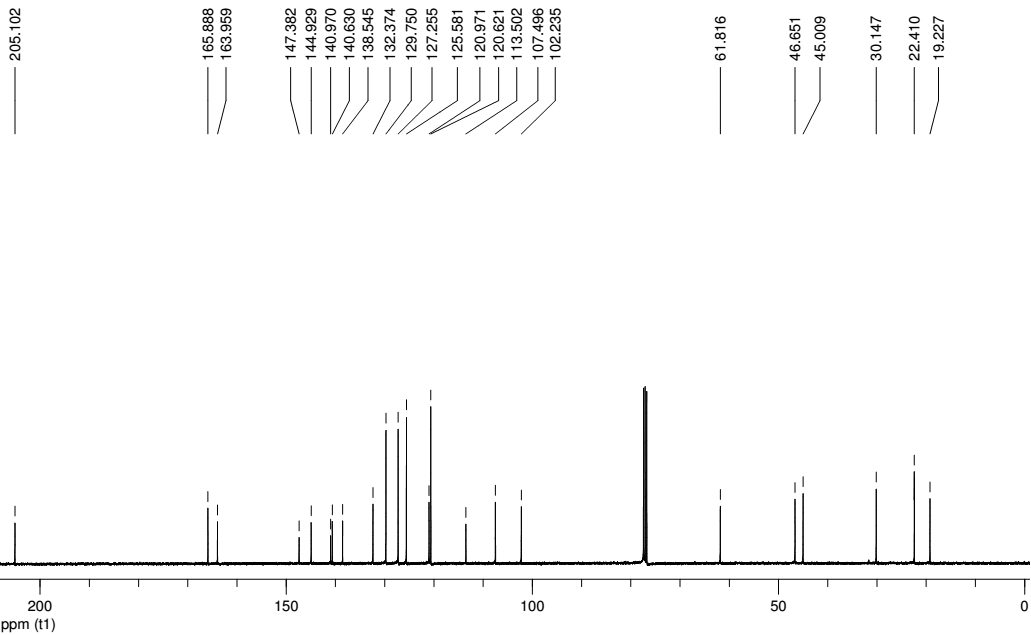


^1^H-NMR of compound 1-(4-((1-cyclohexyl-1H-1,2,3-triazol-4-yl)methoxy)-2-hydroxyphenyl)-2-(4-isobutylphenyl)propan-1-one (**13m**).


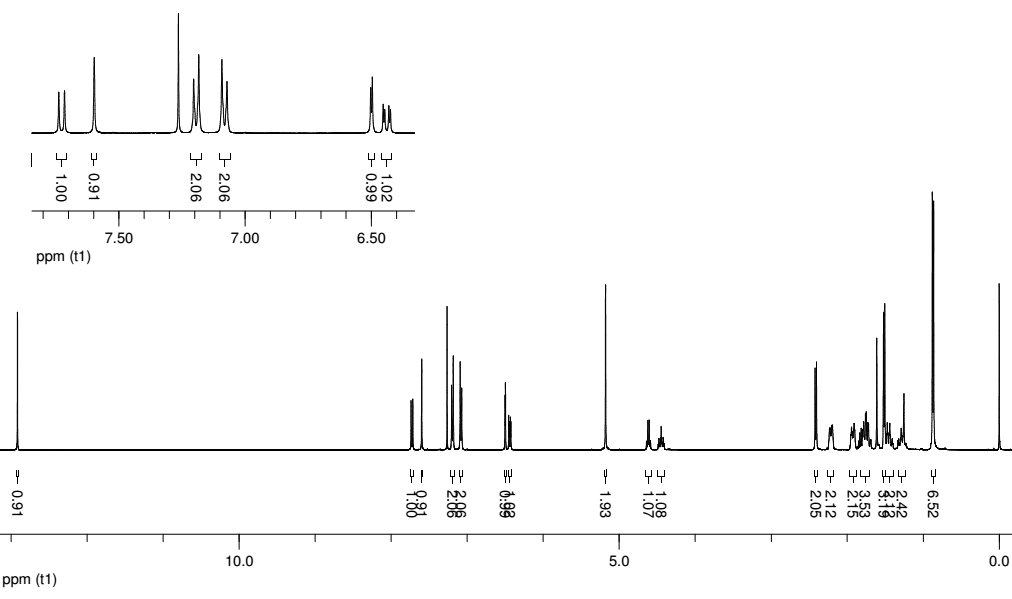


^13^C- NMR of compound 1-(4-((1-cyclohexyl-1H-1,2,3-triazol-4-yl)methoxy)-2-hydroxyphenyl)-2-(4-isobutylphenyl)propan-1-one (**13m**).


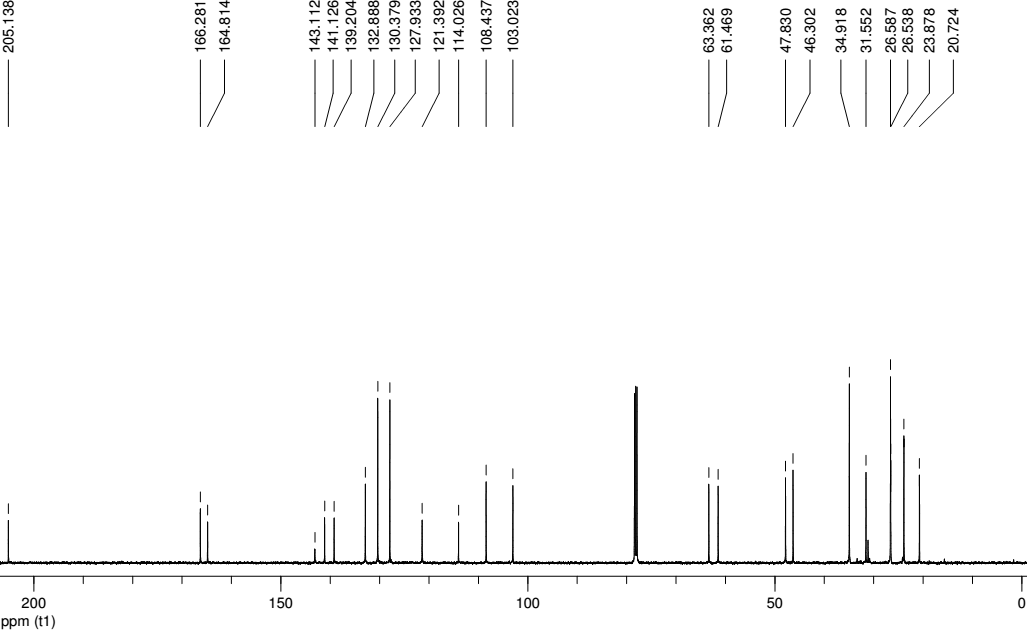


^1^H-NMR of compound 1-(4-((1-cyclopentyl-1H-1,2,3-triazol-4-yl)methoxy)-2-hydroxyphenyl)-2-(4-isobutylphenyl)propan-1-one (**13n**).


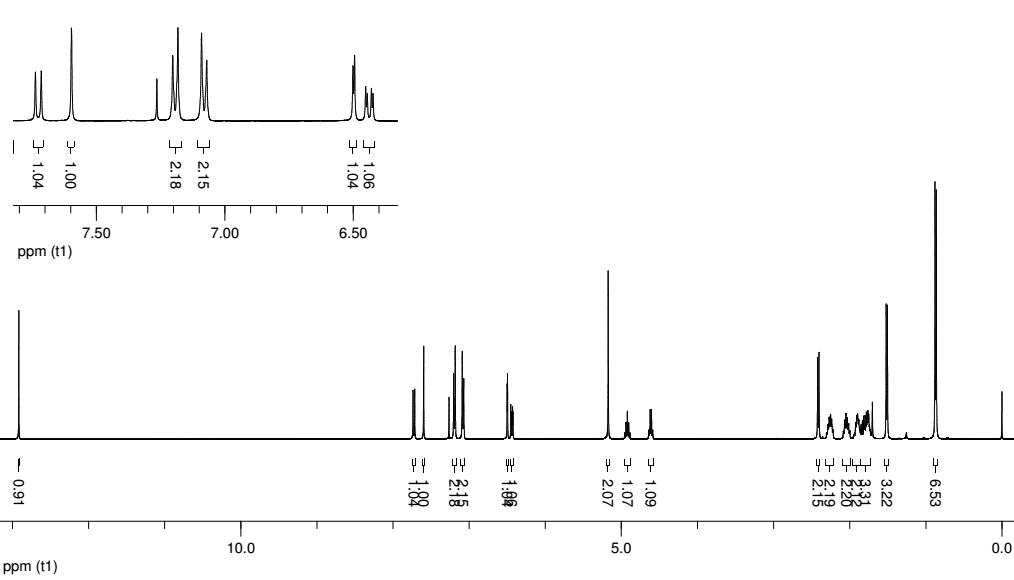


^1^H-NMR of compound 1-(2-hydroxy-4-((1-(4-nitrobenzyl)-1H-1,2,3-triazol-4-yl)methoxy)phenyl)-2-(4-isobutylphenyl)propan-1-one (**13o**).


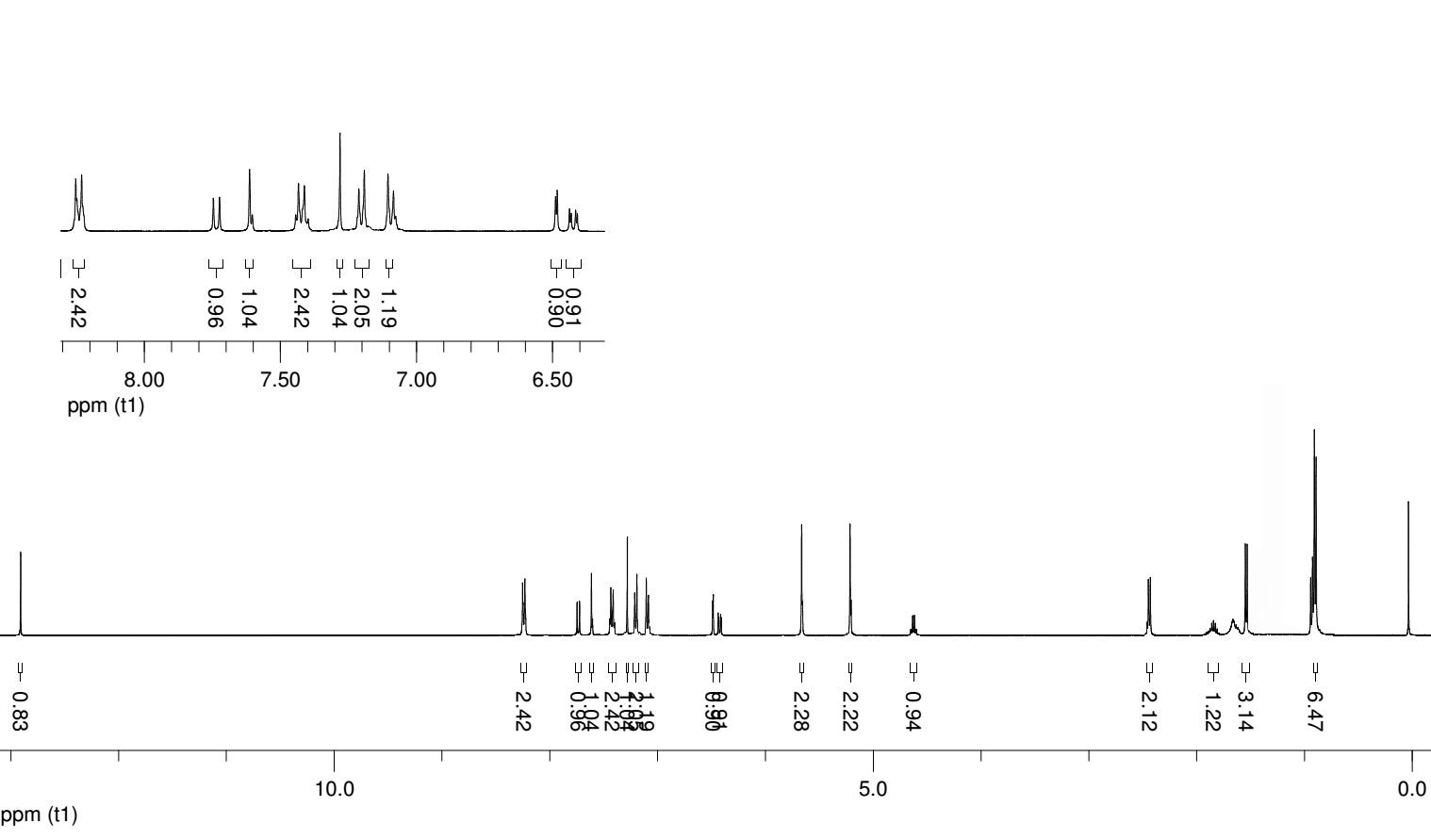

^13^C- NMR of compound 1-(2-hydroxy-4-((1-(4-nitrobenzyl)-1H-1,2,3-triazol-4-yl)methoxy)phenyl)-2-(4-isobutylphenyl)propan-1-one (**13o**).
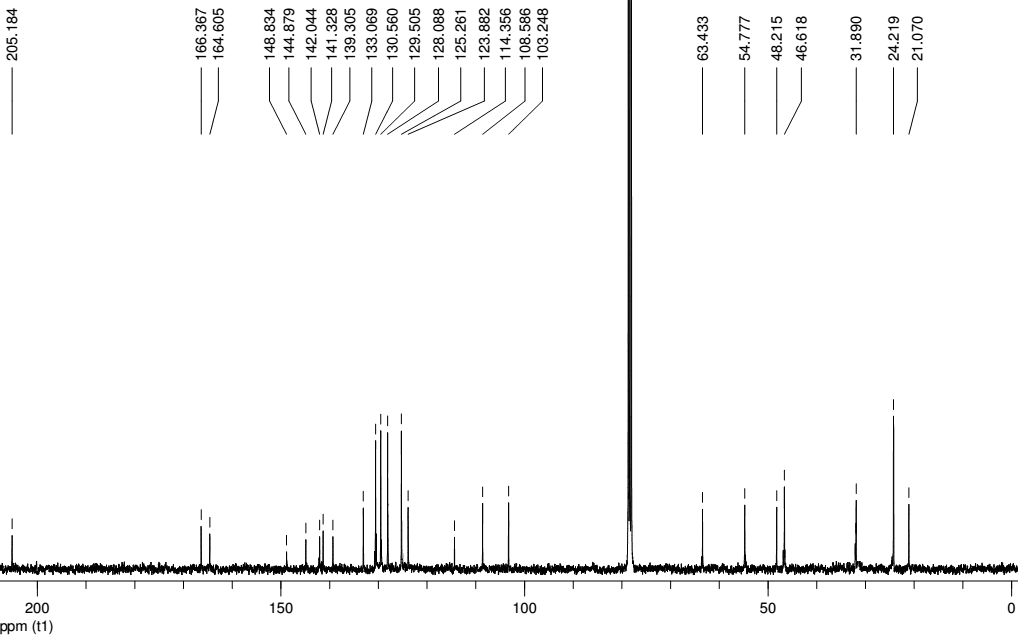


^1^H-NMR of compound 1-(4-((1-(4-bromophenyl)-1H-1,2,3-triazol-4-yl)methoxy)-2-hydroxyphenyl)-2-(4-isobutylphenyl)propan-1-one (**13p**).


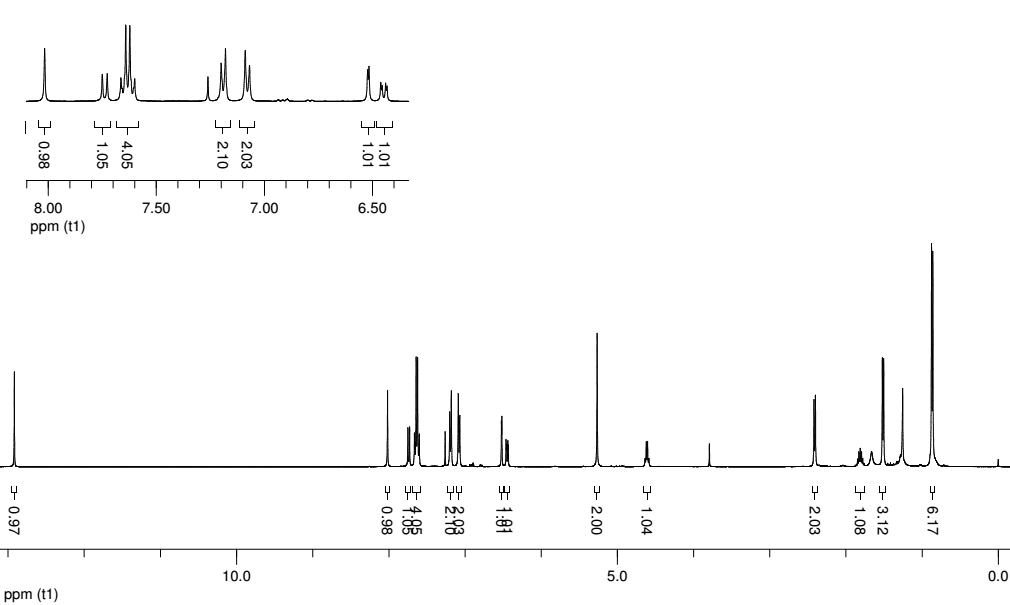


^13^C- NMR of compound 1-(4-((1-(4-bromophenyl)-1H-1,2,3-triazol-4-yl)methoxy)-2-hydroxyphenyl)-2-(4-isobutylphenyl)propan-1-one (**13p**).


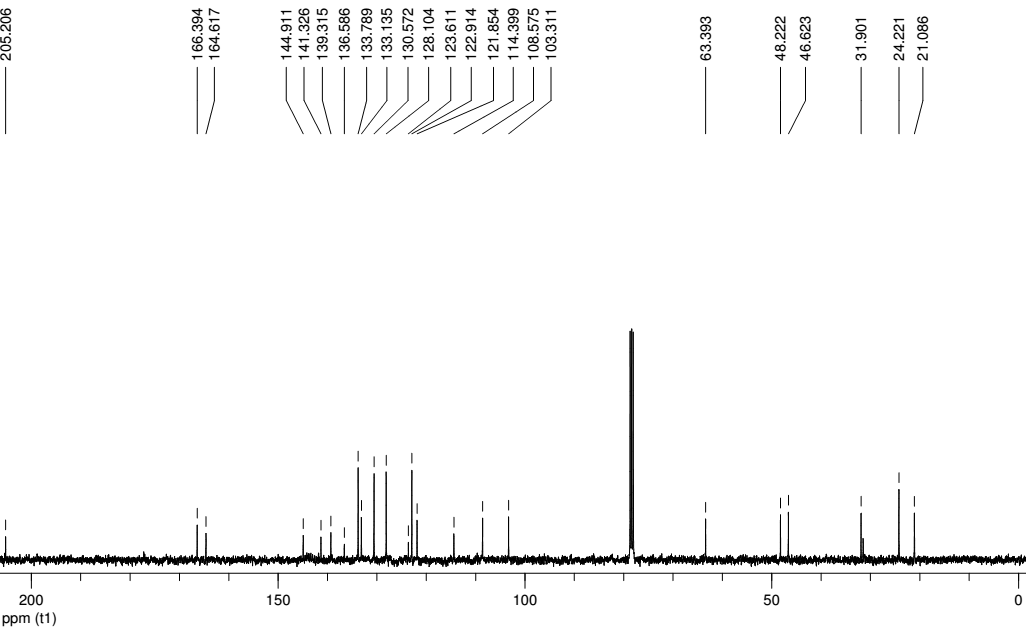


^1^H-NMR of compound 1-(4-((1-(2-chlorophenyl)-1H-1,2,3-triazol-4-yl)methoxy)-2-hydroxyphenyl)-2-(4-isobutylphenyl)propan-1-one (**13q**).


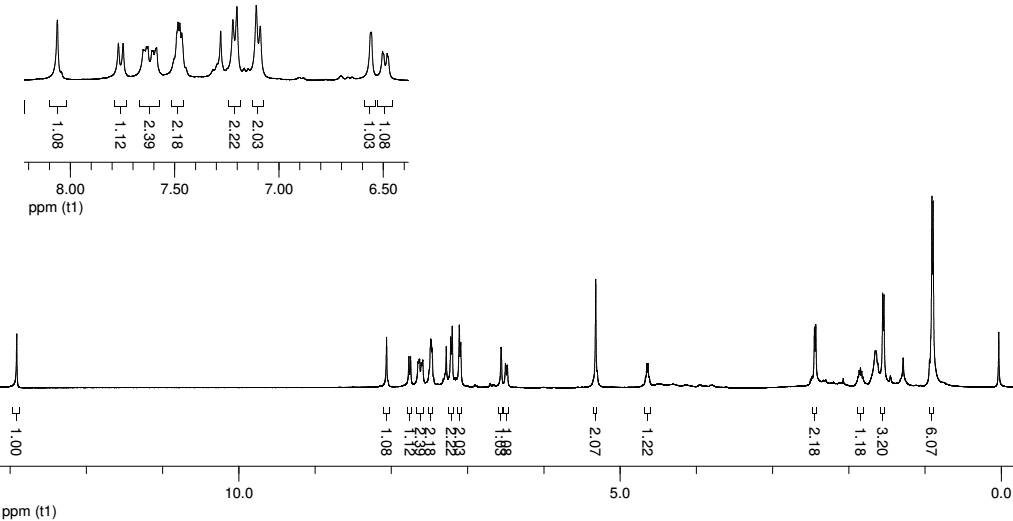


^1^H-NMR of compound 1-(4-((1-(2-chlorophenyl)-1H-1,2,3-triazol-4-yl)methoxy)-2-hydroxyphenyl)-2-(4-isobutylphenyl)propan-1-one (**13q**).


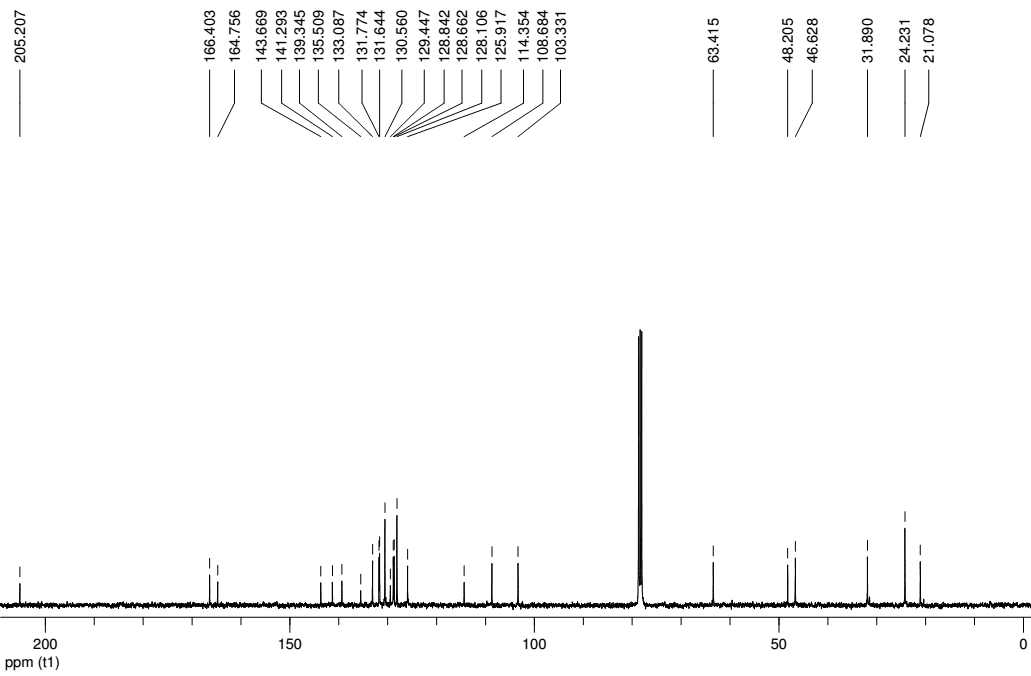


LRMS of compound **10**.


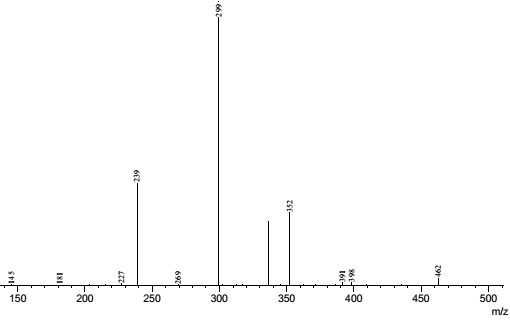


LRMS of compound **11**.
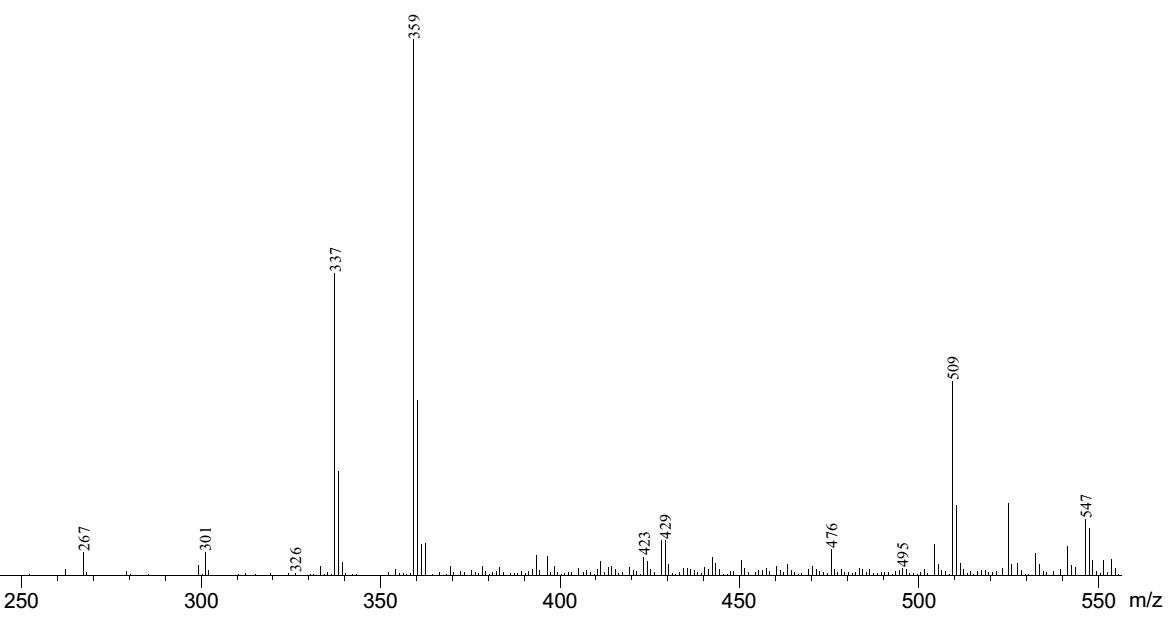


LRMS of compound **13a**.
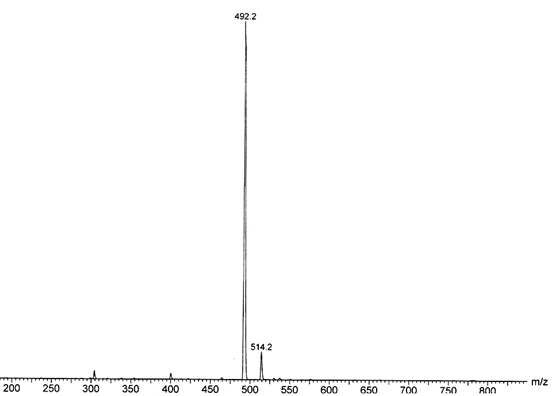


LRMS of compound **13b**.
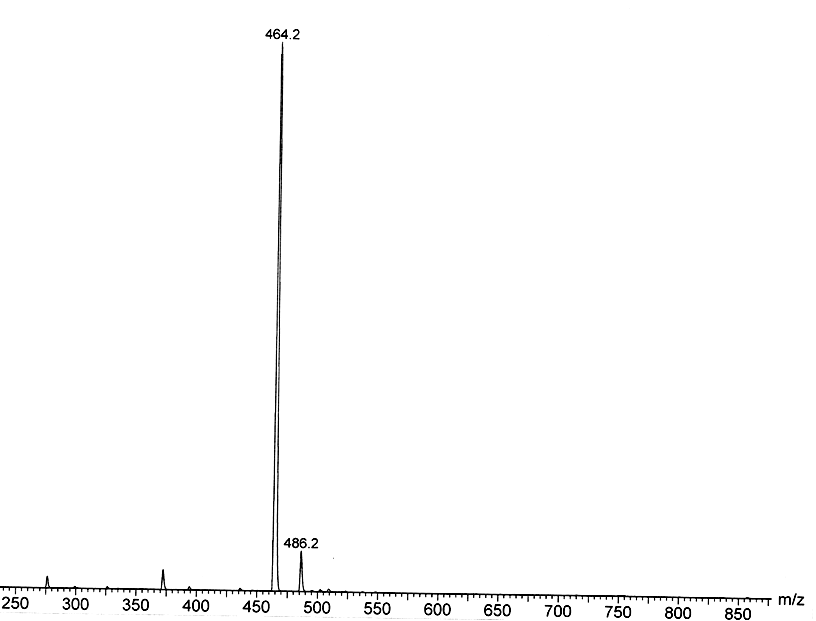


LRMS of compound **13c**.
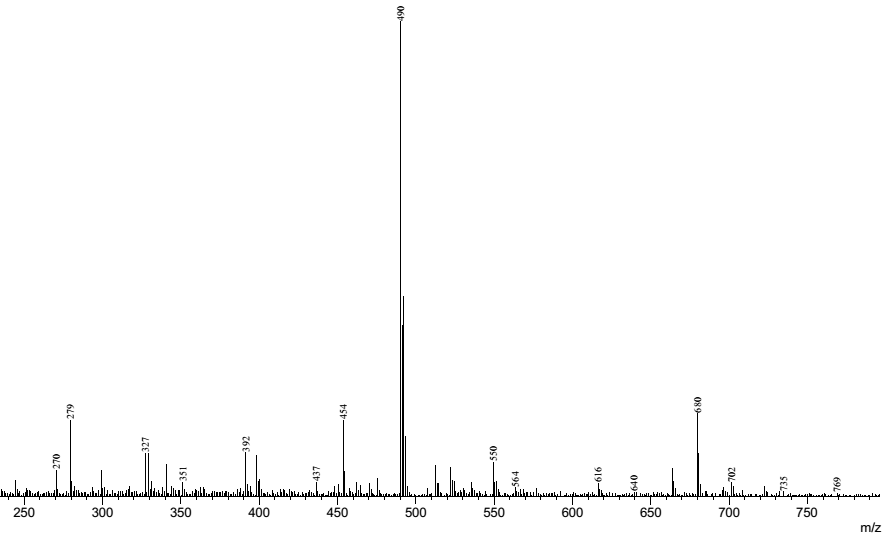

LRMS of compound **13d**.
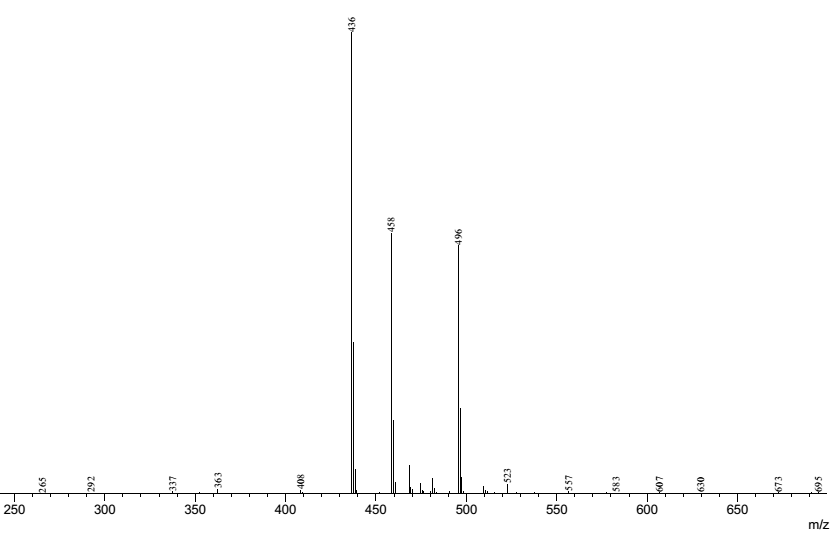


LRMS of compound **13e**.
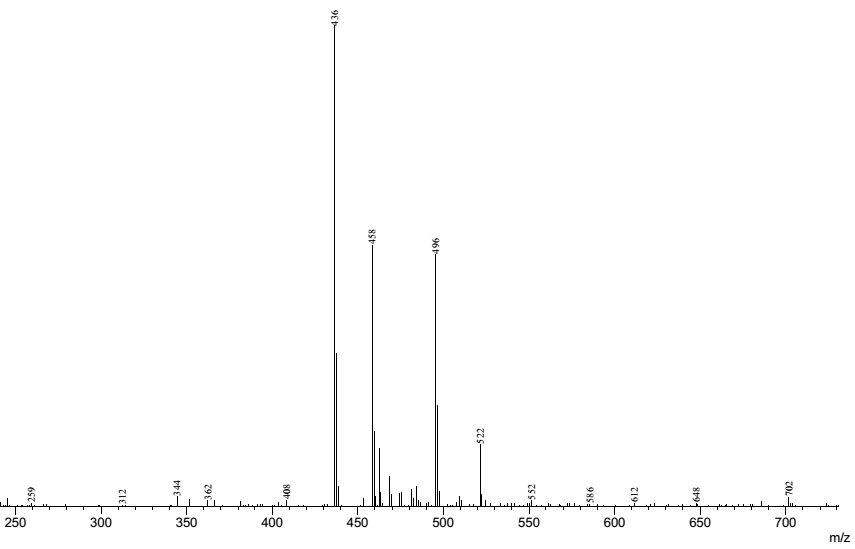


LRMS of compound **13f**.
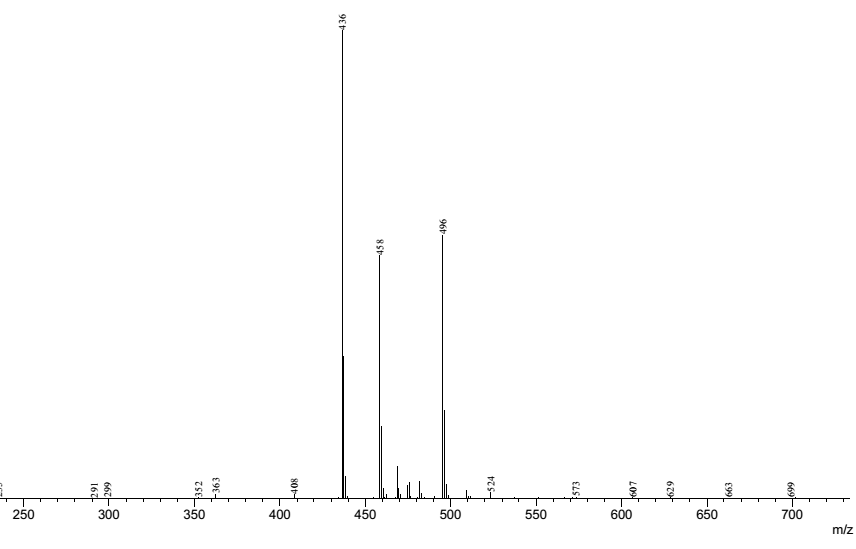


LRMS of compound **13g**.
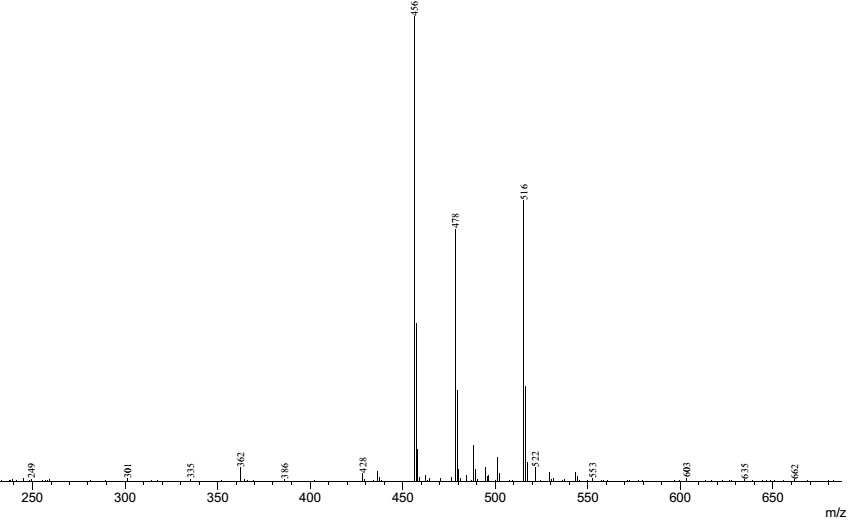


LRMS of compound **13h**.
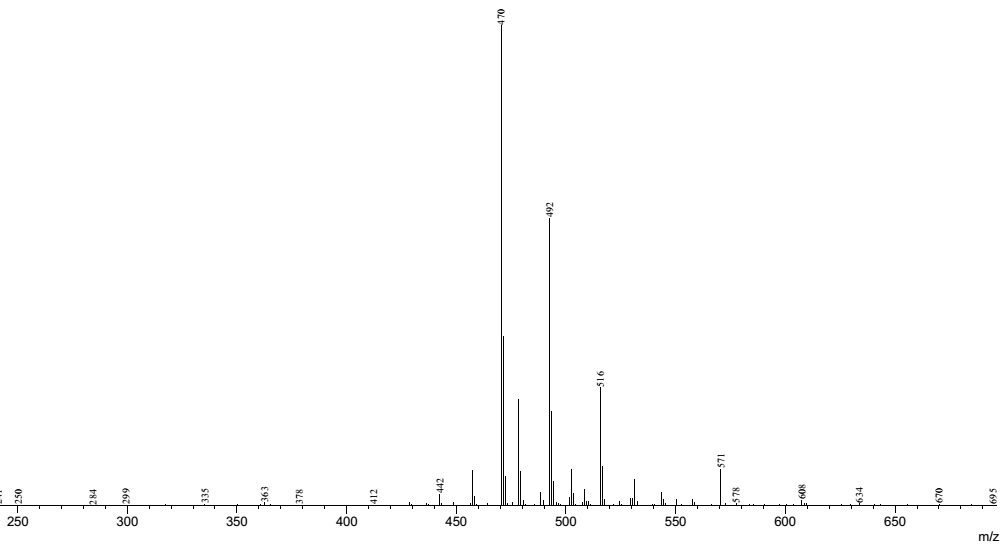


LRMS of compound **13i**.
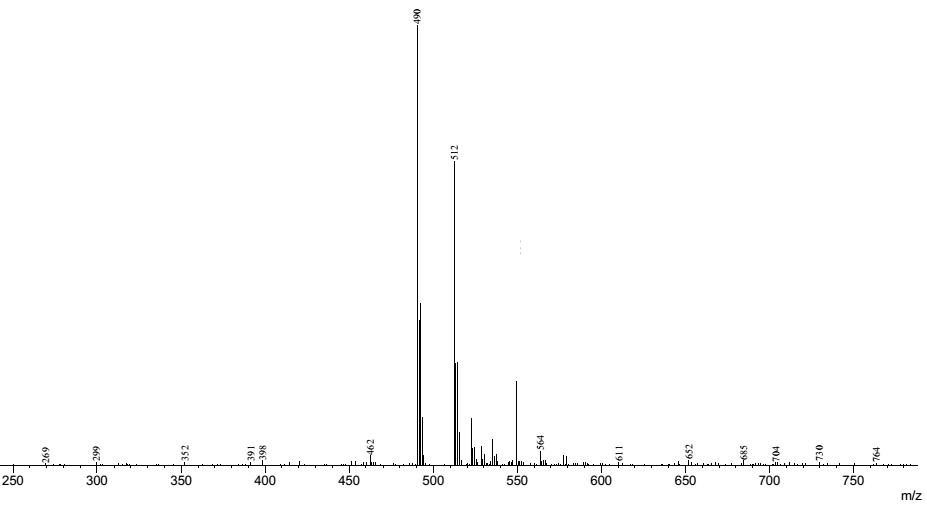


LRMS of compound **13j**.
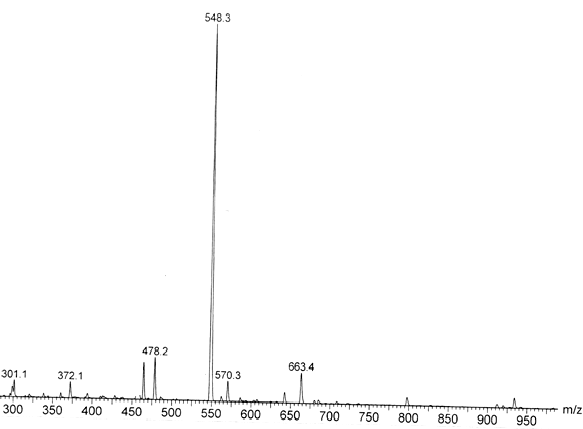


LRMS of compound **13k**.
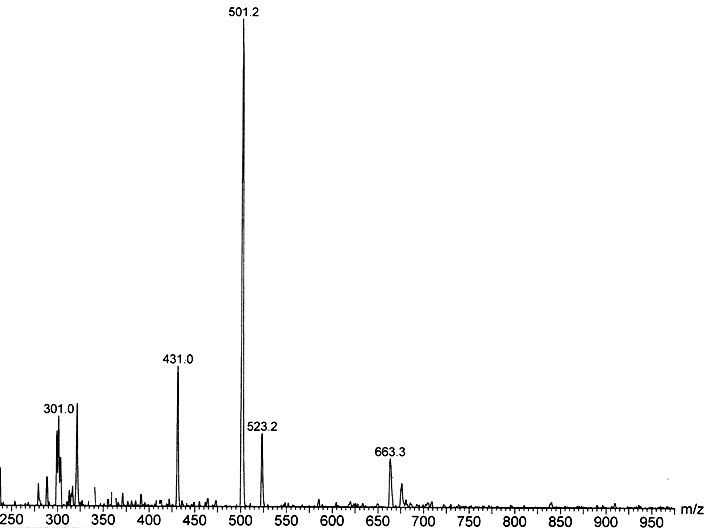


LRMS of compound **13l**.
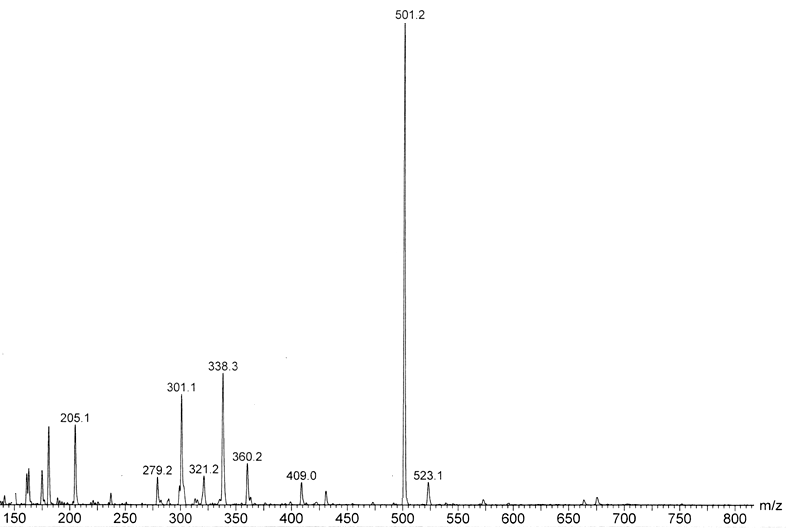


LRMS of compound **13m**.


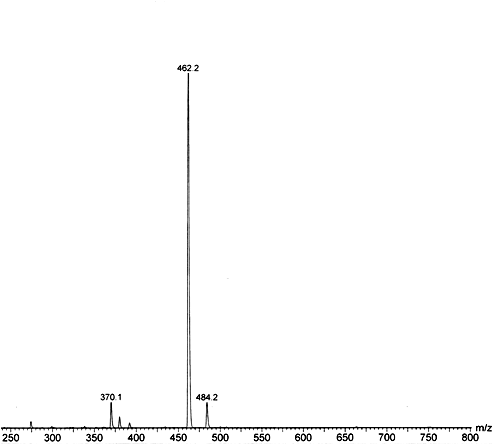


LRMS of compound **13n**.
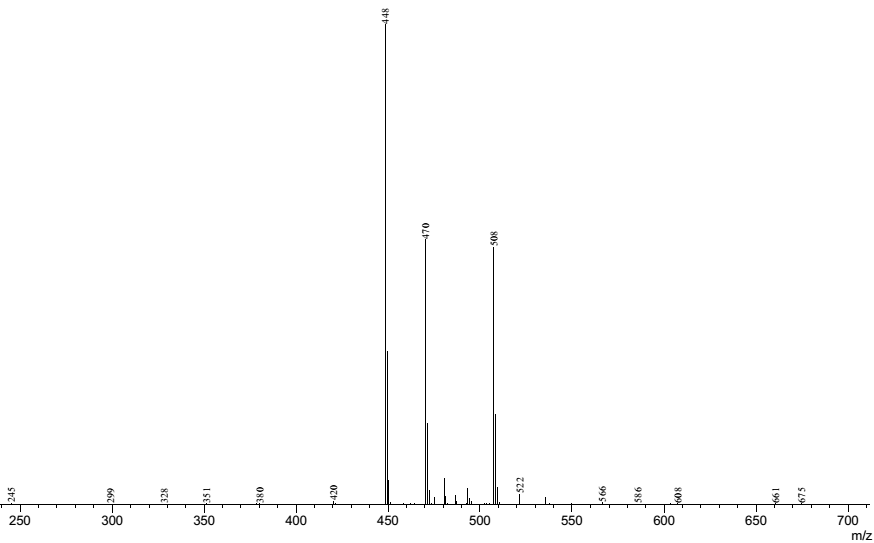


LRMS of compound **13o**.
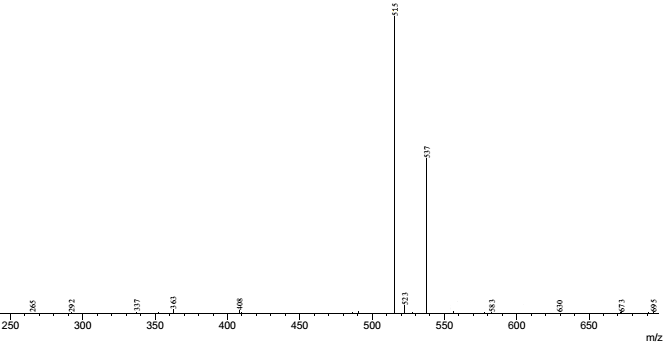


LRMS of compound **13p**.
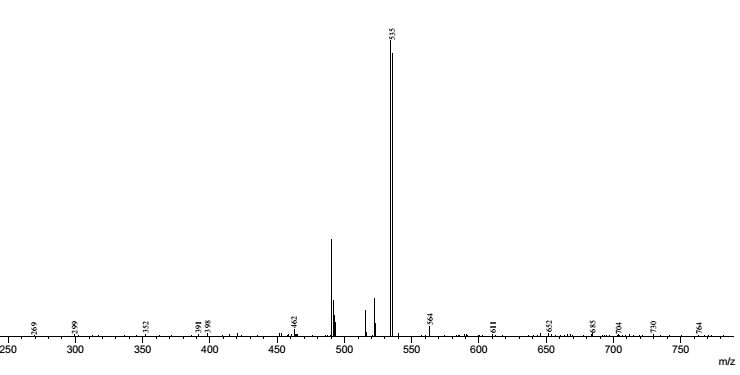


LRMS of compound **13q**.
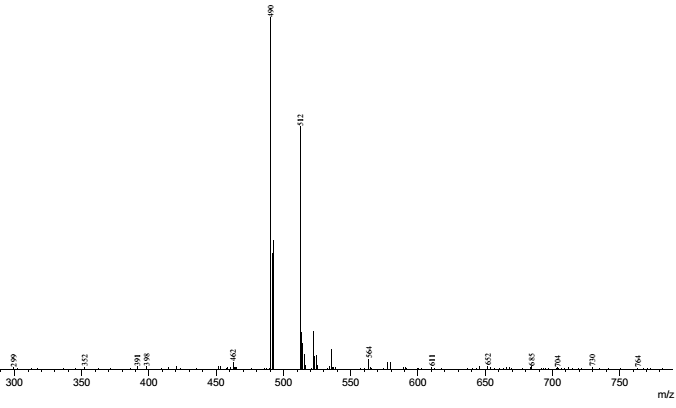


HPLC of compound **13a
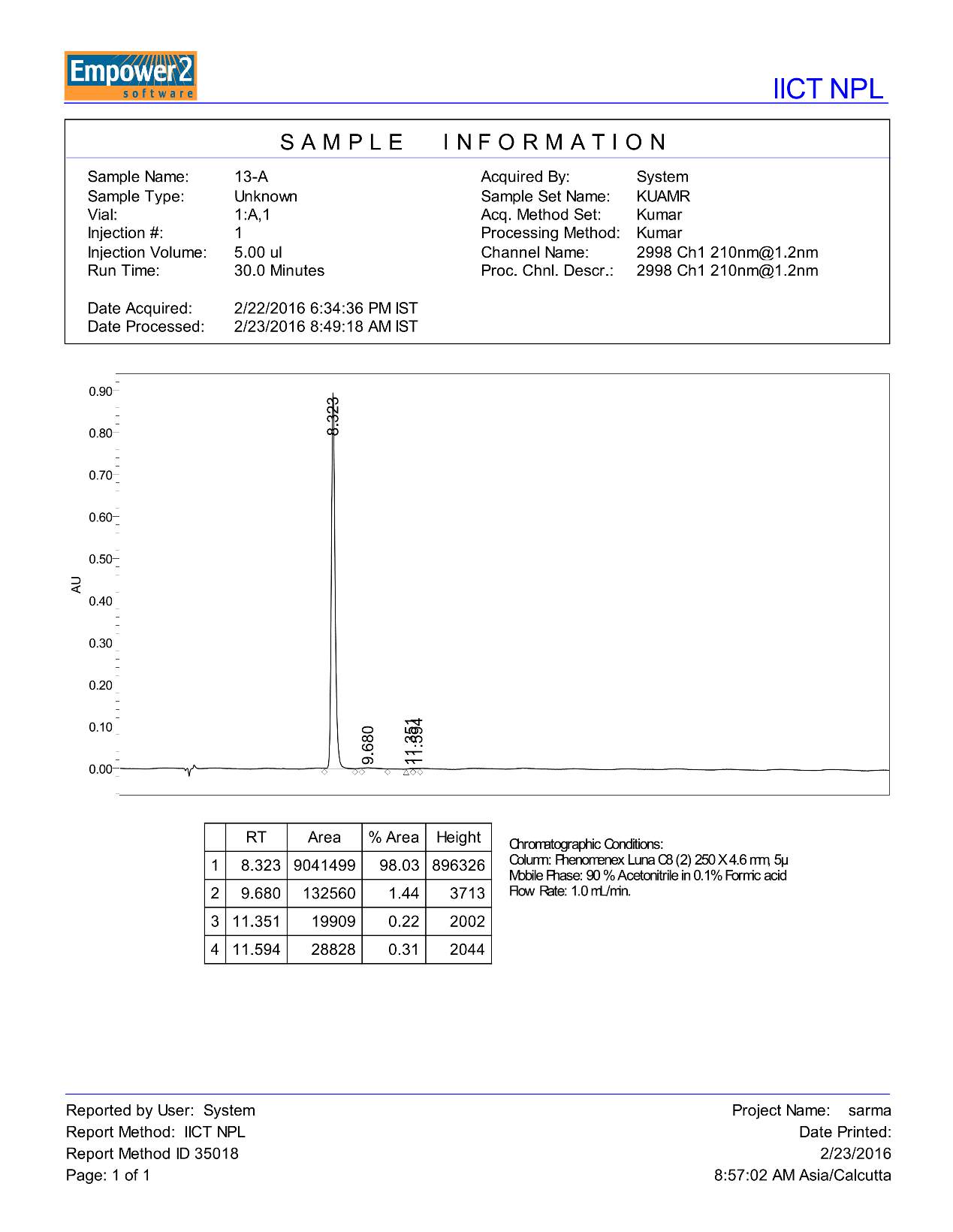
**

HPLC of compound **13b**
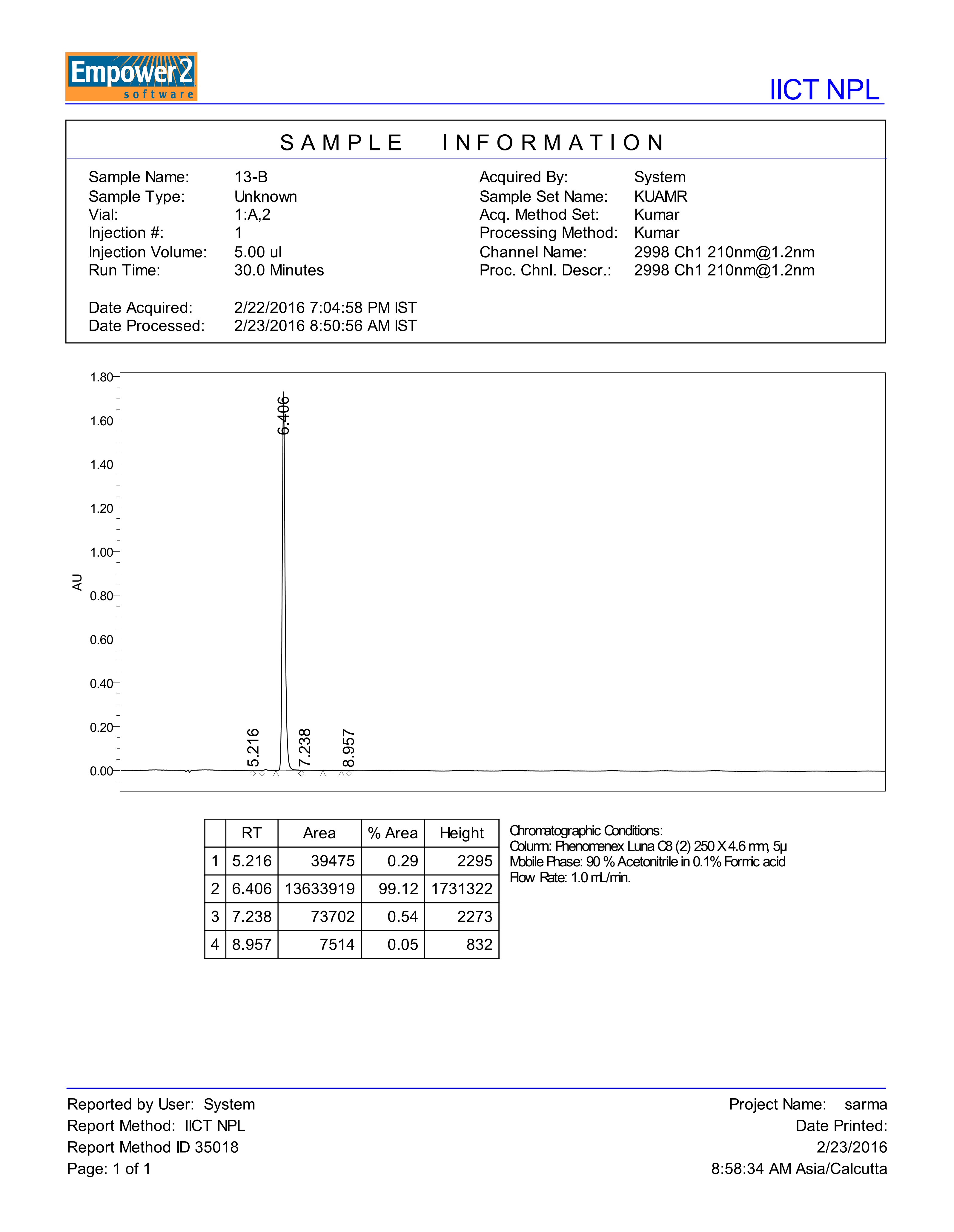


HPLC of compound **13c
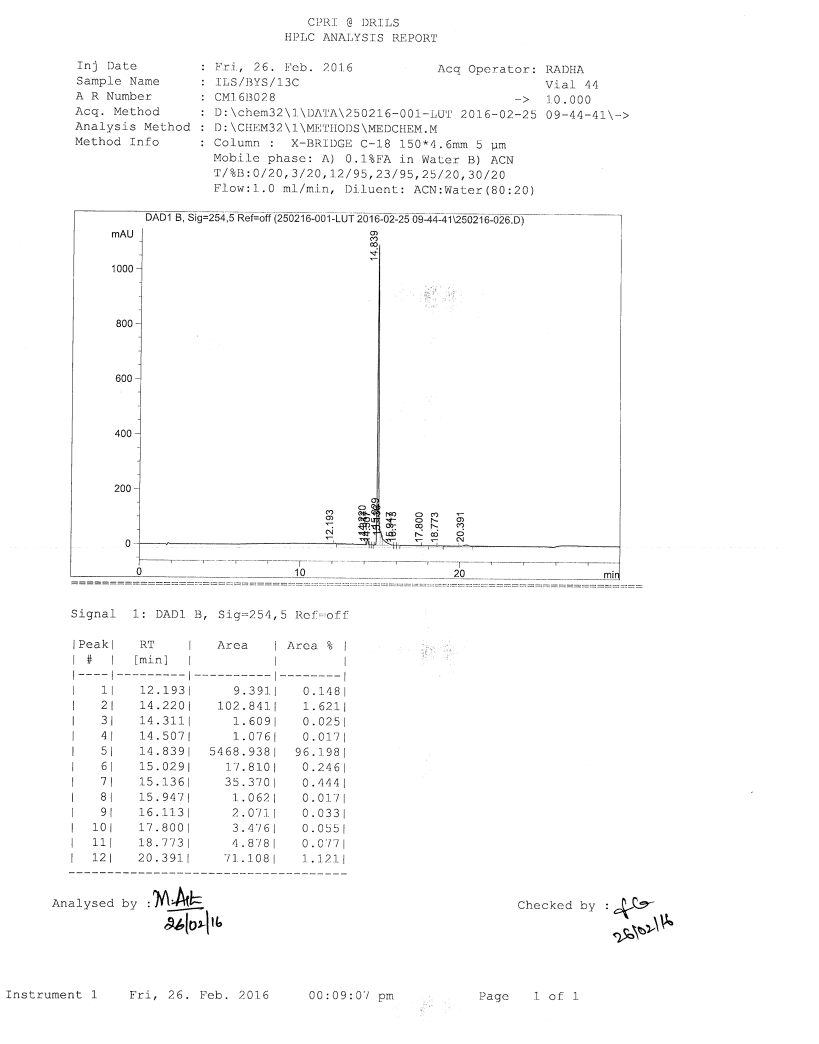
**

HPLC of compound **13e**
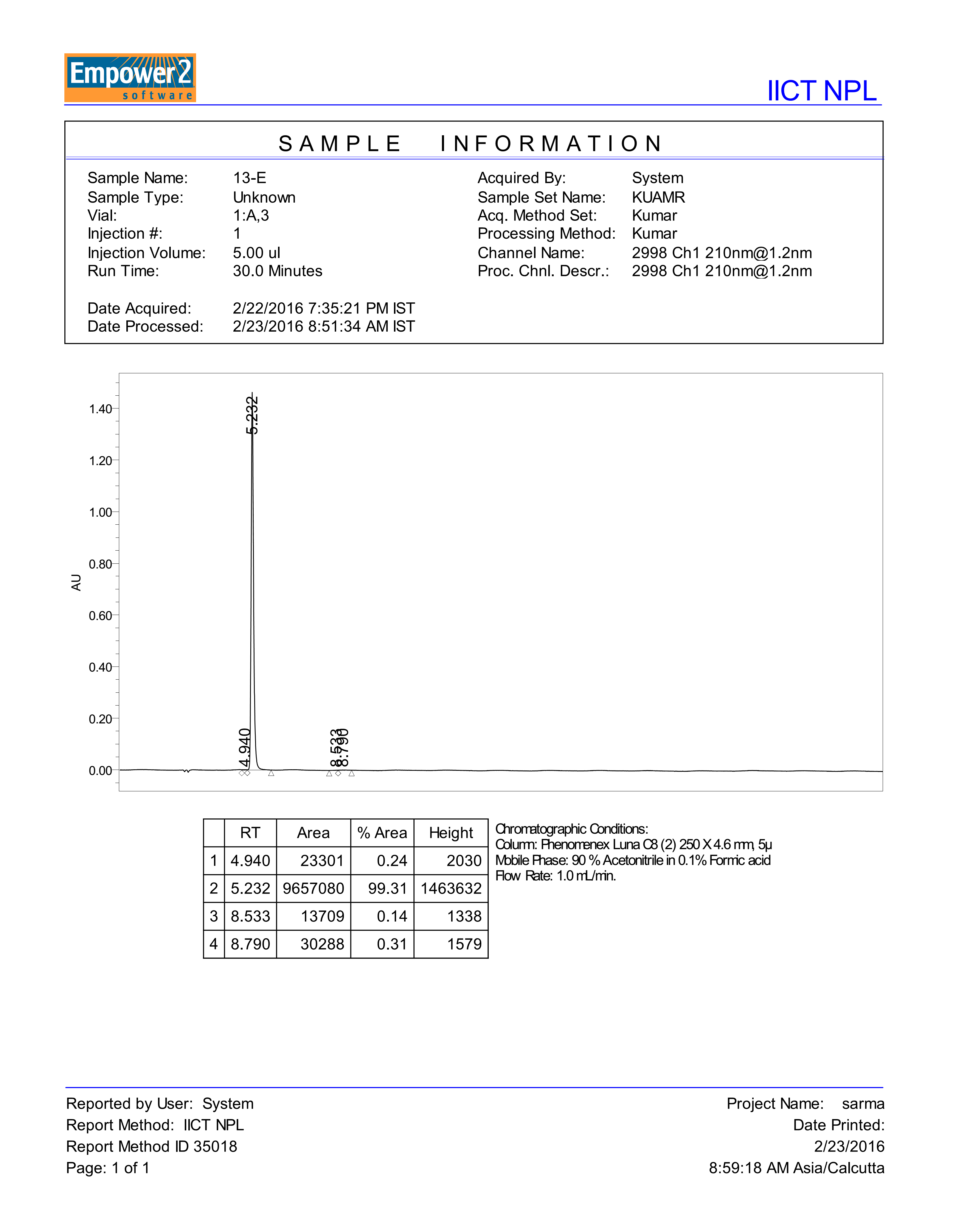


HPLC of compound **13f
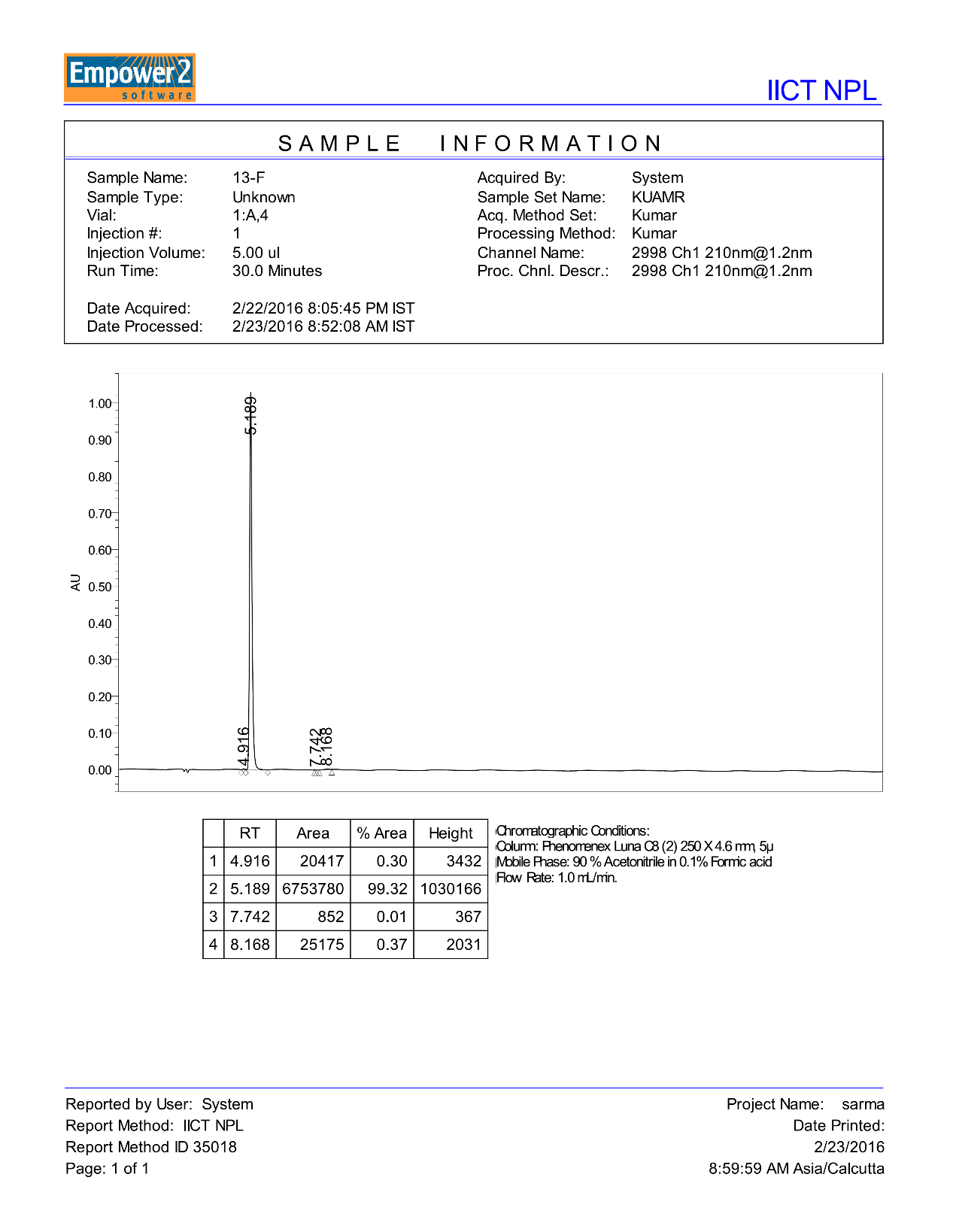
**

HPLC of compound **13g**
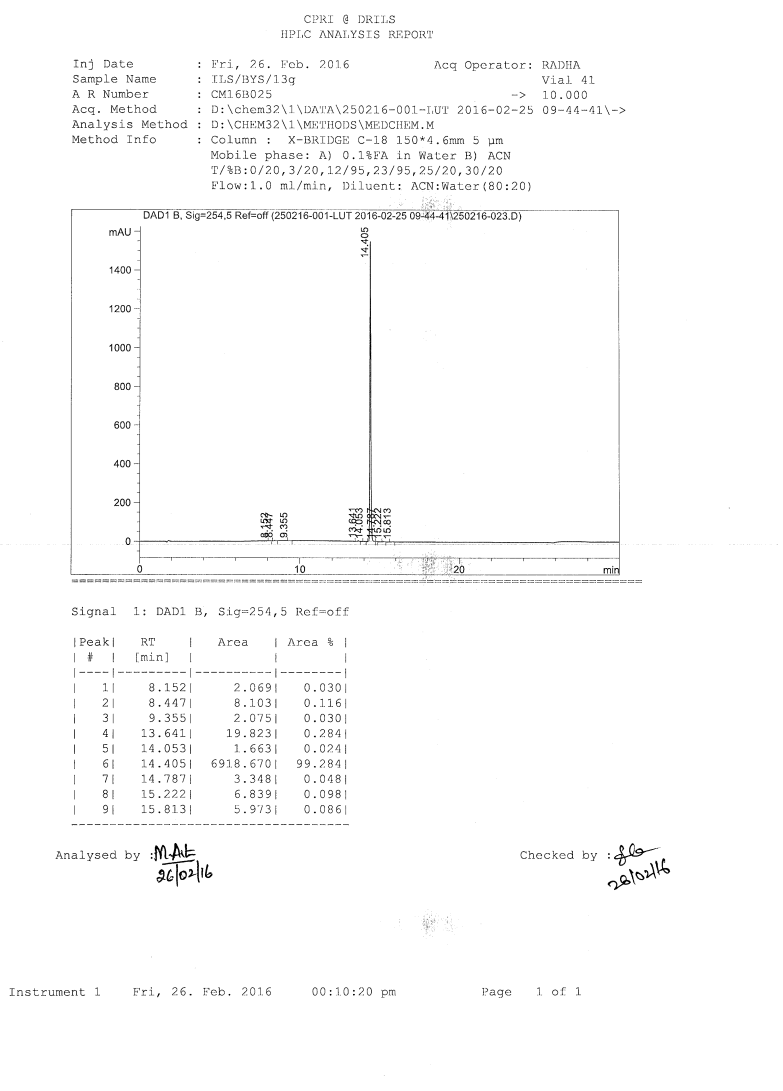


HPLC of compound **13h**
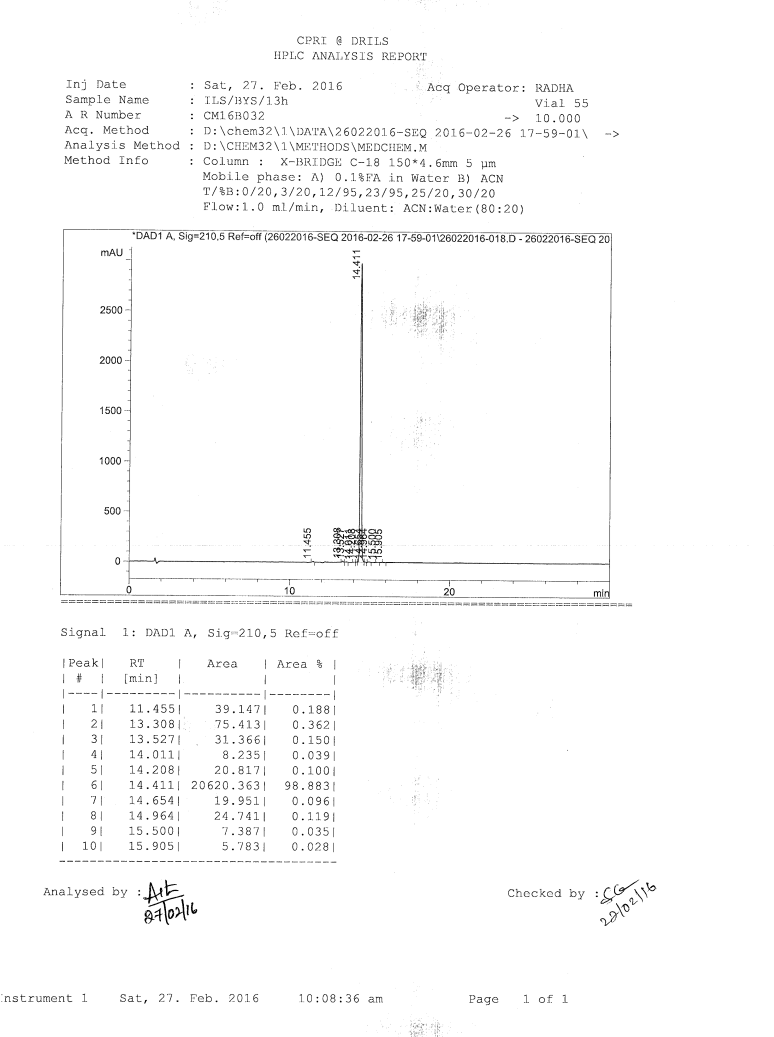


HPLC of compound **13i**
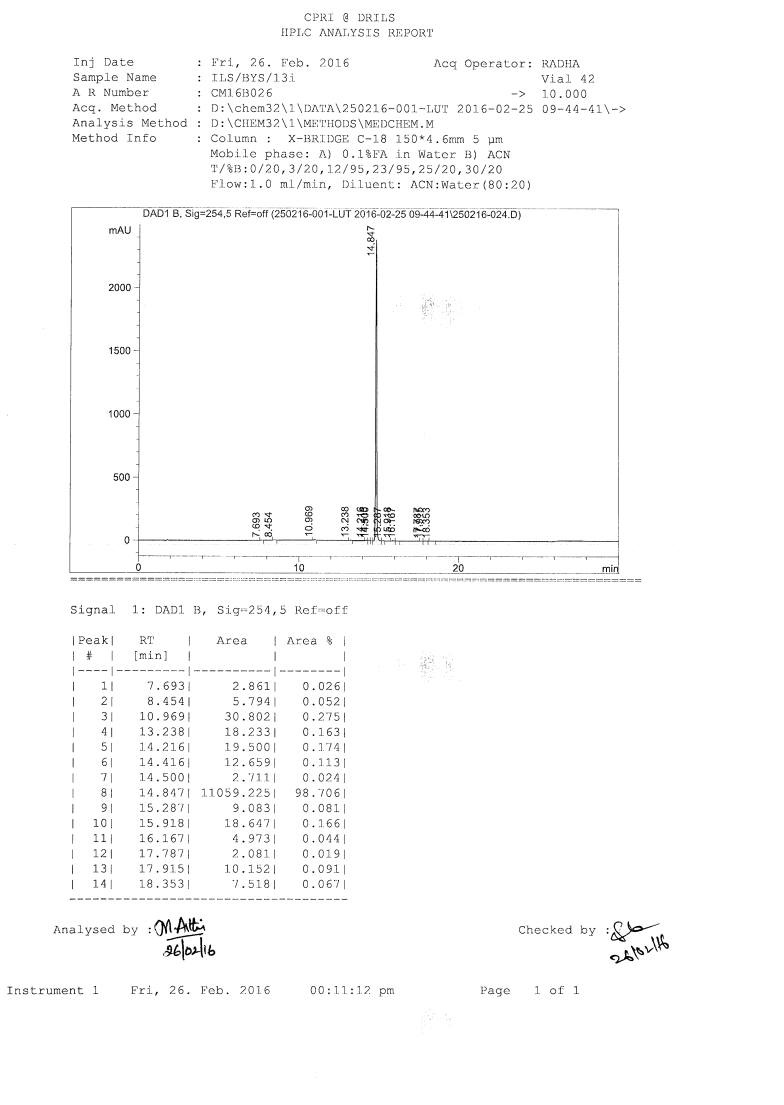


HPLC of compound **13j**
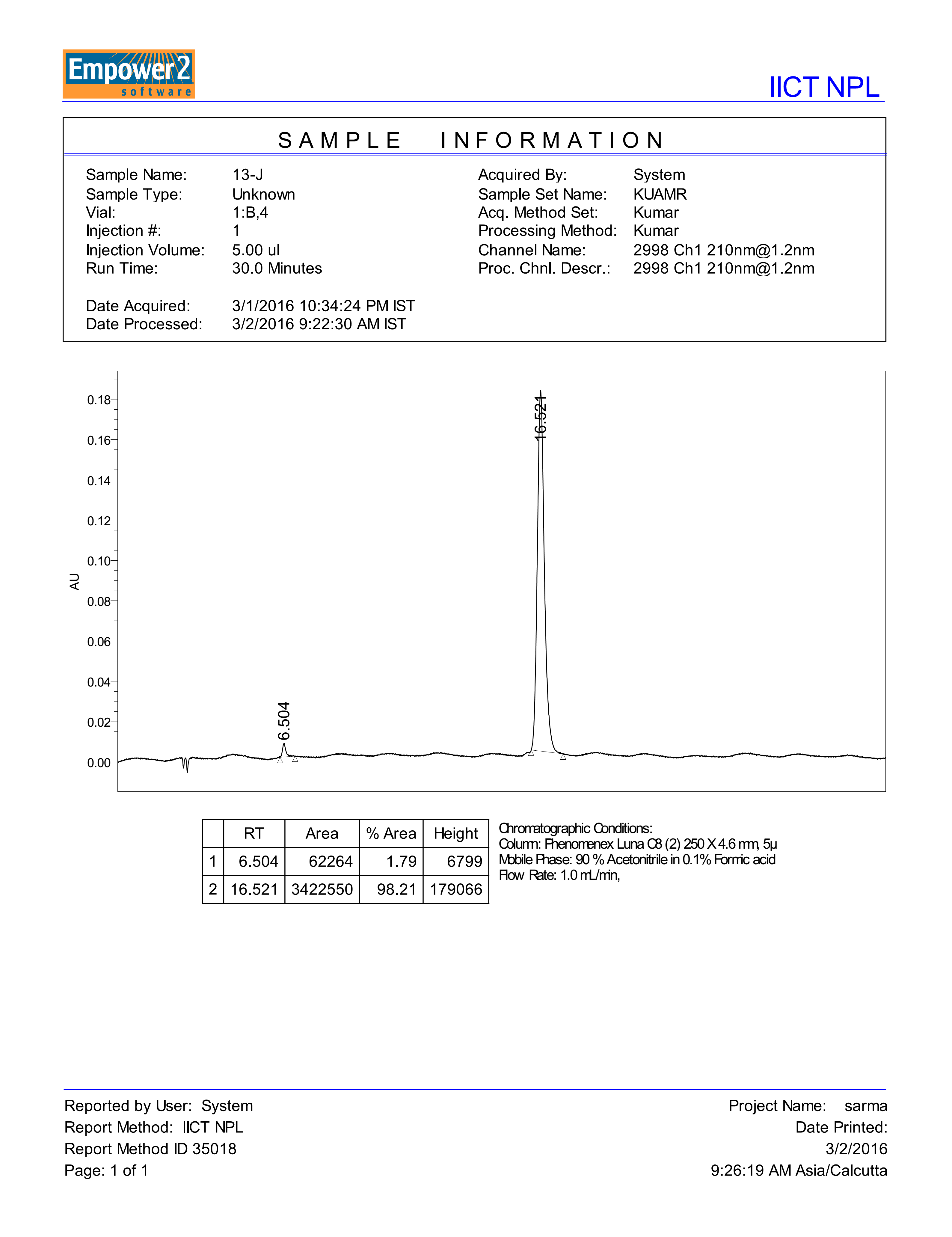


HPLC of compound **13k**
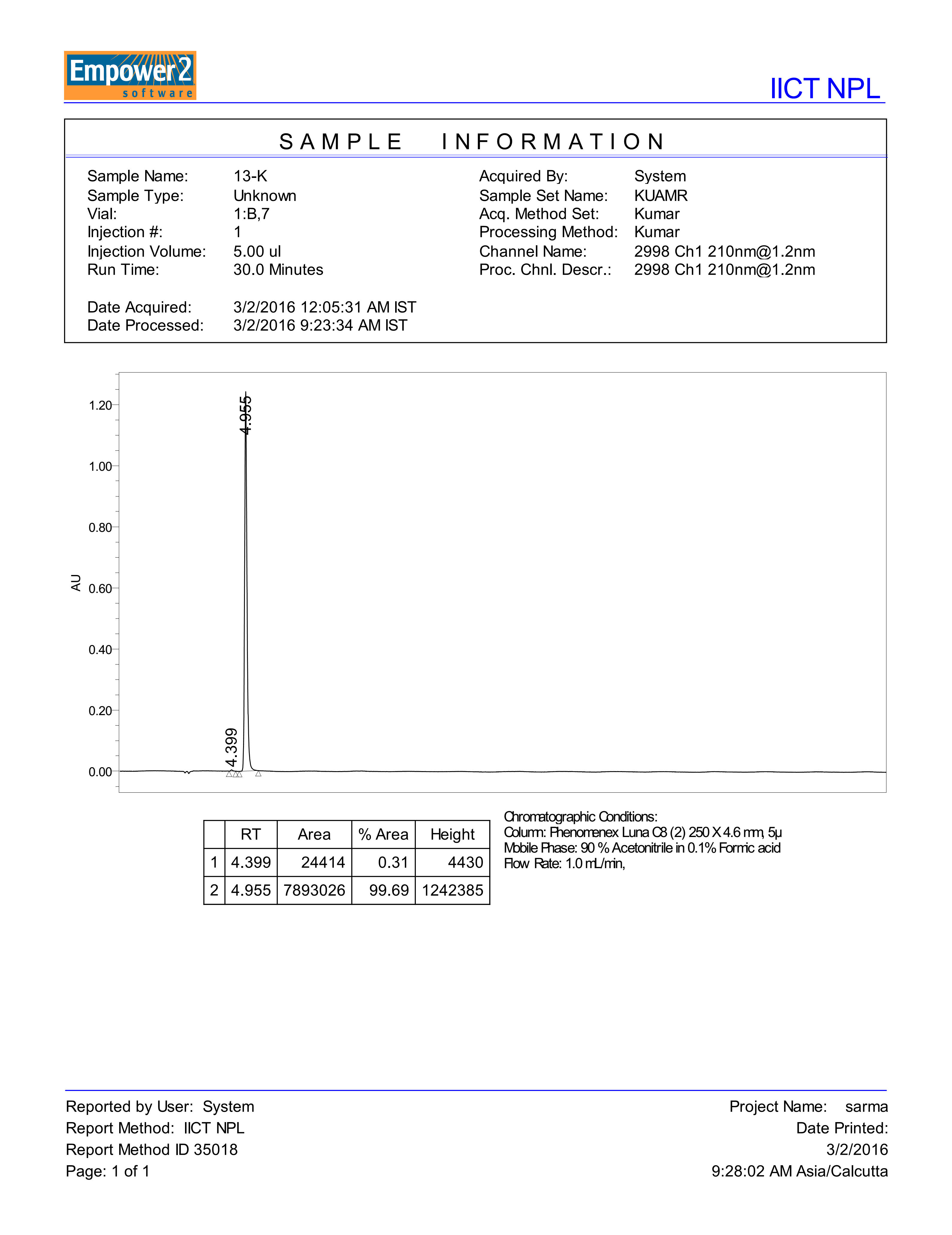


HPLC of compound **13l**
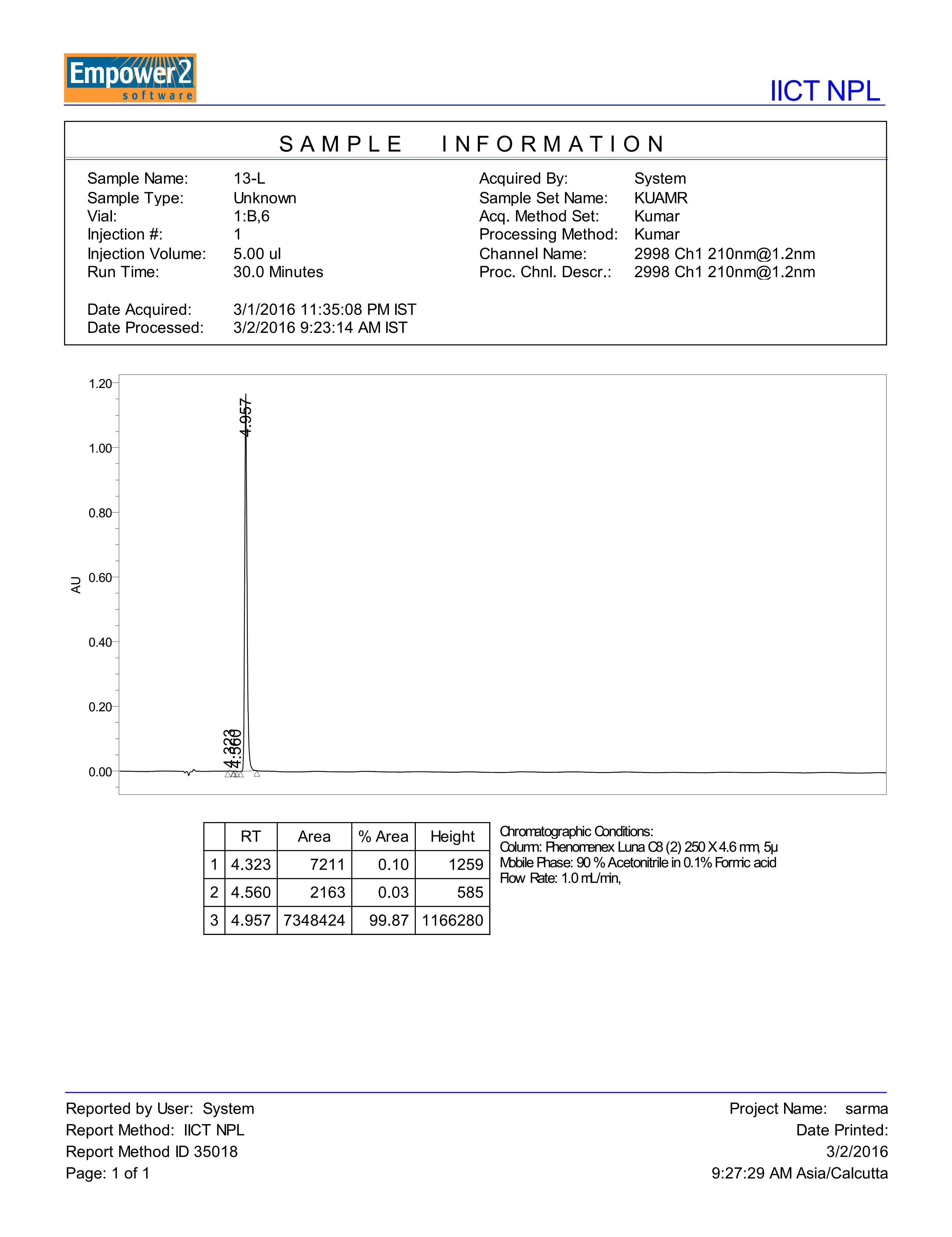


HPLC of compound **13m
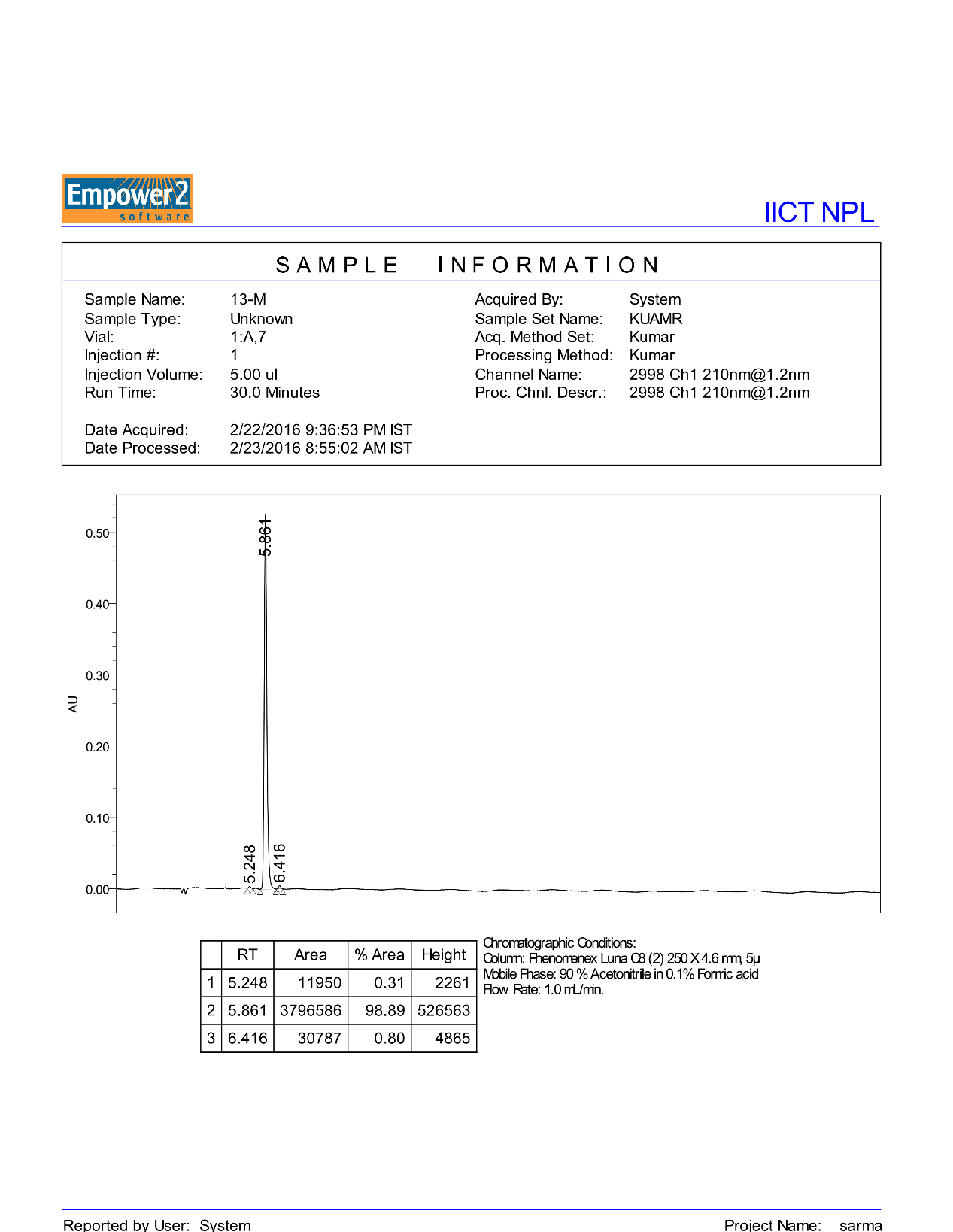
**

HPLC of compound **13n**
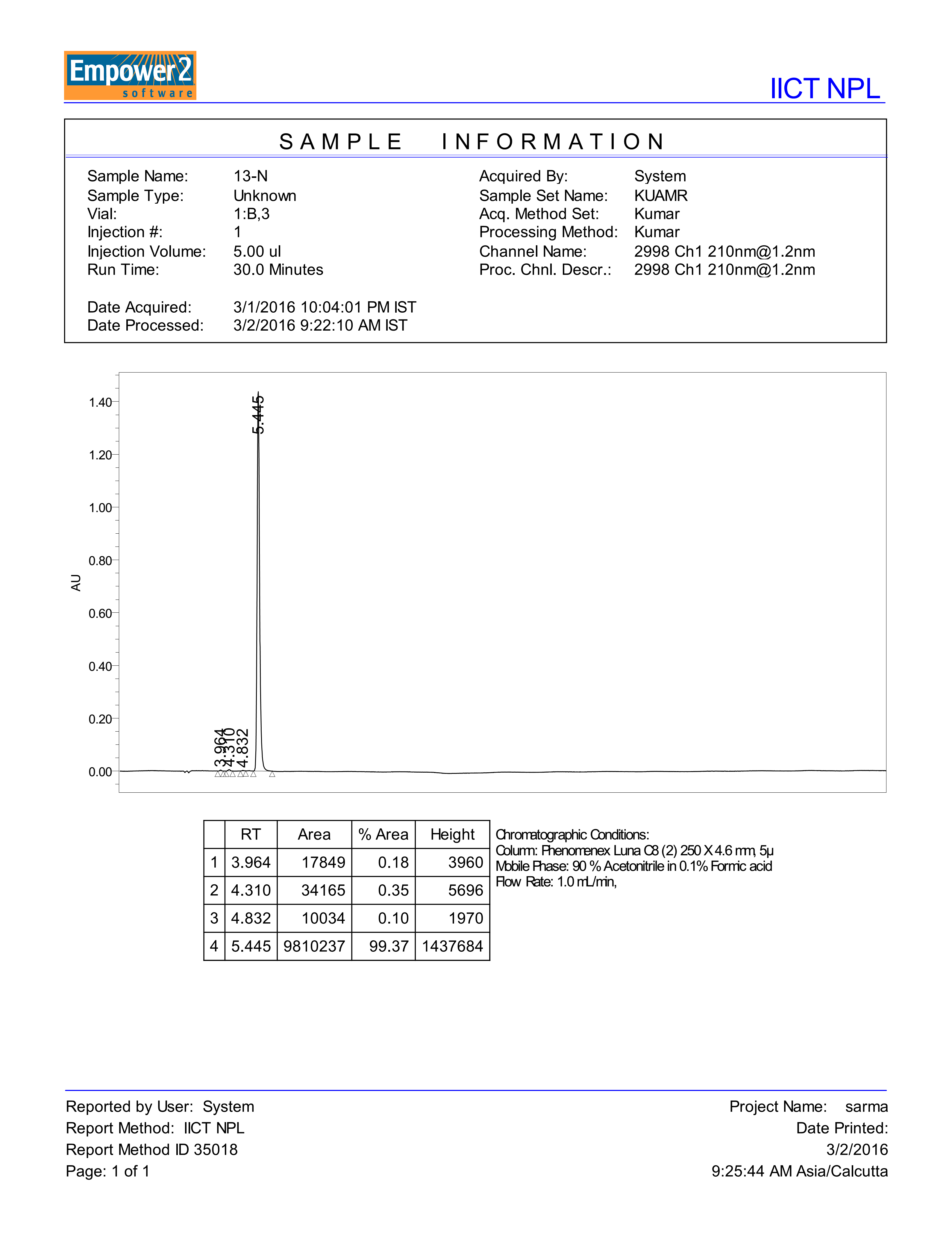


HPLC of compound **13o
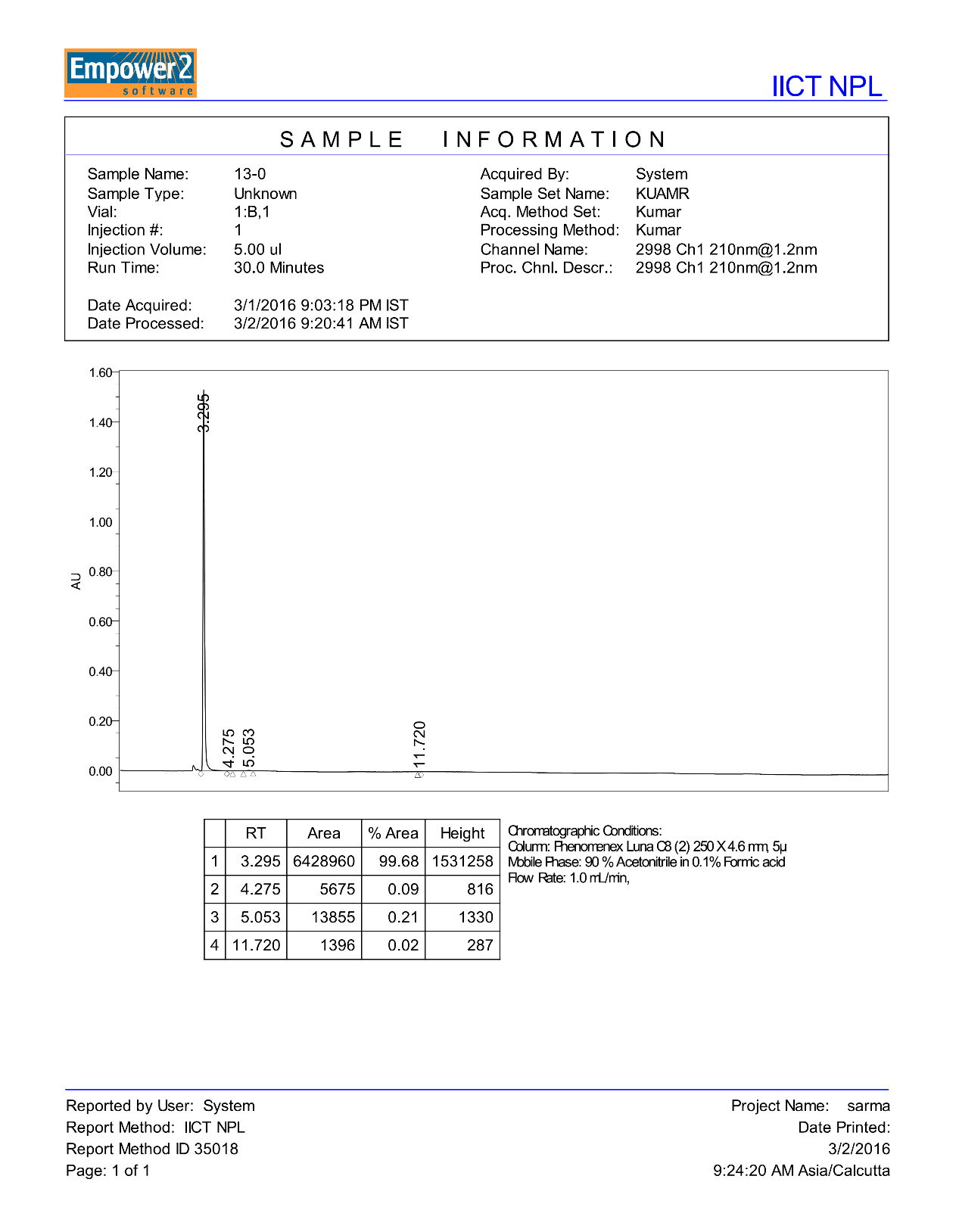
**

HPLC of compound **13q
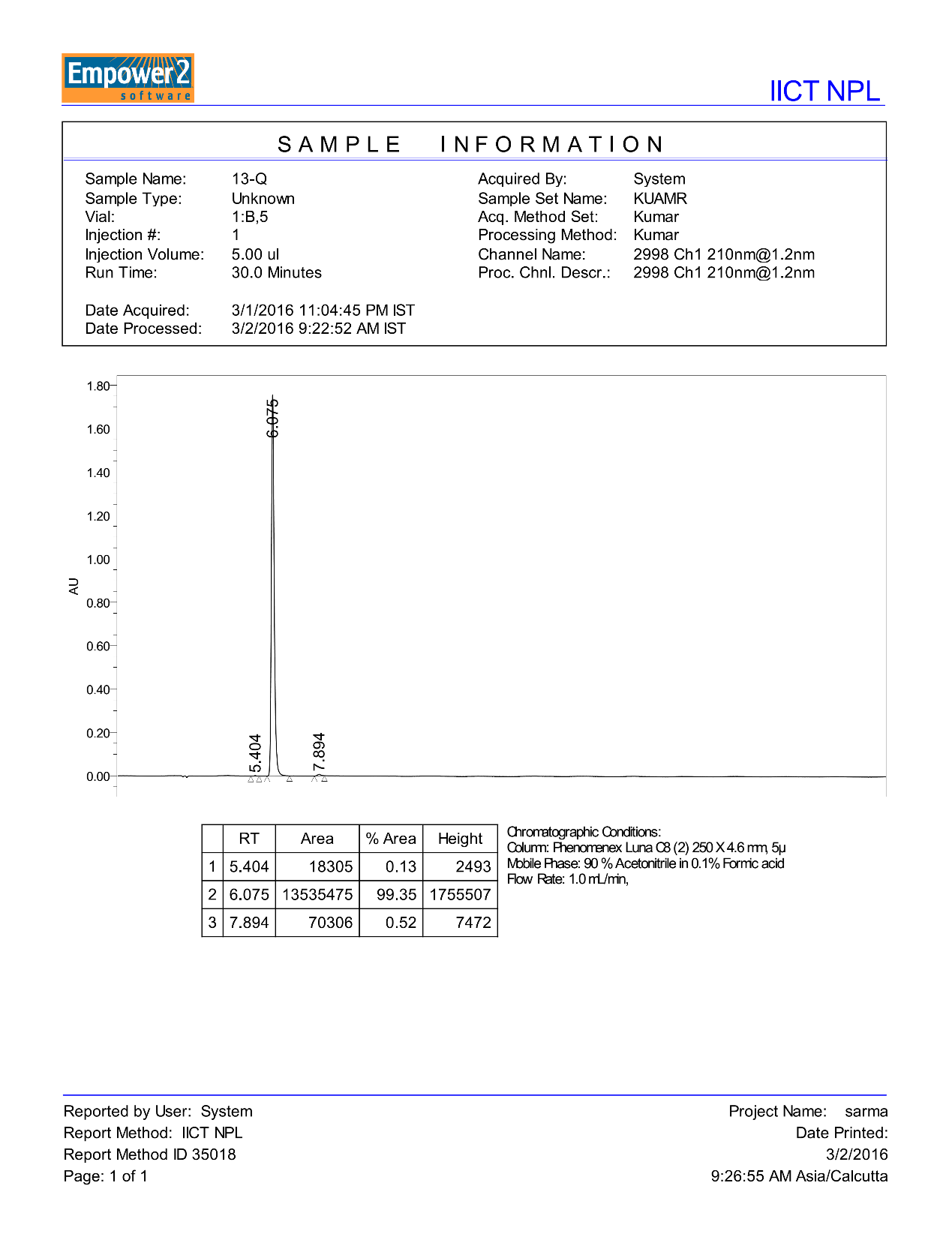
**
